# Supplementary material for: Adeno-associated viral vector resource for the RNA-targeting Cas13d: A comparison of high-fidelity variants, DjCas13d and hfCas13d
Source: Mol Ther Methods Clin Dev. 2025 Aug 20;33(4):101565. doi: 10.1016/j.omtm.2025.101565 (PMC12466237; doi:10.1016/j.omtm.2025.101565)
Supplement: Document S1. Figures S1–S11 [file mmc1.pdf]

OMTM, Volume *33*

Supplemental information

Adeno-associated viral vector resource for the RNA-targeting Cas13d: A comparison of high-fidelity variants, DjCas13d and hfCas13d

Franklin Back, Alfredo Sandoval, Lily M. Vu, Veronica M. Hong, Amulya Bhaskara, Sierra R. Rodriguez, John T. O'Brien, Benedict J. Kolber, Sven Kroener, and Jonathan E. Ploski

`

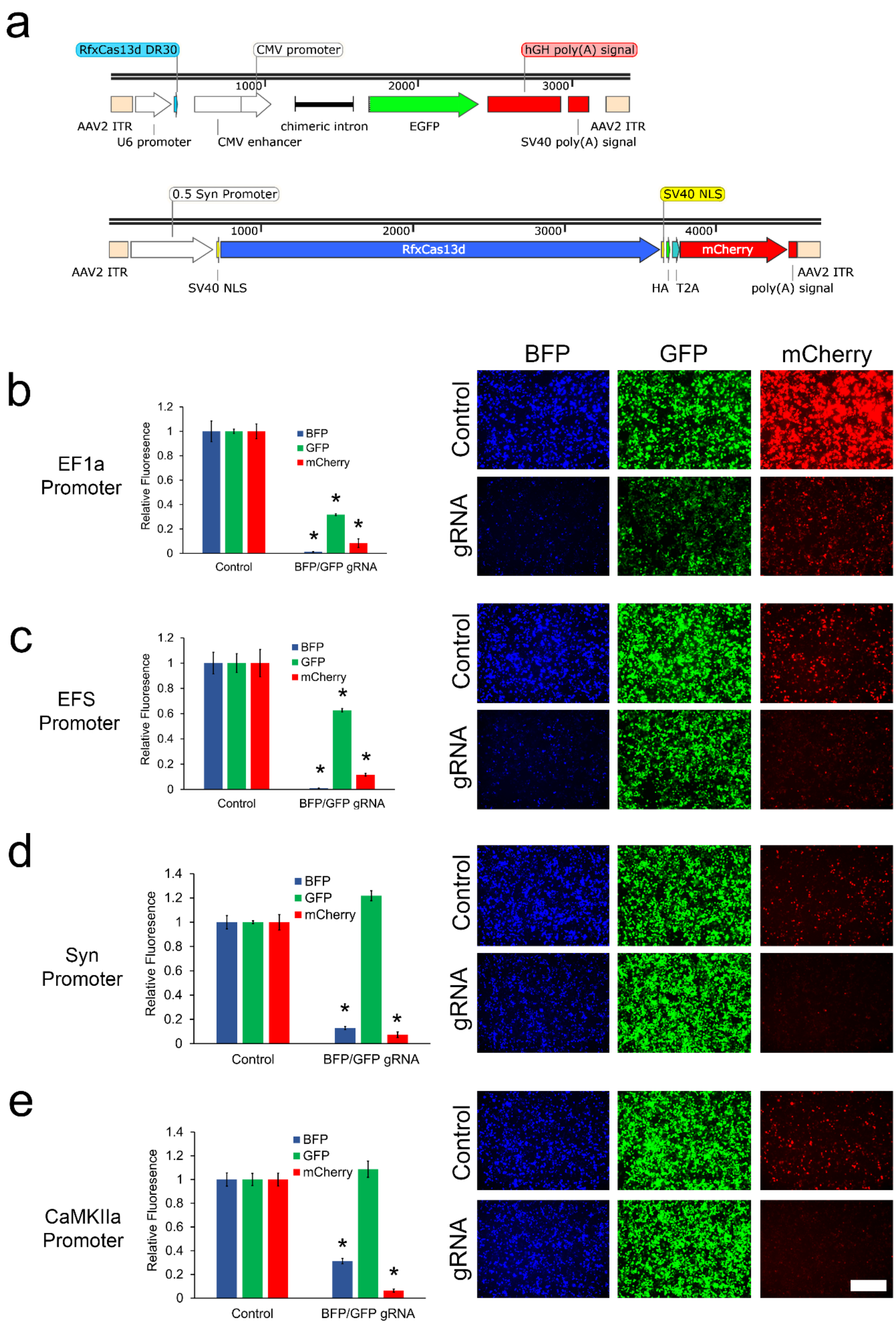
a
RfxCas13d DR30
CMV promoter
hGH poly(A) signal
1000
2000
3000
AAV2 ITR
U6 promoter
CMV enhancer
chimeric intron
EGFP
AAV2 ITR
SV40 poly(A) signal
0.5 Syn Promoter
SV40 NLS
1000
2000
3000
4000
RfxCas13d
mCherry
AAV2 ITR
SV40 NLS
HA
T2A
AAV2 ITR
poly(A) signal
BFP
GFP
mCherry
b
EF1a Promoter
Relative Fluoresence
Control
BFP/GFP gRNA
Control
gRNA
c
EFS Promoter
d
Syn Promoter
e
CaMKIIa Promoter

`

Figure S1: Demonstration of the functionality of AAV-based gRNA and CasRx (dual vector) plasmids in Neuro-2A cells. a.) Schematic of AAV vector maps. The first vector is designed to express a gRNA expression cassette using a U6 promoter and a GFP gene designed to be expressed from a CMV promoter. The second vector is designed to express a CasRx(RfxCas13d)-mCherry gene controlled from a synapsin promoter. Additional vectors were similarly designed to include an EF-1α, EF-1α short (EFS), or an alpha-CaMKII promoter, instead of a synapsin promoter to control CasRx expression (not shown). **b-e.)** Guide RNA plasmids containing a BFP/GFP targeting gRNA or non-targeting gRNA control plasmids were co-transfected with plasmids designed to express CasRx and nuclear-localized BFP, and 48 hours later the cells were imaged for BFP, GFP and mCherry epifluorescence. EF-1α, EFS, Synapsin, and alpha-CaMKII versions are shown respectively in b., c., d., and e. Quantification of data is depicted on the left side, representative images are presented on the right side. BFP/GFP gRNA-treated samples all exhibited a significant reduction in BFP levels compared to controls, but GFP levels were only lowered in experiments using the EF-1α and EFS CasRx plasmids. The levels of mCherry dropped in all BFP/GFP gRNA-treated conditions. Error bars = standard error of the mean. Four biological replicates ($n = 4$) were used for all experimental groups. Asterisk (*) indicates a significant difference compared to the respective control group (t-test, two-tailed, * = $p < 0.003$). Scale bar = 400 microns. Note: The EF-1α-CasRx-mCherry AAV vector genome is above the recommended packaging limit.

`

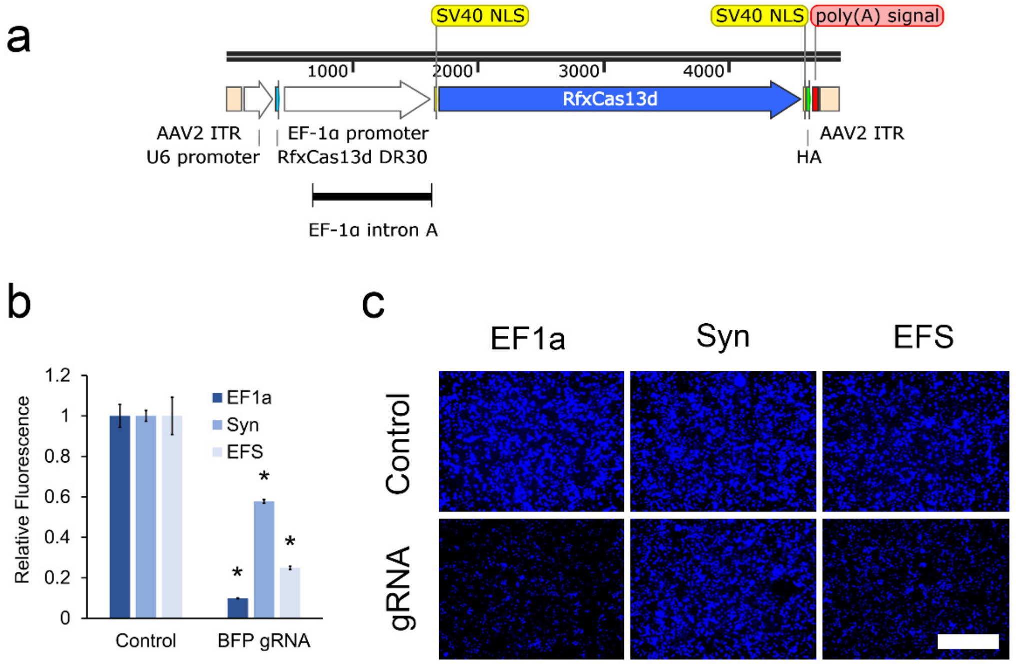

Figure S2: Demonstration of the functionality of AAV-based gRNA/CasRx (single vector) plasmids in Neuro-2A cells. a.) Schematic of AAV vector map designed to express a gRNA expression cassette using a U6 promoter and a CasRx(RfxCas13d) gene controlled from an EF-1α promoter. Additional vectors were similarly designed to include an EF-1α short (EFS) or synapsin promoter, instead of an EF-1α promoter to control CasRx expression (not shown). **b-c.)** Each of these single vector plasmids containing a BFP targeting gRNA or non-targeting gRNA control were co-transfected with plasmids designed to express nuclear-localized BFP, and 48 hours later the cells were imaged for BFP epifluorescence. Quantification of data depicted in b.), and representative images presented in c.). BFP gRNAs treated samples all exhibited a significant reduction in BFP levels compared to controls. Error bars = standard error of the mean. Four biological replicates ($n = 4$) were used for all experimental groups. Asterisk (*) indicates a significant difference compared to the respective control group (t-test, two-tailed, * = $p < 0.0002$). Scale bar = 450 microns.

`

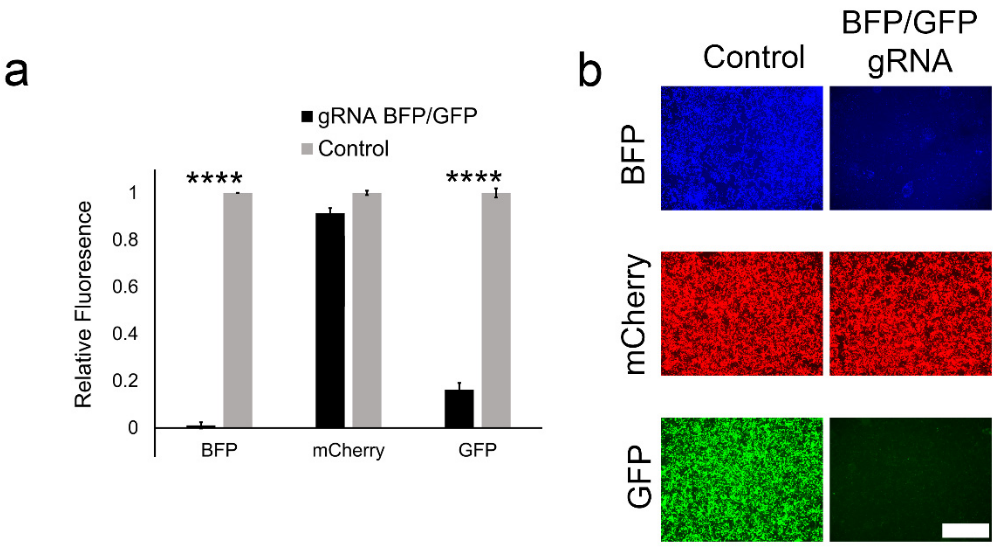

Figure S3: BFP/GFP targeting gRNA is not targeting mCherry. To ensure the BFP/GFP targeting gRNA is not also targeting mCherry, we co-transfected 293FT cells with a vector containing an EFS-CasRx gene, a BFP/GFP gRNA expression cassette, and either BFP, GFP, or mCherry encoding plasmids. Forty-eight hours later, the cells were imaged for BFP/GFP/mCherry expression. BFP and GFP fluorescence were significantly lower in samples that received the BFP/GFP targeting gRNA compared to control samples that received a non-targeting gRNA while mCherry levels remained high, indicating that the gRNA was not targeting mCherry. Data quantitation is depicted in a.) and representative images are depicted in b.). All groups contained 4-6 biological replicates ($n = 4\text{-}6$). T-test, two-tailed, **** = $p < 1E\text{-}9$ compared to the respective control group. Error bars = standard error of the mean. Scale bar = 400 microns.

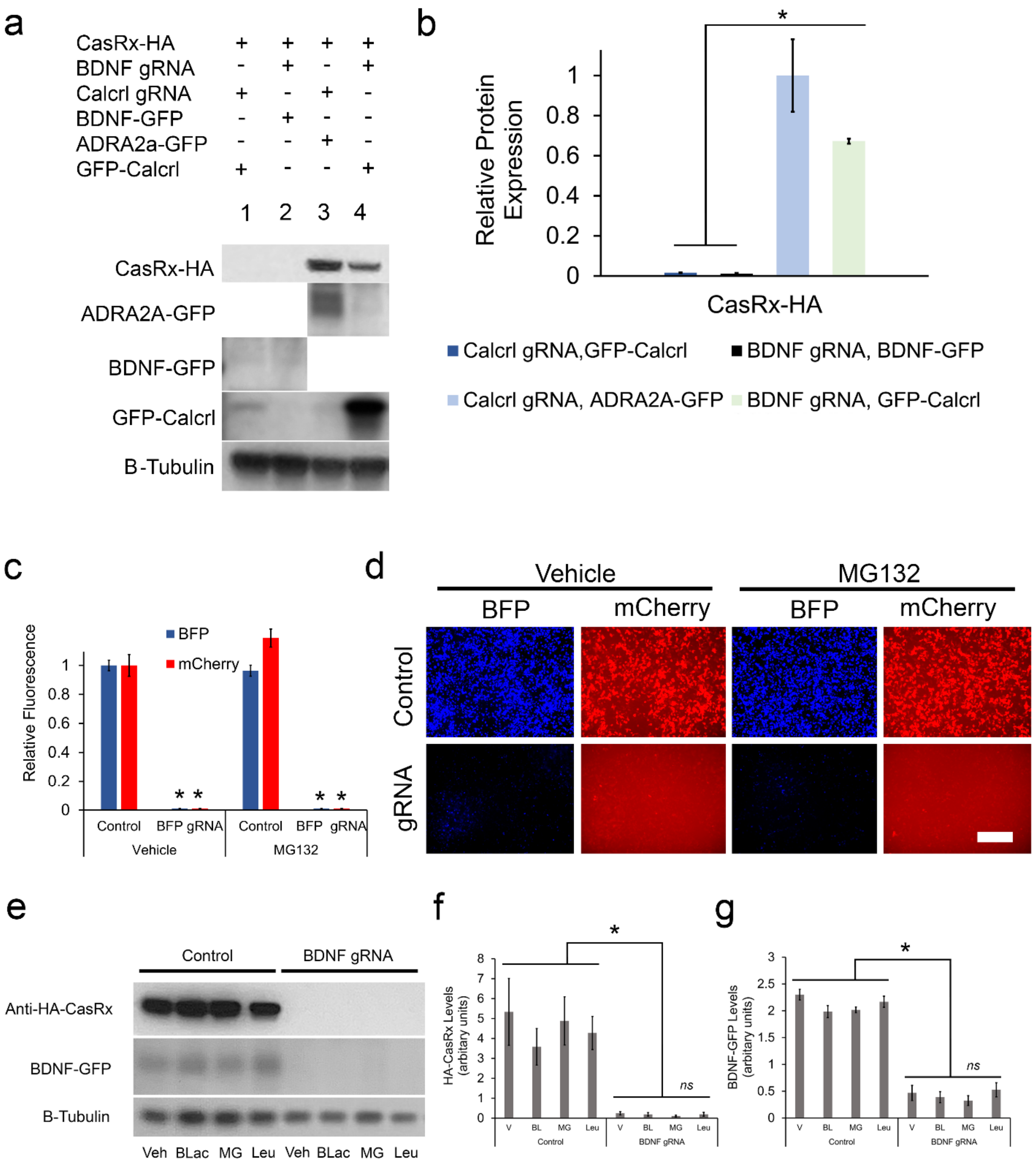
a
CasRx-HA + + + +
BDNF gRNA - + - +
Calcrl gRNA + - + -
BDNF-GFP - + - -
ADRA2a-GFP - - + -
GFP-Calcrl + - - +
1 2 3 4
CasRx-HA
ADRA2A-GFP
BDNF-GFP
GFP-Calcrl
B-Tubulin
b
*
Relative Protein Expression
0 0.2 0.4 0.6 0.8 1
CasRx-HA
Calcrl gRNA,GFP-Calcrl
BDNF gRNA, BDNF-GFP
Calcrl gRNA, ADRA2A-GFP
BDNF gRNA, GFP-Calcrl
c
Relative Fluorescence
0 0.2 0.4 0.6 0.8 1
BFP
mCherry
* *
* *
Control BFP gRNA Control BFP gRNA
Vehicle MG132
d
Vehicle
MG132
BFP mCherry BFP mCherry
Control
gRNA
e
Control
BDNF gRNA
Anti-HA-CasRx
BDNF-GFP
B-Tubulin
Veh BLac MG Leu Veh BLac MG Leu
f
*
HA-CasRx Levels (arbitary units)
0 1 2 3 4 5 6 7 8
ns
V BL MG Leu V BL MG Leu
Control BDNF gRNA
g
*
BDNF-GFP Levels (arbitary units)
0 0.5 1 1.5 2 2.5 3
ns
V BL MG Leu V BL MG Leu
Control BDNF gRNA

`

Figure S4: CasRx protein levels become significantly depleted when CasRx is co-expressed with a gRNA and its intended target and this is not influenced by the ubiquitin-proteasome system. a-b.) 293FT cells were transfected with plasmids encoding EFS-CasRx, gRNAs designed to target *BDNF*, or *Calcrl*, and plasmids designed to express rat BDNF-GFP, ADRA2A-GFP, or GFP-*Calcrl*. Forty-eight hours later, the cells were harvested and examined via western blotting for CasRx, ADRA2A-GFP and GFP-*Calcrl* protein levels. When *Calcrl* gRNAs and GFP-*Calcrl* (lane 1) or *BDNF* gRNAs and BDNF-GFP (lane 2), were co-expressed, CasRx protein levels were significantly lowered. However, CasRx levels remained high when *Calcrl* gRNAs and ADRA2A-GFP (lane 3) or *BDNF* gRNAs and GFP-*Calcrl* (lane 4), were co-expressed. CasRx protein levels were significantly depleted when CasRx was co-expressed with a gRNA and its intended target (ANOVA with Tukey pairwise comparisons, * = $p < 0.004$). Note that BDNF protein is not present in lane two because it is degraded. Each group contained 3 biological replicates (n = 3). Error bars = standard error of the mean. HA-CasRx and GFP protein levels were normalized to Beta-Tubulin levels for each sample. **(c-d).** 293FT cells were transfected with plasmids encoding CasRx-mCherry, BFP, and BFP gRNA expression cassettes or control gRNA expression cassettes, and the cells were either treated with vehicle or the ubiquitin-proteasome inhibitor, MG132. Forty-eight hours later, the cells were imaged for BFP and mCherry epifluorescence. BFP and mCherry fluorescence dropped in samples that were treated with BFP gRNAs, but not controls, and the treatment with MG132 had no influence on these levels. Two-way ANOVA revealed a significant effect for BFP gRNA on BFP and mCherry levels (* = $p < 1E-9$), however, there was no interaction for MG132 treatment ($p > 0.05$). Quantified data are shown in c.) and representative images are depicted in d.). Four biological replicates for all groups (n = 4). Scale bar = 375 microns. **(e-g)**. 293FT cells were transfected with plasmids encoding CasRx, BDNF-GFP, and *BDNF* gRNAs or control gRNAs expression cassettes and treated cells with either vehicle (Veh or V) or the ubiquitin-proteasome inhibitors, MG132 (MG), or clasto-Lactacystin β-lactone (BLac or BL) or the lysosomal inhibitor leupeptin (Leu). Forty-eight hours later, the cells were harvested, and protein levels for CasRx and BDNF-GFP were examined via western blotting. CasRx and BDNF-GFP levels were significantly lowered in the cells that were treated with *BDNF* gRNAs compared to controls (Two-way ANOVA, * $p < 0.0000003$), and none of the inhibitors influenced these protein levels (Two-way ANOVA $p > 0.3$ = *ns*). Four biological replicates for all groups (n = 4). Error bars = standard error of the mean.

`

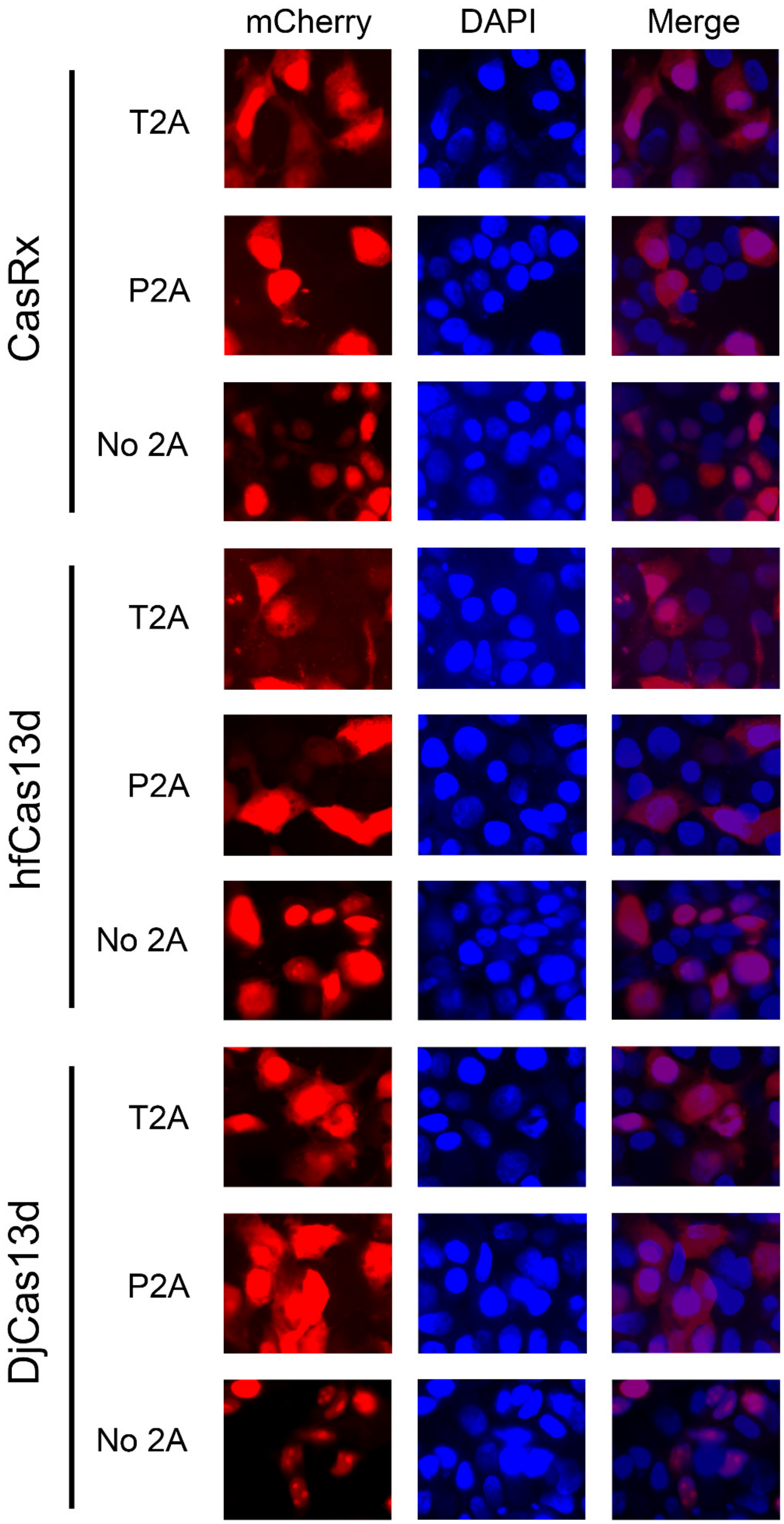

mCherry
DAPI
Merge
CasRx
T2A
P2A
No 2A
hfCas13d
T2A
P2A
No 2A
DjCas13d
T2A
P2A
No 2A

`

Figure S5: Cas13d/mCherry protein cellular localization. Plasmids designed to encode three different variants of Cas13d that contain either a T2A-mCherry sequence, a P2A-mCherry sequence or don't have a 2A sequence, but are designed to be a fusion protein with mCherry, were transfected into 293FT cells. Forty-eight hours later the cells were imaged for mCherry and DAPI epifluorescence to observe the cellular localization of mCherry. From left to right, the panels are mCherry, DAPI and the merged signal between the two to clearly indicate the cellular localization of mCherry. In all cases there is strong nuclear localization of mCherry. For samples that contain 2A sequences, there is also significant cytoplasmic localization of mCherry. Samples that do not contain 2A sequences but are instead Cas13-Cherry fusions, show virtually only nuclear localization except in cases of high expression. Representative images are shown for all samples.

`

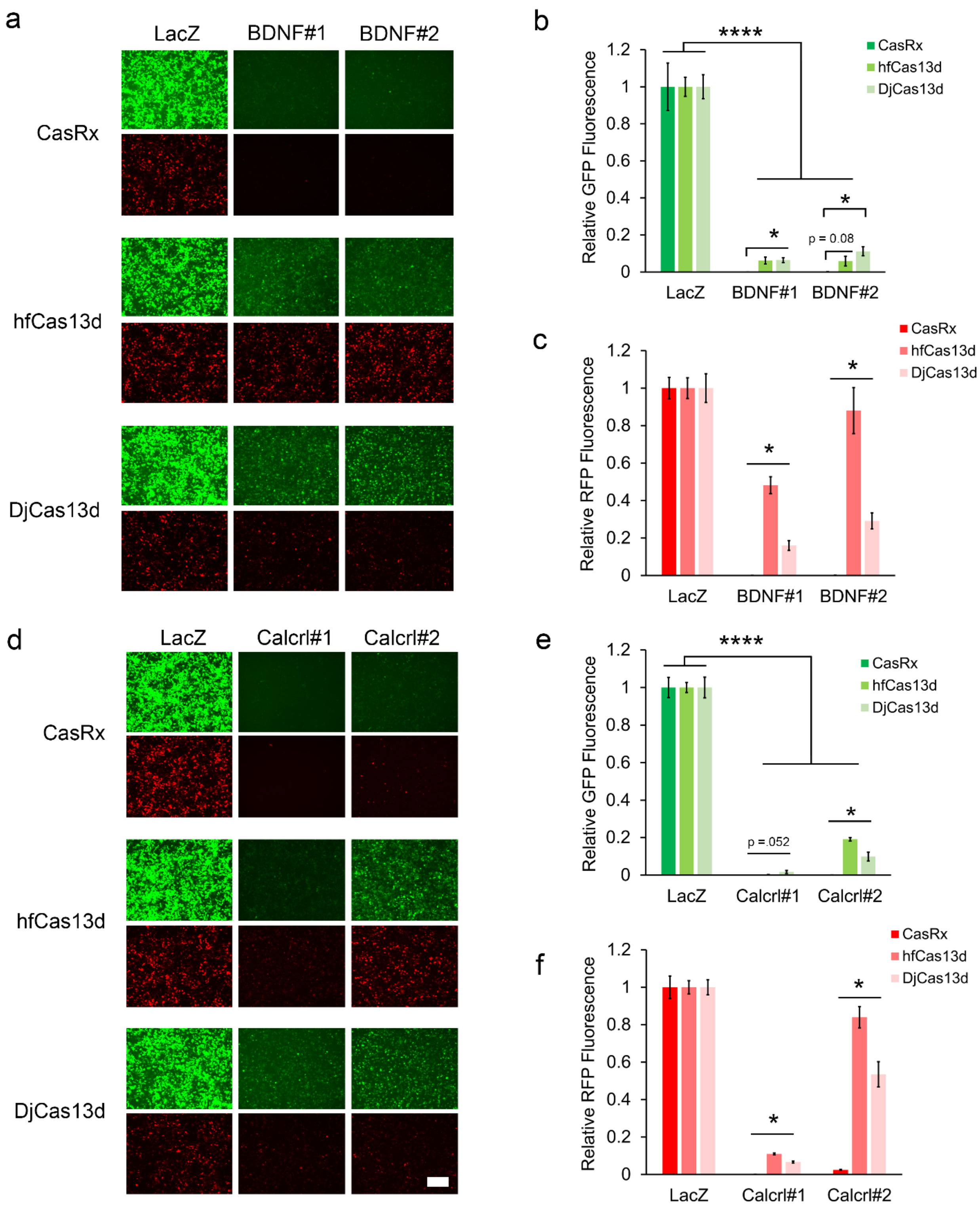

Figure S6: Variants of Cas13d knock down ectopically-expressed genes efficiently. (a). 293FT cells were transfected with Cas13d encoding vectors, a GFP-*BDNF* encoding plasmid and two different gRNA expression cassettes, *BDNF* 1 and 2, or a gRNA expression cassette designed to target LacZ as a control. Forty-eight hours post-transfection the cells were imaged for GFP and mCherry(RFP) epifluorescence. Representative images are shown. **(b).** Quantification of

`

images revealed that treatment with both *BDNF* gRNAs resulted in robust knockdown of GFP-*BDNF* for each of the Cas13d variants compared to LacZ controls (**** = ANOVA, $p < 0.000006$; Fisher's LSD, $p < 0.000005$). There was a significant difference in GFP-*BDNF* knockdown for *BDNF* 1 among the Cas13d variants, with CasRx outperforming the other Cas13d variants (* = ANOVA, $p = 0.0103$; Fisher's LSD, $p < 0.008059$). There was a significant difference in GFP-*BDNF* knockdown for *BDNF* 2 between CasRx and DjCas13d, (* = ANOVA, $p < 0.0134$; Fisher's LSD, $p = 0.0042$). Four biological replicates for all groups ($n = 4$). **(c).** Both DjCas13d and CasRx groups, for both *BDNF* 1 and 2, exhibited a significant reduction in RFP levels compared to LacZ controls, (ANOVA, $p < 0.000003$; Fisher's LSD, $p < 0.000006$), however, RFP levels were only significantly different from LacZ controls for hfCas13d *BDNF* 1 (ANOVA, $p = 0.003991$; Fisher's LSD, $p < 0.001598$), but not hfCas13d *BDNF* 2 ($p = 0.3296$). RFP levels for the hfCas13d, *BDNF* 1 and 2 groups were significantly higher than the DjCas13d and CasRx, *BDNF* 1 and 2 groups (* = ANOVA, $p < 0.000055$; Fisher's LSD, $p < 0.023700$). Four biological replicates for all groups ($n = 4$). **(d).** 293FT cells were transfected with either of these Cas13d encoding vectors, a GFP-*Calcrl* encoding plasmid and two different gRNA expression cassettes, *Calcrl* 1 and 2, or a gRNA expression cassette designed to target LacZ as a control. Forty-eight hours post transfection the cells were imaged for GFP and mCherry(RFP) epifluorescence. Representative images shown. **(e).** Quantification of images revealed that treatment with both *Calcrl* gRNAs resulted in robust knockdown of GFP-*Calcrl* for each of the Cas13d variants compared to LacZ controls (**** = ANOVA, $p < 1.39E-08$; Fisher's LSD, $p < 1.96E-08$). There was not a significant difference in GFP-*Calcrl* knockdown for *Calcrl* 1 among the Cas13d variants, (ANOVA, $p = 0.052$). There was a significant difference in GFP-*Calcrl* knockdown for *Calcrl* 2 among the Cas13d variants, with CasRx outperforming the other Cas13d variants (* = ANOVA, $p = 0.000024$; Fisher's LSD, $p < 0.000966$). The use of DjCas13d led to a significant knockdown of GFP-*Calcrl* compared to hfCas13d ($p < 0.001449$). Four biological replicates for all groups ($n = 4$). **(f).** Each of the Cas13d variants, for both *Calcrl* 1 and 2, exhibited a significant reduction in RFP levels compared to LacZ controls, (** = ANOVA, $p < 0.000001$; Fisher's LSD, $p < 0.016729$). RFP levels for the hfCas13d, *Calcrl* 1 and 2 groups were significantly higher than the DjCas13d and CasRx, *Calcrl* 1 and 2 groups (* = ANOVA, $p < 0.000004$; Fisher's LSD, $p < 0.002160$). Four biological replicates for all groups ($n = 4$). Error bars = standard error of the mean. Scale bar = 325 microns.

`

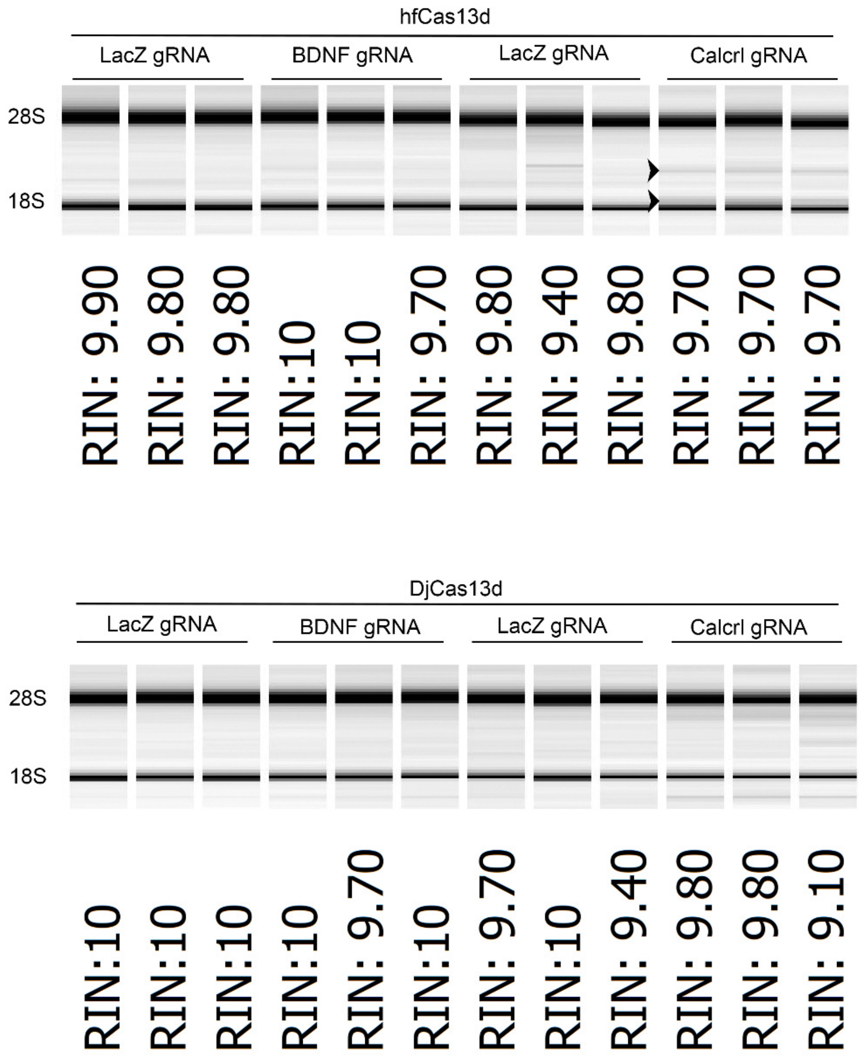

Figure S7:

RNA analysis via Agilent bioanalyzer for total RNA samples that were used in experiments depicted in Figures S6 and Figure 4. Arrows point to the two degraded RNA bands seen in the hfCas13d/*Calcrl* gRNA samples. No other samples show this stereotyped pattern of RNA degradation. These findings are consistent with the data presented in Figure 3. RNA Integrity Numbers (RINs) are shown for each sample.

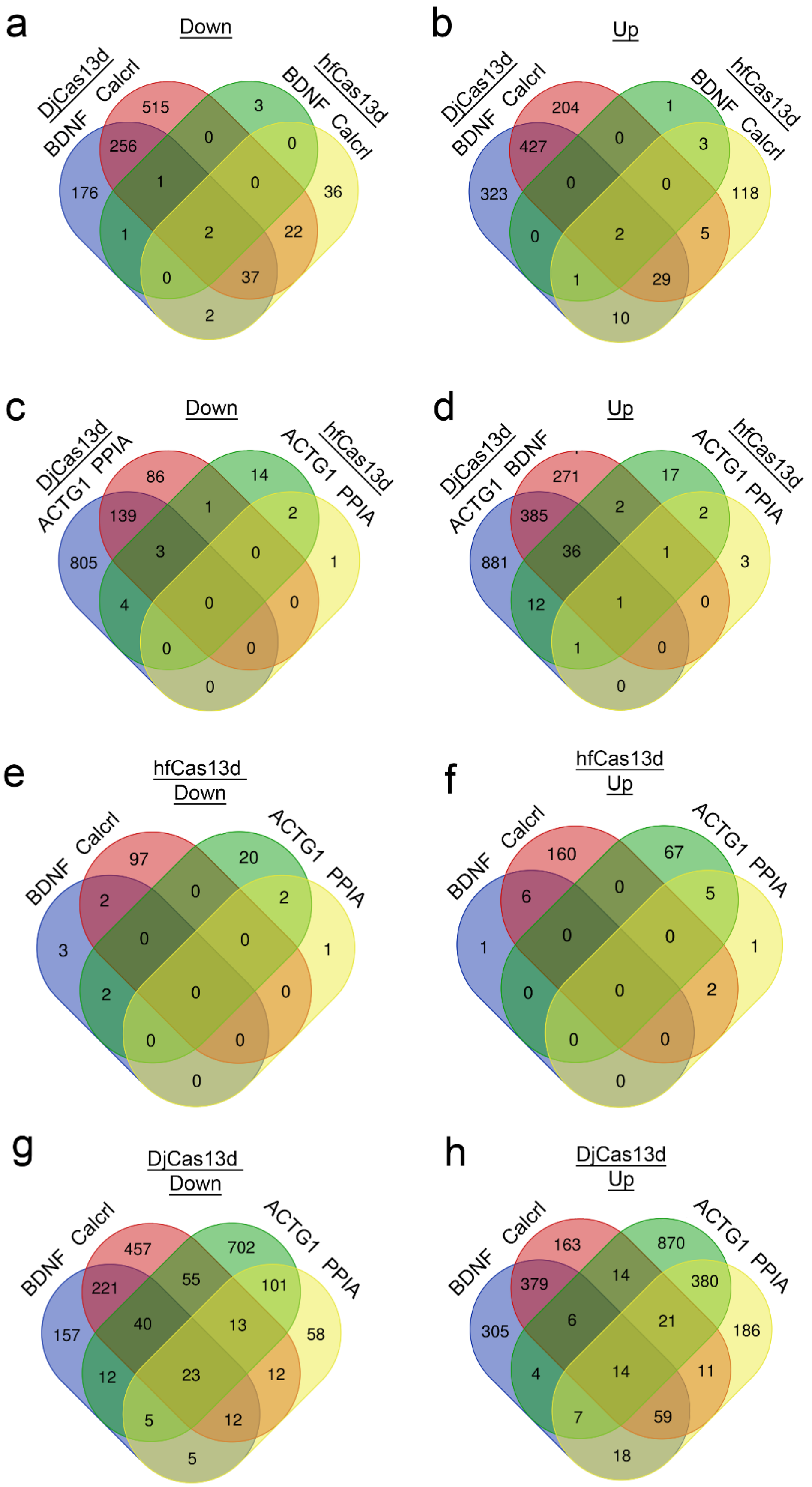
a
Down
DjCas13d
BDNF Calcrl
hfCas13d
BDNF Calcrl
515
3
256
0
0
176
1
0
36
1
2
22
0
37
2
b
Up
DjCas13d
BDNF Calcrl
hfCas13d
BDNF Calcrl
204
1
427
0
3
323
0
0
118
0
2
5
1
29
10
c
Down
DjCas13d
ACTG1 PPIA
hfCas13d
ACTG1 PPIA
86
14
139
1
2
805
3
0
1
4
0
0
0
0
0
d
Up
DjCas13d
ACTG1 BDNF
hfCas13d
ACTG1 PPIA
271
17
385
2
2
881
36
1
3
12
1
0
1
0
0
e
hfCas13d
Down
BDNF Calcrl
ACTG1 PPIA
97
20
2
0
2
3
0
0
1
2
0
0
0
0
0
f
hfCas13d
Up
BDNF Calcrl
ACTG1 PPIA
160
67
6
0
5
1
0
0
1
0
0
2
0
0
0
g
DjCas13d
Down
BDNF Calcrl
ACTG1 PPIA
457
702
221
55
101
157
40
13
58
12
23
12
5
12
5
h
DjCas13d
Up
BDNF Calcrl
ACTG1 PPIA
163
870
379
14
380
305
6
21
186
4
14
11
7
59
18

`

Figure S8: Venn diagrams illustrating the similarities and differences of dysregulated genes among several RNAseq experiments.

(a). Venn diagram showing the overlap of downregulated genes from 4 RNAseq experiments (see Figure 4), designed to target GFP-*BDNF* or GFP-*Calcrl* with either DjCas13d or hfCas13d. (**b.**) Venn diagram showing the overlap of upregulated genes from 4 RNAseq experiments (see Figure 4), designed to target GFP-*BDNF* or GFP-*Calcrl* with either DjCas13d or hfCas13d. **(c).** Venn diagram showing the overlap of downregulated genes from 4 RNAseq experiments (see Figure 5), designed to target *ACTG1* or *PPIA* with either DjCas13d or hfCas13d. (**d.**) Venn diagram showing the overlap of upregulated genes from 4 RNAseq experiments (see Figure 5), designed to target *ACTG1* or *PPIA* with either DjCas13d or hfCas13d. **(e, f).** Venn diagrams showing the overlap of down and upregulated genes from 4 RNAseq experiments (see Figures 4 and 5), designed to target GFP-*BDNF*, GFP-*Calcrl*, *ACTG1* or *PPIA* with hfCas13d. **(g, h).** Venn diagrams showing the overlap of down and upregulated genes from 4 RNAseq experiments (see Figures 4 and 5), designed to target GFP-*BDNF*, GFP-*Calcrl*, *ACTG1* or *PPIA* with DjCas13d.

`

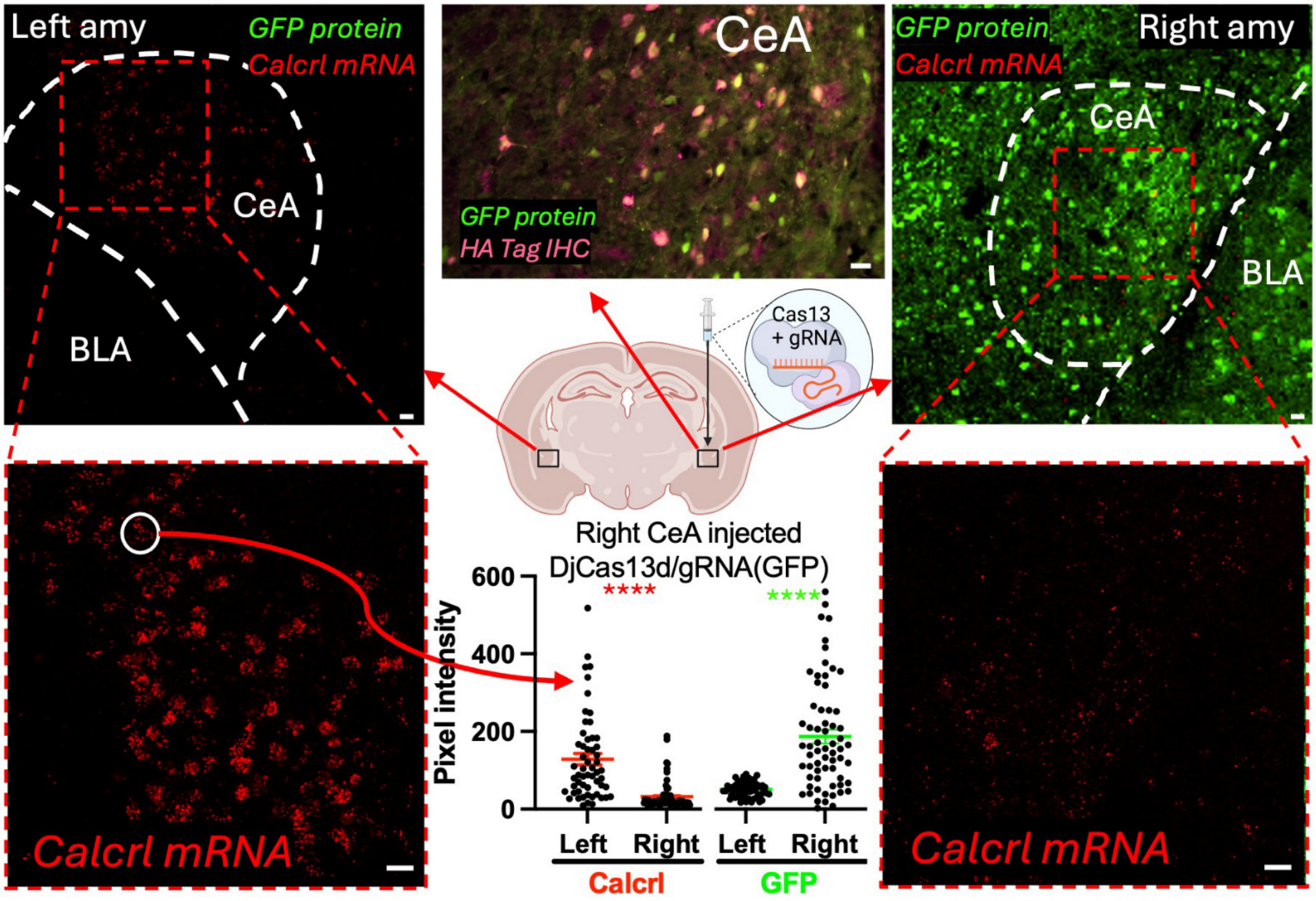

Figure S9: In Vivo Disruption of *Calcrl* mRNA with DjCas13d in mice. The right central amygdala (CeA) of a mouse was injected with AAV-DjCas13d-HA and AAV-gRNA*Calcrl*-GFP. Central top image shows >85% co-localization of GFP (gRNA vector) and HA tag IHC (DjCas13d protein). The amount of *Calcrl* mRNA was decreased in the right CeA (bottom right 400 x image) compared to the uninjected left CeA (bottom left 400 x image) as quantified in the bottom central graph. Quantification was completed on all cells in a representative image for *Calcrl* and for GFP (tagged to gRNA). Scale bars = 20um ****P<0.0001 t-test between sides.

`

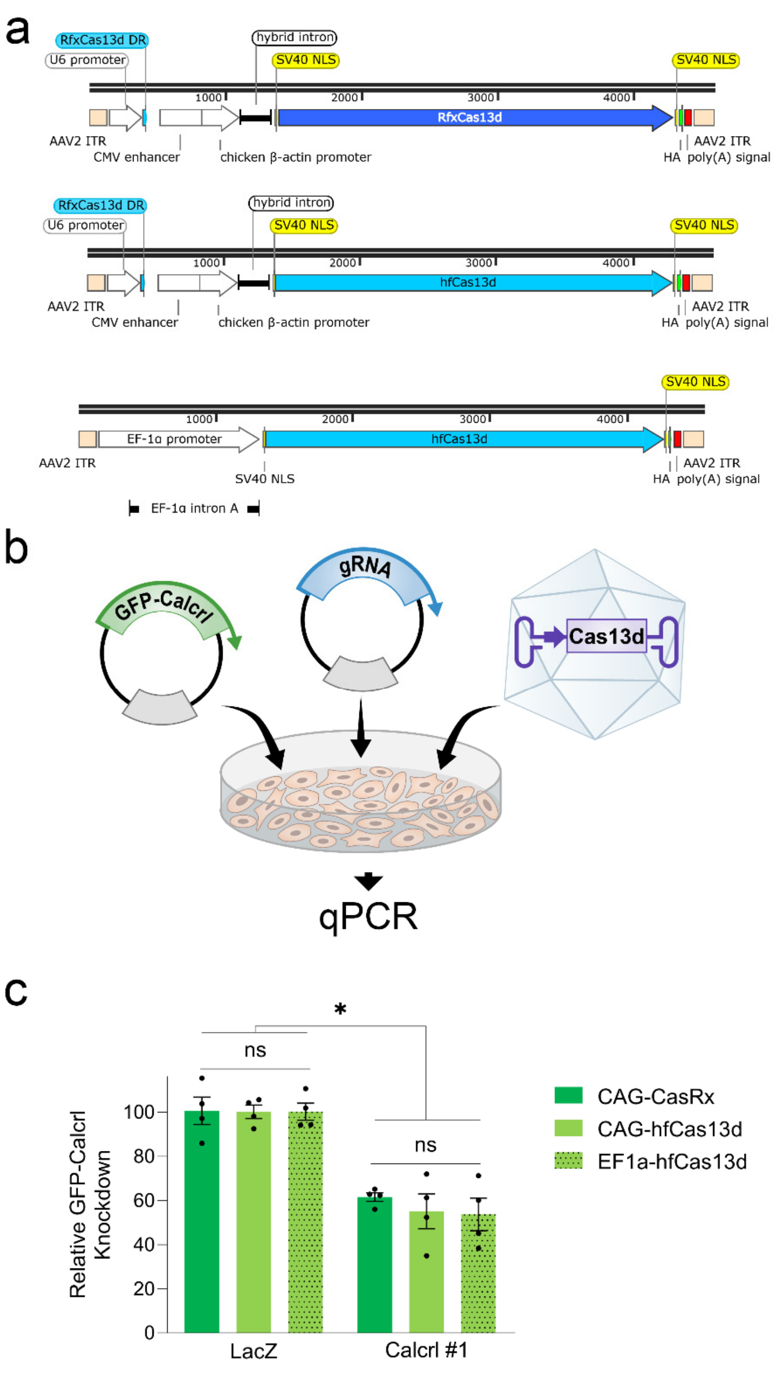

Figure S10: AAVs encoding Cas13d show functionality with different promoters

(a). Schematic of AAV vector maps depicting AAVs designed to express CasRx and hfCas13d, from a CAG promoter. These vectors also contain a gRNA expression cassette upstream of the Cas13d gene. The bottom vector is an AAV designed to express hfCas13d from an Ef1a promoter. This vector does not have a gRNA expression cassette. **(b,c).** Graphic describing the outline of the experiment. 293FT cells were transfected with a GFP-*Calcrl* encoding plasmid and a gRNA expression cassette, *Calcrl* 1, or a gRNA expression cassette designed to target LacZ as a control. The cells were also transduced with Cas13d encoding viruses depicted in (a.). Seventy-two hours post-transfection/transduction the samples were processed for qPCR to examine GFP mRNA levels. Quantitative PCR revealed GFP was significantly knocked down compared to the LacZ control group demonstrating the functionality of these viruses, two-way ANOVA (* = $p < 0.05$). Error bars = standard error of the mean.

`

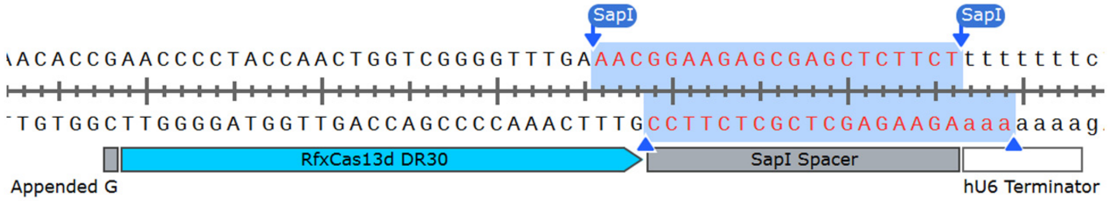

gRNA Name	Sequence (5' - 3')
GFP/BFP Top	aacTTGTACTCCAGCTTGTGCCCCAG
GFP/BFP Bot	aaaCTGGGGCACAAGCTGGAGTACAA
BDNF1 1 Top	aacAATAACCATAGTAAGGAAAAGGA
BDNF1 1 Bot	aaaTCCTTTTCCTTACTATGGTTATT
BDNF1 2 Top	aacACGTGCTCAAAAGTGTCAGCCAG
BDNF1 2 Bot	aaaCTGGCTGACACTTTTGAGCACGT
Calcrl 1 Top	aacGTAACATTCATATTGAGCCGTCA
Calcrl 1 Bot	aaaTGACGGCTCAATATGAATGTTAC
Calcrl 2 Top	aacCAAAGCAGCACAAATCGGACCAT
Calcrl 2 Bot	aaaATGGTCCGATTTGTGCTGCTTTG
ACTG1 Top	aacACTTCAGGGTCAGGATGCCACGCTTGCTCT
ACTG1 Bot	aaaAGAGCAAGCGTGGCATCCTGACCCTGAAGT
PPIA Top	aacAAACACCACATGCTTGCCATCCAACCACTC
PPIA Bot	aaaGAGTGGTTGGATGGCAAGCATGTGGTGTTT
LacZ Top	aacCGTCTGGCCTTCCTGTAGCCAGCTTTCATC
LacZ Bot	aaaGATGAAAGCTGGCTACAGGAAGGCCAGACG

Figure S11: Guide RNA composition for all experiments. Top: Image depicts the SapI sites used to ligate the gRNAs into the gRNA expression cassette. Bottom: Oligonucleotides used for all gRNAs used in this study. Lowercase nucleotides in red are the overhang sequences used to ligate into the SapI site of the gRNA expression cassette.

Supplemental Table Legends

Table S1: Dysregulated genes identified in each RNAseq experiment were subjected to ontology analysis to determine if specific classes of genes were enriched. Each RNAseq experiment is labeled in bold and underline and is referred to as either Dj or hf, referring to DjCas13d or hfCas13d; up or down referring to if the gene list was from the up or downregulated list and the name of the target gene. If genes were identified to be enriched in either the biological, cellular, or molecular ontological classifications, the classification is listed with the adjacent p-value and the genes that are part of each classification. If no classification met the adjusted p-value of > 0.05, then "nothing" is listed.

`

Table S2: Lists of genes that occur in at least 3 out of 4 RNAseq comparisons made for each comparison in Figure S8. On the left most, column is a description of what is contained in the adjacent rows: Relevant figure refers to the portion of Figure S8 the data refers to. Groups refers to which RNAseq groups the genes are co-dysregulated in. # of genes refers to the number of genes that were part of the comparison and match the associated portion of the Venn diagrams displayed in Figure S8. Genes refers to the specific genes that were found in the group. Each specific experimental comparison is highlighted in alternating dark or light gray highlight, to help differentiate the separate experimental comparisons.

Table S3: Ontology analysis for genes that were dysregulated in at least 3 out of the 4 RNAseq experiments depicted in each of the comparisons from Figure S8. The table is organized with biological and cellular ontological terms on the left. The adjusted p-value for each term in the middle and the genes that were identified in the genes lists that are part of each ontological category are listed on the right. If data in not presented for a particular comparison, it's because no genes were identified above chance or the experiment did not have any genes that occurred in the 3 out of 4 RNAseq experiments such as the case for comparisons made in Figure S8, e and f.

Table S4: Potential off-targets for each gRNA that were found to be in differentially expressed gene lists. Headings for each column describe what information the table contains.

Table S5: A list of each plasmid described in this study. Including the name of the plasmid, Addgene # if available, the ITR to ITR length of the viral genome in base pairs and which experimental figure the plasmid was used in. Note all the AAV plasmids contain viral genomes within the optimal packaging limit except for 1 which is highlighted in red. All the plasmids were created by the Ploski Lab except for the EF1a-mCherry plasmid.
