# Supplementary material for: Adeno-associated viral vector resource for the RNA-targeting Cas13d: A comparison of high-fidelity variants, DjCas13d and hfCas13d
Source: Mol Ther Methods Clin Dev. 2025 Aug 20;33(4):101565. doi: 10.1016/j.omtm.2025.101565 (PMC12466237; doi:10.1016/j.omtm.2025.101565)
Supplement: Document S2. Article plus supplemental information [file mmc7.pdf]

Molecular Therapy
Methods & Clinical Development
Original Article

Adeno-associated viral vector resource for the RNA-targeting Cas13d: A comparison of high-fidelity variants, DjCas13d and hfCas13d

Franklin Back,[1] Alfredo Sandoval,[2] Lily M. Vu,[3] Veronica M. Hong,[3] Amulya Bhaskara,[3] Sierra R. Rodriguez,[3] John T. O'Brien,[3] Benedict J. Kolber,[3] Sven Kroener,[3] and Jonathan E. Ploski[1]

[1]Department of Neuroscience and Experimental Therapeutics, Penn State College of Medicine, Hershey, PA 17033-0850, USA; [2]The University of Texas Medical Branch, Galveston, TX 77555, USA; [3]Department of Neuroscience, School of Behavioral and Brain Sciences, The University of Texas at Dallas, Richardson, TX 75080, USA

RNA-targeting CRISPR-Cas systems have emerged as alternatives to RNA-interference technology to knock down specific RNA transcripts. In particular, Cas13d derived from *Ruminococcus flavefaciens* (CasRx, RfxCas13d) has generated interest due to its superior knockdown efficiencies; however, accumulating evidence indicates that CasRx is prone to inducing transcriptome alterations due to its tendency to cleave bystander RNAs. High-fidelity Cas13d (hfCas13d) derived from CasRx and DjCas13d, an ortholog of Cas13d derived from *Ruminococcus* sp. UBA7013 (gut metagenome), are two recently identified variants that are superior to CasRx, as they both show a reduced tendency to cleave bystander RNAs. In this study, we created a resource of adeno-associated viral (AAV) vectors designed to deliver Cas13d, including hfCas13d and DjCas13d. We directly compared hfCas13d and DjCas13d for their on- and off-target potential in 293FT and neuro 2A cells. Specifically, we examined their ability to knockdown several endogenous and ectopically expressed transcripts using several different guide RNAs (gRNAs), and we examined knockdown specificity using a combination of reporter assays, RNA integrity analysis, and RNA sequencing (RNA-seq). We report that while both of these enzymes exhibit generally similar levels of knockdown potential, with DjCas13d sometimes outperforming hfCas13d, hfCas13d consistently caused significantly fewer transcriptome alterations when targeting highly expressed genes compared to DjCas13d.

INTRODUCTION

Precise and effective tools for transcriptome engineering are critical for preclinical research studies and advancing RNA-targeting therapeutics to the clinical stage. Historically, RNA interference (RNAi) technology has been a common way to manipulate RNA levels in cells. However, this technology is not without drawbacks considering it can compete with endogenous miRNA pathways and cause massive non-specific changes to the transcriptome.[1–4] An alternative technology to target RNA has emerged with the discovery of the class II CRISPR-Cas type VI, RNA-targeting Cas13 family of effectors.[5,6] These systems have been engineered for targetable RNA visualization, knockdown, base-editing, and *in vivo* isoform manipulation.[6–8]

The Cas13 family of endonucleases is characterized by a single-effector protein containing two higher eukaryotes and prokaryotes nucleotide-binding ribonuclease (RNAse) domains.[5] Similar to their DNA-targeting Cas cousins, Cas13 effectors use a guide RNA (gRNA) to target specific RNA sequences for cleavage. Unlike their DNA-targeting counterparts, they do not require protospacer adjacent motifs sequences for nucleic acid-targeting, and several Cas13 members have no discernable sequence requirement for RNA cleavage.[8] Notably, one Cas13 family member, Cas13d, gained significant attention due to its favorable qualities. Cas13d derived from *Ruminococcus flavefaciens*, commonly referred to as CasRx, was particularly exciting due to its ability to knock down RNA transcripts extremely effectively with what appeared to be little to no non-specific off-target activity.[7] Studies have indicated that it may be superior to RNAi and CRISPRi[7] for its precision and RNA knockdown efficacy. Because of these perceived benefits, CasRx was quickly adopted by the scientific community and was used to knock down RNAs in a variety of species including human cells,[7] mice,[9] plants,[10] zebrafish,[11] and several other fish species[12,13] with no apparent cytotoxicity. Furthermore, it was used successfully to alleviate disease phenotypes in several preclinical models, including Huntington's disease,[14] frontotemporal dementia,[7] acquired sensorineural hearing loss,[15] autosomal-dominant hearing loss,[9] and age-related macular degeneration.[16]

Unfortunately, several studies recently demonstrated that CasRx can cause toxicity, lethality, and substantive alterations to the transcriptome. First, it was demonstrated that CasRx caused lethality in *Drosophila melanogaster* flies when used to target RNAs in a gRNA dependent manner.[17,18] However, it was not clear at the

Received 11 December 2024; accepted 14 August 2025;
https://doi.org/10.1016/j.omtm.2025.101565.

Correspondence: Jonathan E. Ploski, PhD, Neuroscience and Experimental Therapeutics, Penn State College of Medicine, Room C3714, 500 University Drive, P.O. Box 850, MC: H109, Hershey, PA 17033-0850, USA.
E-mail: jep5929@psu.edu

© 2025 The Author(s). Published by Elsevier Inc. on behalf of The American Society of Gene and Cell Therapy. This is an open access article under the CC BY license (http://creativecommons.org/licenses/by/4.0/).

time if this was due to something unique to *Drosophila*. Then it was demonstrated that CasRx could lead to significant off-target cleavage of RNA in drosophila and human cells.[19] It was found that when CasRx engages in gRNA-dependent on-target RNA cleavage, it also engages in gRNA-independent RNA cleavage where it randomly cleaves RNAs, a phenomenon referred to as collateral cleavage. Some studies have indicated that the extent of collateral cleavage may be correlated with the degree of on-target cleavage.[20–23] This means that for highly abundant and efficiently targeted RNAs, collateral cleavage will be high when using CasRx, leading to massive changes in the transcriptome. One study also demonstrated that the expression of CasRx in mouse neurons *in vivo* led to the collateral cleavage of the 28S rRNA, resulting in death of the mice.[24]

Nevertheless, CasRx might be able to be used advantageously under certain conditions. For example, when targeting low or moderately expressed transcripts with CasRx, there may not be significant collateral cleavage activity. This explains at least in part why the original study characterizing CasRx[7] may not have detected CasRx's off-target activity since this study targeted low-expressed genes. However, it currently remains unknown what level of gene expression is too much for CasRx to effectively target without inducing significant collateral cleavage. Because of these aforementioned drawbacks of using CasRx, there has been significant interest in identifying variants of Cas13d that may exhibit a more favorable profile. One such effort led to the discovery of high-fidelity Cas13d ("hfCas13d").[20] In this study, investigators performed mutagenesis of CasRx and screened these mutants for low collateral cleavage activity and high on-target cleavage activity. Another study searched for orthologues of Cas13d that possessed an improved off-target/on-target cleavage profile and identified "DjCas13d" as a likely candidate.[21]

In the current study, we set out to produce a resource of adeno-associated viral (AAV) vectors designed to deliver Cas13d, specifically including CasRx, hfCas13d, and DjCas13d. AAV is the viral vector of choice for gene therapy due to its overall safety profile; AAV is extensively used throughout biomedical research for delivering transgenes *in vivo* and *ex vivo*. Notably, the field of neuroscience uses AAV in preclinical research models because it enables the genetic manipulation of specific regions, cells, and circuits with relative ease. We also directly compare hfCas13d and DjCas13d for their on-target efficiencies and the extent of transcriptome disturbances they may produce. Although convincing evidence exists that both hfCas13d and DjCas13d appear to be superior to CasRx for their improved specificities,[20,21] it currently remains unknown how they compare to one another. We start by sharing our findings generated with a suite of AAV vectors designed to deliver CasRx. During these initial studies, we show that CasRx engages in significant off-target cleavage activity. Our findings corroborate the findings of others that when CasRx is used to target highly expressed transcripts, it leads to significant alterations of the transcriptome,[20,21] the degradation of rRNA,[24] and depletion of the CasRx protein itself.[22] We then expanded our viral vector resource to include AAV vectors designed to deliver hfCas13d and DjCas13d. Finally, we performed extensive testing of the hfCas13d and DjCas13d vectors, comparing their ability to target ectopically expressed transcripts and endogenous transcripts in 293FT cells and neuro 2A cells, and performed extensive transcriptome analysis, using RNA sequencing (RNA-seq) to evaluate to what extent the transcriptome was altered.

RESULTS

CasRx-induced collateral activity significantly reduces highly expressed transcripts

We sought to develop a suite of AAV plasmids to deliver genes encoding CasRx and associated gRNA expression cassettes. The first set of plasmids we generated were single vector systems designed to express a gRNA expression cassette using a U6 promoter and a CasRx-mCherry gene controlled from either an elongation factor 1-alpha (EF-1α) short (EFS) promoter to drive moderate ubiquitous expression of CasRx-mCherry, a synapsin promoter to drive pan-neuronal expression, or an alpha-CaMKII promoter to drive expression in alpha-CaMKII positive cells. The mCherry coding region was separated from the CasRx coding region by a T2A ribosome skipping peptide, which should create two independent proteins from a single cistron (Figure 1A). To test these plasmids, we inserted gRNAs designed to target the mRNA coding region for blue fluorescent protein (BFP) and co-transfected them into neuro-2A cells with plasmids encoding nuclear localized BFP and imaged them for BFP and mCherry epifluorescence 48 h later (Figure 1B). The control samples, which received a non-targeting control gRNA expression cassette, all exhibited mCherry and BFP expression. In the BFP gRNA samples, BFP was robustly knocked down in the samples that received the EFS promoter version and, to a lesser extent, in samples that received the synapsin and alpha-CaMKII promotor versions of these plasmids. Overall, these data indicate that these plasmids worked as intended (Figures 1C–1E). However, we observed a significant drop in mCherry expression levels in all samples that received BFP gRNAs, but not the control gRNA samples. These data are consistent with previous findings that collateral activity induced by CasRx will decrease other highly expressed transcripts including the RNA for CasRx itself.[22] We also performed a similar experiment to test a dual vector system (e.g., CasRx and gRNA in different vectors) (Figure S1) and other single vector systems without mCherry (Figure S2). In all cases where the CasRx gene was expressed as a T2A-mCherry gene, mCherry levels dropped when the samples were treated with the BFP targeting gRNAs. In additional experiments, we verified that the BFP targeting gRNA did not directly target the mCherry RNA (Figure S3) and the depletion of CasRx protein that we observed was not unique to the BFP RNA, but occurred when other gRNAs were used to target other highly expressed RNAs (Figures S4A and S4B). Notably, several studies have been published that indicate that when CasRx is directed to target a highly expressed transcript, CasRx protein levels become depleted presumably because CasRx mediated collateral cleavage cleaves its own transcript.[22] We did, however, examine if the loss of CasRx protein might be due in part to the actions of the ubiquitin-proteasome system, but found no evidence that this contributes significantly (Figures S4C–S4G).

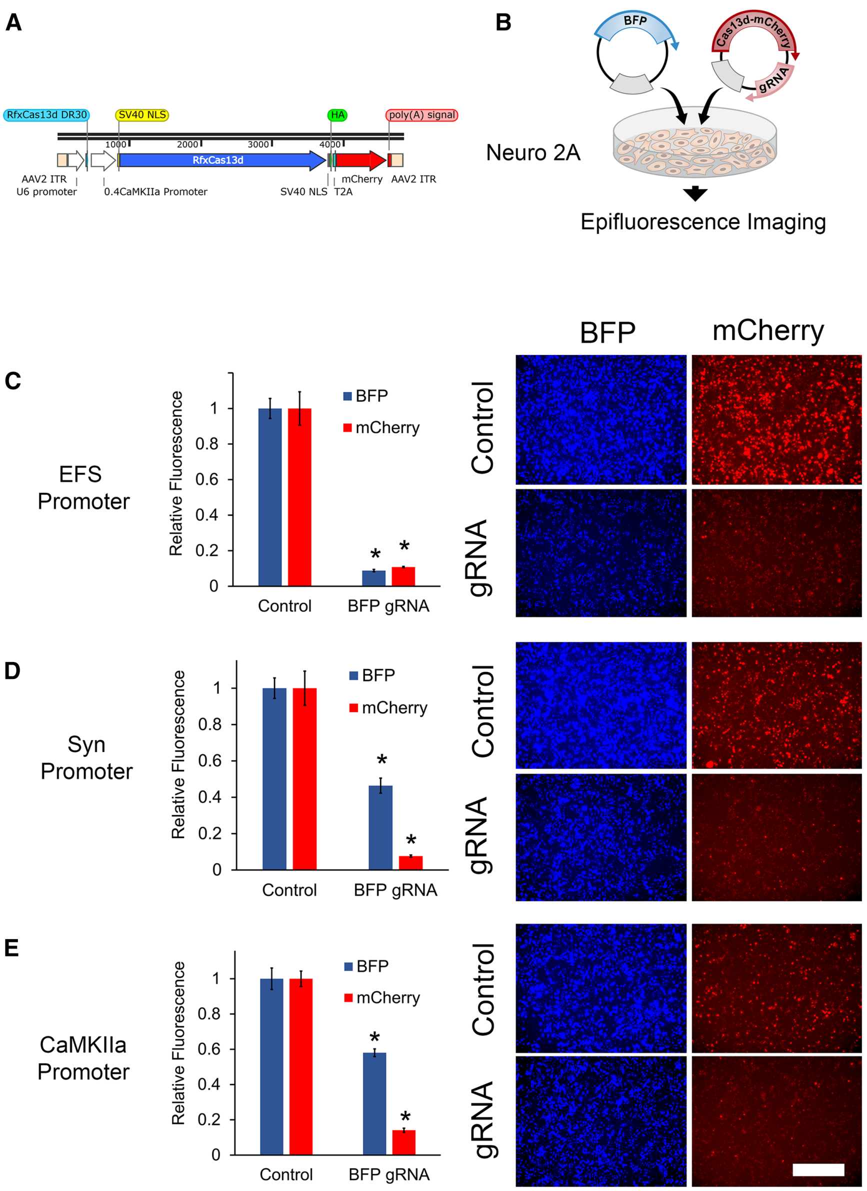

Figure 1. Demonstration of the functionality of AAV-based gRNA/CasRx (single vector) plasmids in neuro-2A cells

(A) Schematic of AAV vector map designed to express a gRNA from a U6 promoter and a CasRx(RfxCas13d)-mCherry gene controlled from an alpha-CaMKII promoter. Additional vectors were similarly designed to include an EF-1α short (EFS) or synapsin promoter, instead of the alpha-CaMKII promoter to control CasRx expression (not shown). (B) Graphic describing the outline of the experiment. (C–E) Single vector plasmids containing a BFP targeting gRNA or non-targeting gRNA control plasmids were co-transfected into neuro 2A cells with plasmids designed to express nuclear-localized BFP, and 48 h later, the cells were imaged for BFP and mCherry epifluorescence. EFS, synapsin, and alpha-CaMKII versions shown respectively in (C), (D), and (E). Quantification of data depicted on left side; representative images presented on right side. BFP gRNAs treated samples all exhibited a significant reduction in BFP levels compared to controls. Surprisingly mCherry levels also dropped in all BFP gRNA treated conditions. Error bars = standard error of the mean. Four biological replicates (n = 4) were used for all experimental groups. Asterisk (*) indicates significant difference compared to respective control group (t test, two-tailed, $^*p < 0.003$). Scale bars, 400 microns.

CasRx-mediated RNA targeting leads to significant transcriptome changes

To examine CasRx-mediated changes at the RNA level, we purified RNA from the vehicle-treated samples described in Figures S4C and S4D. We examined the RNA on an Agilent Bioanalyzer and observed two distinct bands below the 28S rRNA band only in RNA samples that were treated with BFP gRNAs (Figures 2A and 2B). These bands are likely due to CasRx-mediated cleavage of the 28S rRNA.[24] Quantitative PCR revealed that BFP RNA levels were knocked down approximately 4-fold compared to control samples (Figure 2C). Next, we subjected these RNA samples to whole transcriptome analysis using RNA-seq. We identified hundreds of genes to be up- and downregulated in these samples. In this case where CasRx was targeting a highly expressed transcript, it led to a significant distortion of the transcriptome (Figures 2D and 2F). The RNA-seq analysis also corroborated our qPCR BFP knockdown data (Figure 2E).

Vectors for delivery of high-fidelity variants of Cas13d knock down ectopically expressed genes efficiently

Considering our findings and those of others[20,21] that CasRx can lead to significant distortion of the transcriptome under certain conditions, we next sought to develop AAV vectors that could be used to deliver the recently identified high-fidelity variants of Cas13d: hfCas13d and DjCas13d. To this end, we converted our existing EFS-CasRx-T2A-mCherry AAV plasmid to a hfCas13d plasmid by mutating 4 alanine codons in CasRx so they instead code for valines.[20] We also developed a similar vector with human codon-optimized DjCas13d (Figure 3) and other vectors with P2A sites or without 2A sites (Figure S5). To directly compare Cas13d proteins for their ability to knock down intended targets and to assess to what extent non-specific activity occurred, we transfected these vectors into 293FT cells with a GFP-*brain-derived neurotrophic factor (BDNF)* encoding plasmid and two different gRNA expression cassettes designed to target *BDNF* (BDNF 1 and 2), or

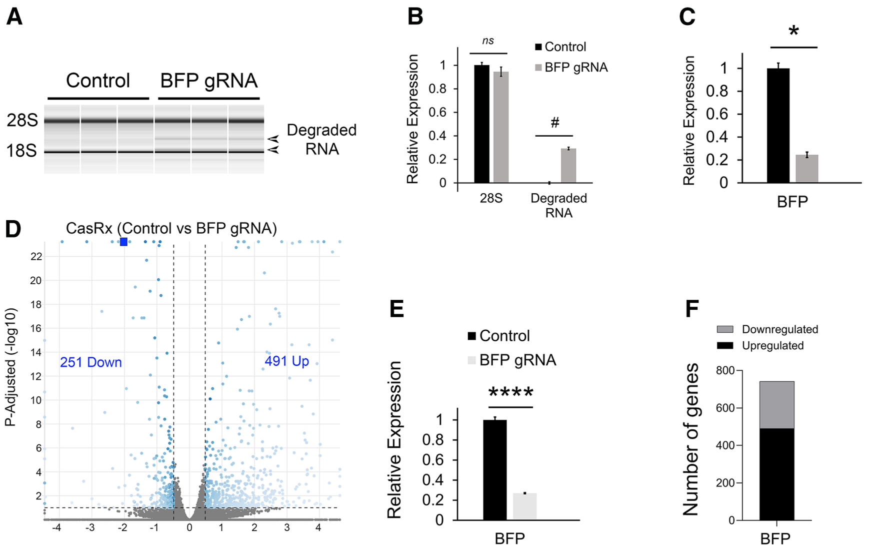

Figure 2. CasRx-mediated RNA targeting leads to significant transcriptome changes

(A) RNA from the vehicle-treated samples is described in Figures S4C and S4D were examined on an Agilent Bioanalyzer, and two distinct bands below the 28S rRNA band were detected only in RNA samples that were treated with BFP gRNAs but not control samples indicating the presence of degraded RNA. Representative images are shown for a group size of 3. Arrows point to the two degraded RNA bands. The location of the 28S and 18S rRNA bands indicated. (B) Quantification of these bands revealed a significant presence of degraded RNA in the BFP gRNA groups compared to controls, (Mann-Whitney U test, #$p < 0.05$). A significant difference in the 28S was not detected (t test, *ns* = $p > 0.05$). Five biological replicates for all groups ($n = 5$). (C) Quantitative PCR for BFP mRNA was performed on the samples and BFP RNA levels were significantly knocked down approximately 4-fold (t test, two-tailed, *$p = 5.02E-07$). Five biological replicates for all groups ($n = 5$). (D) These RNA samples were subjected to whole transcriptome analysis using RNA-seq. A volcano plot depicts the differential expression observed between samples treated with the BFP gRNA compared to controls. Gene lists were determined by filtering the data to only include genes with an adjusted p value of 0.1 and a fold change of ±1.4-fold. Blue dots indicate differentially expressed genes. Gray dots indicate genes that are not differentially expressed. The horizontal dashed line indicates the threshold for significance and the vertical dashed lines indicate the ±1.4-fold cut-off. BFP is indicated by the blue square. We identified 251 genes to be downregulated and 491 genes to be upregulated in these samples treated with the BFP gRNA compared to controls. Three biological replicates for all groups ($n = 3$). (E) RNA-seq data was graphed to depict the relative change in BFP RNA levels between the groups. **** indicates adjusted p value, $p < 9.14E-18$. (F) The number of genes up and downregulated in this RNA-seq experiment are graphed. Error bars = standard error of the mean.

a gRNA expression cassette designed to target LacZ as a control (Figures 3C–3G). We also conducted a similar experiment testing two gRNAs, (Calcrl 1 and 2) designed to target GFP-*Calcrl* (Figures 3H and 3I). Twenty-four hours later, the cells were imaged for GFP and mCherry (RFP) epifluorescence (Figures 3C–3E and 3H–3J) and the RNA from these cells was subsequently examined via an Agilent Bioanalyzer (Figures 3F–3G, 3K, and 3L). CasRx produced robust knockdown of both *BDNF* and *Calcrl* GFP and CasRx-mCherry for all *BDNF* and *Calcrl* targeting gRNAs compared to LacZ controls (Figures 3C–3E and 3H–3J). In these experiments, hfCas13d and DjCas13d produced in some cases slightly less *BDNF* and *Calcrl* knockdown compared to CasRx. The *BDNF* gRNA groups did not exhibit a reduction in mCherry expression compared to LacZ controls in the hfCas13d treatment group. In contrast, treatment with these same gRNAs when using DjCas13d led to a significant reduction of mCherry expression compared to LacZ controls suggesting off-target collateral cleavage. Similarly, the *Calcrl* gRNA groups when treated with hfCas13d resulted in significantly less reduction of mCherry compared to CasRx and DjCas13d. These findings indicate that under these circumstances, hfCas13d induced less collateral cleavage compared to CasRx and DjCas13d. RNA examined via Agilent Bioanalyzer revealed RNA degradation in the CasRx, *BDNF*, and *Calcrl* gRNA treatment groups and to a lesser extent in the *Calcrl* 1 hfCas13d group (Figures 3F, 3G, 3K, and 3L). In a similar experiment, we also imaged cells at 48 h post-transfection and found generally similar results (Figures S6 and S7).

Whole transcriptome analyses indicates that the use of DjCas13d led to more transcriptome alterations compared to hfCas13d when targeting highly expressed RNAs

We sought to compare hfCas13d and DjCas13d for their abilities to knock down a specific target and to assess their tendencies for non-specific transcriptome alterations by performing whole transcriptome analysis using RNA-seq. Specifically, we transfected 293FT cells with hfCas13d and DjCas13d-encoding plasmids along with plasmids encoding GFP-*BDNF* and BDNF 1 or LacZ gRNAs, or GFP-*Calcrl* and *Calcrl* 1 or LacZ gRNAs and harvested the cells 48 h post-transfection for RNA-seq analysis (Figures S6 and 4A–4G). Both hfCas13d and DjCas13d were capable of robust knockdown of GFP-*Calcrl* and GFP-*BDNF*, but DjCas13d was slightly better at knocking down its target RNA when targeting GFP-*BDNF* (Figure 4C). However, in these experiments, the use of DjCas13d led to many more genes being up and down regulated compared to hfCas13d (Figures 4A, 4B, 4D, 4E, and 4G).

We repeated the transfections, and 48 h later, the cells were homogenized for protein quantification by western blotting. Both hfCas13d and DjCas13d efficiently reduced the expression levels of their intended targets GFP-*BDNF* (Figures 4H and 4J) and GFP-*Calcrl* (Figures 4K and 4M) with no detectable differences in efficacy. Notably, the Cas13d protein levels were reduced in both high-fidelity variants when the active gRNA was present, indicating collateral cleavage. Moreover, DjCas13d showed lower expression levels when compared to hfCas13d in the LacZ conditions (Figures 4H,

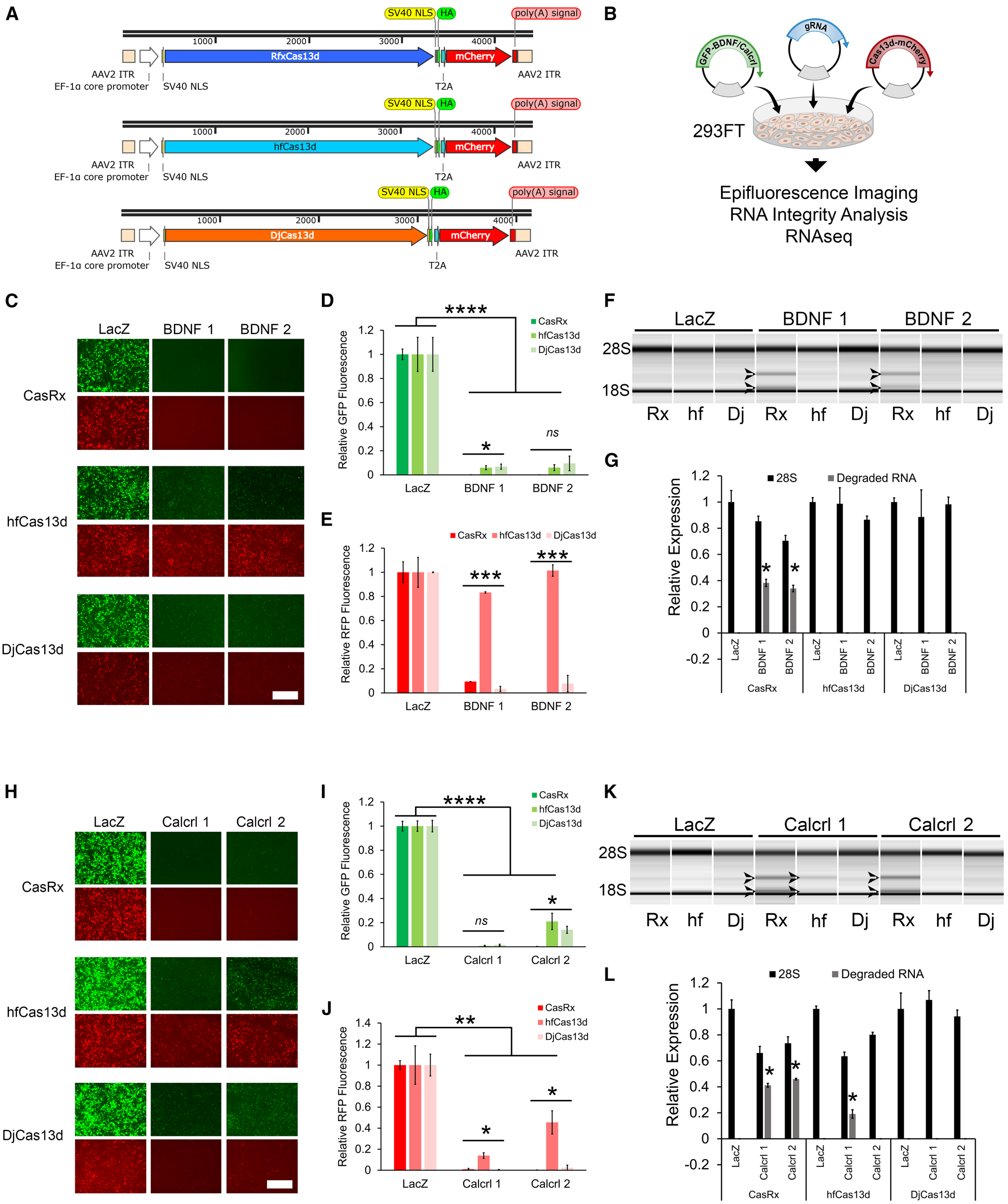

Figure 3. High-fidelity variants of Cas13d knock down ectopically expressed genes efficiently

(A). Schematic of AAV vector maps depicting AAVs designed to express CasRx(RfxCas13d)-mCherry, hfCas13d-mCherry, or DjCas13d-mCherry from an EFS promoter, (EF-1a core promoter). (B) Graphic describing the outline of the experiment. (C) 293FT cells were transfected with either of these Cas13d encoding vectors, a GFP-*BDNF*

(legend continued on next page)

4I, 4K, and 4L), despite transfections being performed with the same amount of Cas13d encoding plasmid DNA.

Ectopic expression of transgenes in transfected cells can lead to super-physiological RNA/protein levels of the transgene gene-product. For instance, ectopic expression of GFP-*Calcrl* and GFP-*BDNF* both resulted in very high levels of their respective RNAs (~10,000 transcripts per million [TPM]) (data not shown). In contrast, the next highest expressed nuclear-encoded gene was GAPDH at ~6,000 TPM. For our purposes, this is ideal because we wanted to directly test hfCas13d and DjCas13d targeting very highly expressed RNAs. However, in our next experiment, we sought to compare hfCas13d and DjCas13d for their on- and off-targeting potential of two endogenously expressed transcripts, *actin gamma 1*, (*ACTG1*) and *peptidylprolyl isomerase A*, (PPIA). In 293FT cells, *ACTG1* (~1,200 TPM) and *PPIA* (~160 TPM) are both highly expressed RNAs. Notably, these RNAs are among the top 5% of mostly highly expressed RNAs in 293FT cells according to our RNA-seq data (see materials and methods). We transfected 293FT cells with hfCas13d and DjCas13d encoding plasmids along with plasmids encoding *ACTG1* gRNA, *PPIA* gRNA, or control gRNAs and harvested the cells 48 h post-transfection for RNA-seq analysis (Figure 5). Both hfCas13d and DjCas13d led to a significant knockdown of *ACTG1* with knockdown between the two not differing significantly (Figures 5A–5C); however, the use of DjCas13d induced a significant up- and downregulation of hundreds of genes mirroring the non-specific issues with the original CasRx (Figures 5B and 5G). In contrast, hfCas13d caused a significant dysregulation of many fewer genes (Figures 5A and 5G). Treatment with DjCas13d led to a significant, although very modest knockdown of *PPIA*, but it still resulted in a significant dysregulation of several hundred genes (Figures 5E and 5G). The use of hfCas13d induced a significant knockdown of *PPIA* with very few significantly dysregulated genes and knocked down PPIA slightly better than DjCas13d (Figures 5D–5G).

We repeated the transfections targeting endogenously expressed gene products, and 48 h later, the cells were homogenized for protein quantification by western blotting. When targeting *ACTG1*, neither hfCas13d nor DjCas13d showed significant Cas13d protein reduction, (Figures 5I–5L). No changes in Cas13d protein levels were detected when *PPIA* was targeted by hfCas13d (Figures 5M and5N). However, DjCas13d protein levels were reduced when the active gRNA was present, indicating collateral cleavage (Figures 5M and 5O). Once more, the hfCas13d and DjCas13d protein levels from the control groups differ despite transfections being performed with the same amount of Cas13d encoding plasmid DNA (Figure 5P).

In our next set of experiments, we developed another set of Cas13d encoding AAV genome plasmids that contain the immediate/early promoter enhancer of cytomegalovirus (CMV promoter) and a chimeric intron to drive robust expression of Cas13d (Figure 6A). Due to the space limitations of the viral genome, we did not include an mCherry encoding sequence in sequence with the Cas13d like we had in the previous set of vectors. We did, however, include an mCherry encoding gene within the plasmid, but it was inserted outside of the viral genome region to serve as marker for plasmid transfection and non-specific collateral cleavage. We co-transfected these plasmids into mouse neuro-2A cells along with a GFP-*Calcrl* encoding plasmid and either a gRNA expression cassette designed to target *Calcrl* (Calcrl 2), or a gRNA expression cassette designed to target LacZ as a control (Figure 6B). Forty-eight hours

encoding plasmid and two different gRNA expression cassettes, *BDNF* 1 and 2, or a gRNA expression cassette designed to target LacZ as a control. Twenty-four hours post-transfection, the cells were imaged for GFP and mCherry (RFP) epifluorescence. Representative images are shown. (D) Quantification of data revealed that treatment with both *BDNF* gRNAs resulted in robust knockdown of GFP-*BDNF* for each of the Cas13d variants compared to LacZ controls (**** = ANOVA, $p < 0.00005$; Fisher's LSD, $p < 0.00005$). There was a significant difference in GFP-*BDNF* knockdown for *BDNF* 1 among the Cas13d variants, with CasRx outperforming the other Cas13d variants (* = ANOVA, $p = 0.03$; Fisher's LSD, $p < 0.03$). There was not a significant difference in GFP-BDNF knockdown for BDNF 2 among the Cas13d variants, (*ns* = ANOVA, $p = 0.258$). Four biological replicates for all groups ($n = 4$). (E) Both DjCas13d and CasRx groups, for both *BDNF* 1 and 2, exhibited a significant reduction in RFP levels compared to LacZ controls, (ANOVA, $p < 0.000002$; Fisher's LSD, $p < 0.000002$); however, RFP levels were not significantly different from LacZ controls for hfCas13d (ANOVA, $p = 0.23$). RFP levels for the hfCas13d, *BDNF* 1 and 2 groups were significantly higher than the DjCas13d and CasRx, *BDNF* 1 and 2 groups (*** = ANOVA, $p < 2.7E-09$; Fisher's LSD, $p < 1.82E-09$). Four biological replicates for all groups ($n = 4$). (F) RNA was purified from samples described in (C and D), and examined on an Agilent Bioanalyzer. The CasRx, *BDNF* 1 and 2 groups exhibited 2 RNA bands likely a result of RNA degradation, which were not found in the LacZ control groups nor any of the samples treated with DjCas13d or hfCas13d. Representative images are shown. Arrows point to the two degraded RNA bands. The location of the 28S and 18S rRNA bands indicated. Four biological replicates for all groups ($n = 4$). (G) Quantification of bioanalyzer data presented. (Mann-Whitney U test, *$p < 0.05$). (H) 293FT cells were transfected with either of these Cas13d encoding vectors, a GFP-*Calcrl* encoding plasmid and two different gRNA expression cassettes, *Calcrl* 1 and 2, or a gRNA expression cassette designed to target LacZ as a control. Twenty-four hours post-transfection, the cells were imaged for GFP and mCherry (RFP) epifluorescence. Representative images are shown. (I) Quantification of images revealed that treatment with both *Calcrl* gRNAs resulted in robust knockdown of GFP-*Calcrl* for each of the Cas13d variants compared to LacZ controls (**** = ANOVA, $p < 2.54E-07$; Fisher's LSD, $p < 7.69E-07$). There was not a significant difference in GFP-*Calcrl* knockdown for *Calcrl* 1 among the Cas13d variants, (*ns* = ANOVA, $p = 0.19$). There was a significant difference in GFP-*Calcrl* knockdown for *Calcrl* 2 among the Cas13d variants, with CasRx outperforming the other Cas13d variants (* = ANOVA, $p = 0.021$; Fisher's LSD, $p < 0.05$). Four biological replicates for all groups ($n = 4$). (J) Each of the Cas13d variants, for both *Calcrl* 1 and 2, exhibited a significant reduction in RFP levels compared to LacZ controls, (** = ANOVA, $p < 0.011$; Fisher's LSD, $p < 0.013$). RFP levels for the hfCas13d, *Calcrl* 1, and 2 groups were significantly higher than the DjCas13d and CasRx, *Calcrl* 1 and 2 groups (* = ANOVA, $p < 0.0011$; Fisher's LSD, $p < 0.00101$). Four biological replicates for all groups ($n = 4$). (K) RNA was purified from samples described in (G–I), and examined on an Agilent Bioanalyzer. The CasRx, *Calcrl* 1, and 2 groups and the hfCas13d, *Calcrl* 1 group exhibited 2 RNA bands likely a result of RNA degradation, which was not found in the LacZ control groups nor any of the other samples. Representative images are shown. Arrows point to the two degraded RNA bands. The location of the 28S and 18S rRNA bands indicated. Four biological replicates for all groups ($n = 4$). (L) Quantification of bioanalyzer data presented. (Mann-Whitney U test, *$p < 0.05$). Error bars = standard error of the mean. Scale bars, 450 microns.

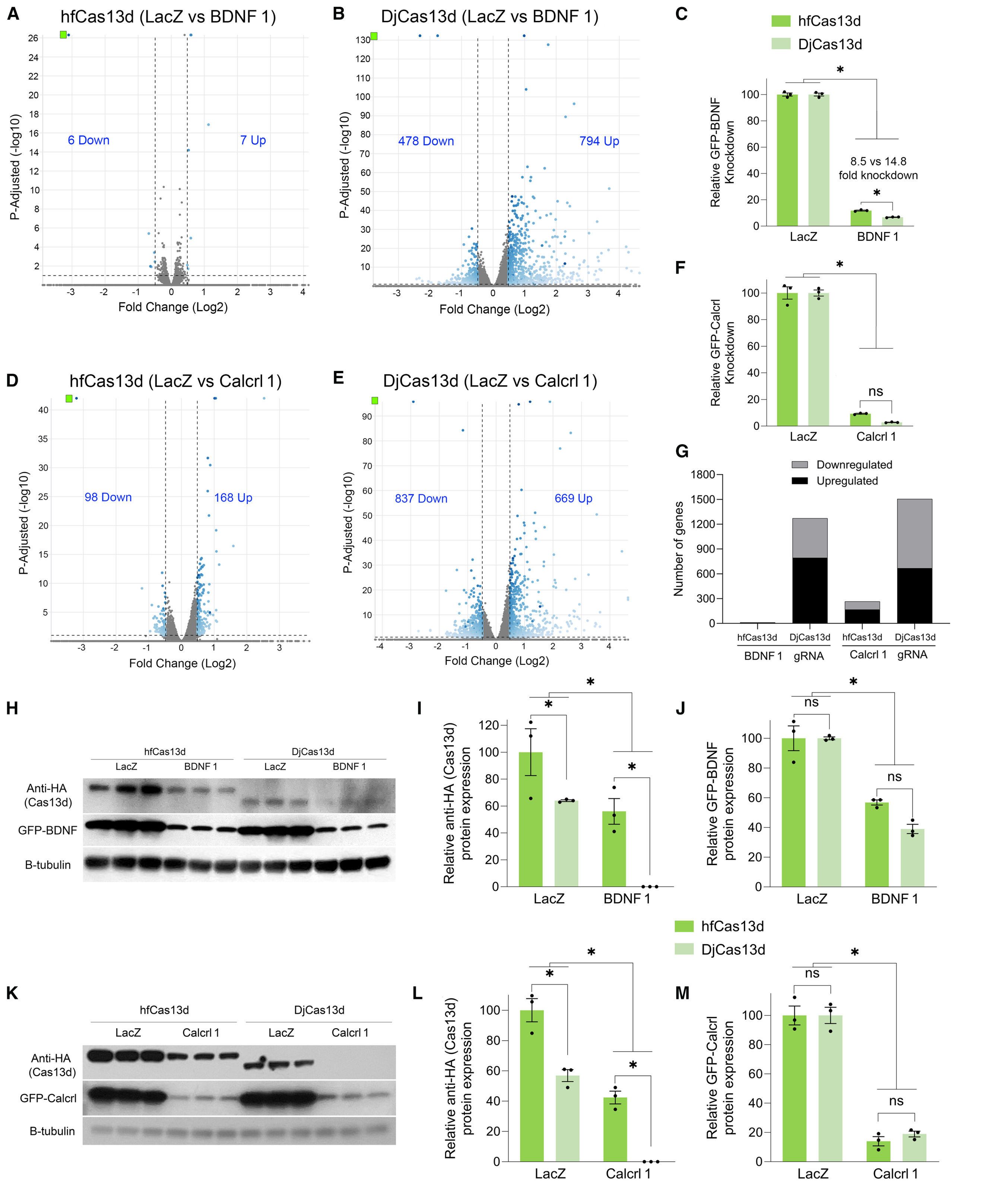

(legend on next page)

post-transfection, the cells were imaged for epifluorescence for GFP and mCherry. Imaging revealed that CasRx, hfCas13d, and DjCas13d all were capable of reducing GFP-*Calcrl* levels (Figure 6C). Monomeric Cherry levels were also reduced in the CasRx and DjCas13d treated samples compared to controls; however, hfCas13d treated samples did not exhibit this reduction in mCherry signal (Figure 6C), which is consistent with reduced collateral cleavage, similar to the results presented previously. RNA analysis via Agilent Bioanalyzer revealed no obvious evidence of rRNA degradation in any of the hfCas13d or DjCas13d treated samples (data not shown). Transcriptome analysis via RNA-seq demonstrated that once again the use of DjCas13d to knockdown GFP-*Calcrl* led to significant transcriptome distortion resulting in hundreds of genes being up- and downregulated compared to the LacZ gRNA-treated control (Figures 6D and 6E). It is noteworthy that the use of hfCas13d in this experiment did not cause any detectable transcriptome dysregulation other than knocking down the intended target GFP-*Calcrl* (Figure 6D). Both hfCas13d and DjCas13d were capable of knocking down GFP-*Calcrl* efficiently and there was not a significant difference between the two (Figure 6F); however, only DjCas13d significantly knocked down mCherry consistent with it causing collateral RNA cleavage (Figure 6G).

We repeated the transfections into neuro-2A cells, and 48 h later, the cells were homogenized for protein quantification by western blotting. The GFP-*Calcrl* protein levels were reduced by both hfCas13d and DjCas13d, but knockdown efficiency was superior with the hfCas13d variant (Figures 6H and 6J). In parallel with the epifluorescence results, a reduction in mCherry protein indicated collateral cleavage from DjCas13d when actively knocking down its target (Figures 6H and 6K). The Cas13d protein levels remained unaltered between the control and *Calcrl* 2 gRNA-treated samples (Figures 6H and 6I); however, control levels of DjCas13d were once again lower than hfCas13d control levels. (Figures 6H and 6I).

When making comparisons between hfCas13d and DjCas13d, it is necessary to examine their relative protein levels so that we can rule out that any differences in efficacy or transcriptome dysregulation between the two are not due to differences in their protein levels. We found that DjCas13d protein levels were significantly lower than hfCas13d protein levels, even in non-targeting control conditions (data shown in Figures 4, 5, and 6). Notably, the transgene structure of hfCas13d and DjCas13d was identical, so expression differences had to be caused by the coding regions of these two transgenes. DjCas13d was codon optimized, too, so this should not be the explanation for the differences between the two. Regardless, DjCas13d consistently exhibited much lower protein levels across these experiments, and despite its lower levels, it caused far more collateral cleavage. Therefore, the higher levels of transcriptome dysregulation caused by DjCas13d compared to hfCas13d cannot be explained by its protein levels, because its protein levels were lower than hfCas13d′s. However, because DjCas13d protein levels were lower, it remains possible that it may exhibit greater knockdown efficacy compared to hfCas13d when their protein levels are normalized. To assess this possibility, we adjusted the amount of hfCas13d encoding plasmid we transfected into the cells, so we could normalize hfCas13d protein levels to DjCas13d protein levels and reassessed gene product knockdown via qPCR. To achieve this goal, we transfected 293FT cells with a range of hfCas13d encoding plasmid DNA amounts (1, 1/2, 1/4, or 1/8), while keeping the amount of DjCas13d encoding plasmid DNA amounts constant, and then compared their protein expression levels (Figure 7A). We determined that to create similar levels of hfCas13d and DjCas13d protein levels we needed to transfect the cells with 1/3.5 of the hfCas13d encoding plasmid compared to the DjCas13d encoding plasmid so that the protein levels would be expressed at the same level (Figures 7A and 7B). Once we established the correct plasmid ratio of hfCas13d to DjCas13d to create similar protein levels, we repeated the knockdown experiments and performed qPCR for GFP-*BDNF* (Figure 7C), GFP-*Calcrl* (Figure 7D), *ACTG1* (Figure 7E), and

Figure 4. Whole transcriptome analysis of potential on and off-targeting of two ectopically expressed transcripts using hfCas13d and DjCas13d

(A) To directly compare hfCas13d and DjCas13d for their on and off-targeting potential, 293FT cells were transfected with hfCas13d or DjCas13d encoding vectors, and either a GFP-*BDNF* encoding plasmid and *BDNF* 1 or LacZ gRNA expression cassettes, or GFP-*Calcrl* and *Calcrl* 1 or LacZ gRNA expression cassettes (same samples shown in S6). Forty-eight hours post-transfection, the RNA was isolated and subjected to whole transcriptome analysis using RNA-seq. Volcano plots depict the differential expression observed between samples treated with *BDNF* 1 gRNAs compared to LacZ gRNA controls (A and B). Gene lists were determined by filtering the data only to include genes with an adjusted p value of 0.1 and a fold change of ±1.4-fold. Blue dots indicate differentially expressed genes. Gray dots indicate genes that are not differentially expressed. The horizontal dashed line indicates the threshold for significance and the vertical dashed lines indicate the ±1.4-fold cut-off. The green square indicates the target GFP-*BDNF*. The number of genes up and downregulated are indicated on each volcano plot. GFP-*BDNF* is significantly and robustly knocked down in both hfCas13d and DjCas13d conditions, but the use of DjCas13d leads to many more transcripts being up and downregulated compared to similar experiments using hfCas13d. (C) RNA-seq data was graphed to depict the relative changes in GFP-*BDNF* RNA levels compared to LacZ controls. Both DjCas13d and hfCas13d significantly knocked down their intended targets in this experiment, but DjCas13d knocked down its target GFP-*BDNF* significantly more than hfCas13d. When targeting GFP-*Calcrl*, shown in green squares in (D and E), a similar result was observed, with DjCas13d presenting a higher number of differentially expressed genes, but there was no significant difference in knockdown of GFP-*Calcrl* between hfCas13d and DjCas13d (F). (G) A comparison of the number of up and downregulated genes in these RNA-seq experiments, indicating lower transcriptome changes for hfCas13d across experiments. To directly compare protein expression of hfCas13d and DjCas13d and target protein knockdown, the experiment was repeated, but this time the cells were harvested for western blot analysis. The levels of both hfCas13d and DjCas13d were reduced when the active gRNAs were present, indicating collateral cleavage (H and I), and both Cas13ds reduced the expression of GFP-*BDNF* (H and J); when targeting GFP-*Calcrl*, both hfCas13d and DjCas13d show a reduction of the Cas13d protein (K and L) and the targeted protein (K and M). Notably, DjCas13d showed lower expression levels when compared to hfCas13d in the LacZ conditions (I and L). Two-way ANOVAs (*$p < 0.05$), error bars = standard error of the mean. Three biological replicates for all groups ($n = 3$). β-tubulin, protein loading control.

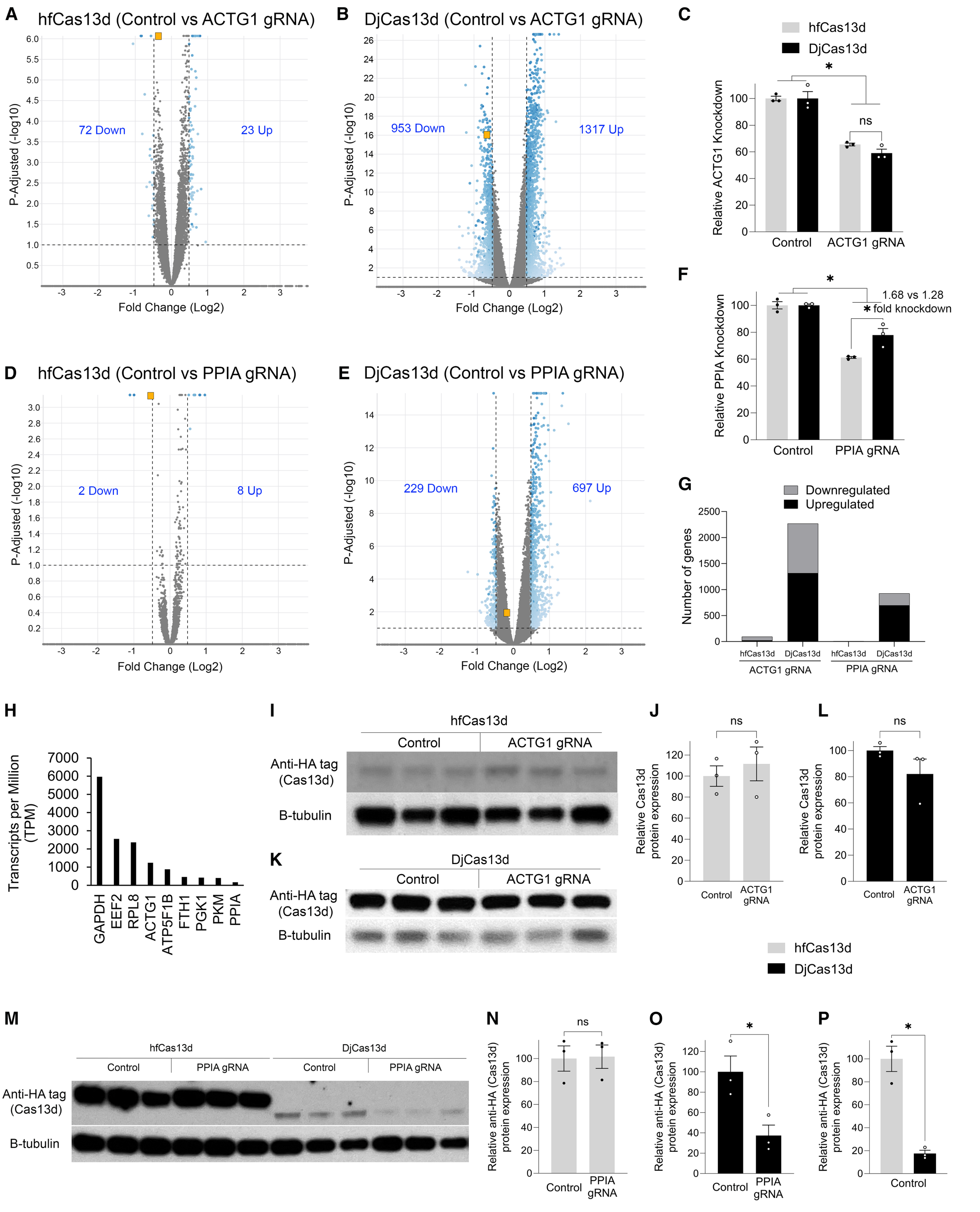

(legend on next page)

(legend on next page)

PPIA (Figure 7F). Three out of the 4 knockdown experiments showed that hfCas13d and DjCas13d did not differ in their ability to knockdown their target transcript. Only in the experiment targeting *ACTG1*, did DjCas13d outperform hfCas13d and deplete *ACTG1* RNA levels more.

Transcriptome changes are likely not due to gRNA off-targeting

The RNA-seq experiments revealed that many genes were dysregulated following the use of DjCas13d and to a much lesser extent, the use of hfCas13d. Ontology analysis was performed for the genes in each gene list to determine if any classes of genes were enriched in each experiment and the enriched genes are listed in Table S1. Next, we sought to determine if there were specific genes that are consistently dysregulated across experiments. Therefore, we cross-referenced the gene lists across these RNA-seq experiments to determine if there were similarities in the genes dysregulated. Any genes dysregulated consistently could indicate that DjCas13d or hfCas13d may preferentially degrade specific RNAs or induce expression of specific classes of RNA. These data are displayed as Venn diagrams (Figure S8). We compared the genes downregulated or upregulated in the experiments where we sought to knockdown *BDNF* and *Calcrl* (Figures S8A and S8B) or *ACTG1* and *PPIA* (Figures S8C and S8D), using DjCas13d and hfCas13d. The two genes that were consistently downregulated for the *BDNF*/*Calcrl* experiments were GFP as expected and *PPFIA4* (Figure S8A). The two genes consistently upregulated were histone protein, *H2B clustered histone 12* (*H2BC12*), and *small nucleolar RNA host gene 15* (*SNHG15*) (Figure S8B). For the *ACTG1*/*PPIA* experiments, no genes were consistently downregulated and arrestin domain containing 3 gene was the only gene consistently upregulated. We then compared genes dysregulated only by hfCas13d (Figures S8E and S8F) or by DjCas13d (Figures S8G and S8H) and sought to determine if there were classes of genes that were dysregulated in at least 3 out of the 4 RNA-seq experiments. For the hfCas13d comparison, no genes were found to be dysregulated in at least 3 of the 4 experiments (Figures S8E and S8F). For the DjCas13d comparison, several genes were found to be dysregulated in at least 3 of the 4 experiments and these genes were subjected to ontology analysis. This analysis revealed that genes associated with the extracellular matrix were enriched in these downregulated gene lists, and genes associated with RNA and ribosomal processing and regulation were enriched in these upregulated gene lists. However, the number of enriched genes were relatively few. The genes identified to be dysregulated in at least 3 of the 4 experiments, for each Venn diagram displayed in Figure S8 are listed in Table S2. The genes listed for each experimental comparison in Table S2 were subjected to ontology analysis, and genes classes that were identified above chance are listed in Table S3.

We also sought to determine if the genes that were found to be dysregulated in these RNA-seq experiments were a product of off-target gRNA binding. To accomplish this, we searched the human genome for genes that possessed similarity to the gRNAs used in this study and then cross-referenced them with the genes that were dysregulated in the RNA-seq experiments. In general, very few genes in the dysregulated gene list showed any similarity to the gRNA sequences. In the cases of genes that did show similarity, the degree of similarity was likely too low to be a legitimate gRNA target (Table S4). Therefore, the transcriptome changes detected are unlikely to be attributed to off-target gRNA binding.

AAVs encoding Cas13d can knock down targets *in vitro* and *in vivo*

The EFS-Cas13d-mCherry (Figure 3A) and the CMV-intron-Cas13d (Figure 6A) encoding AAVs were used to transduce 293FT cells in culture. Seventy-two hours later, the cells were harvested and protein lysates from these cells were examined for Cas13d protein levels via western blotting with an anti-hemagglutinin (HA) tag antibody (Figure 8A). Only the cells that received the CMV-intron encoding Cas13d AAVs exhibited detectable expression of the HA-tagged Cas13d protein (Figure 8B). In addition, we did not observe mCherry epifluorescence from the cells that received the EFS-Cas13d-mCherry AAVs either (data not shown). The EFS-Cas13d-mCherry plasmids

Figure 5. Whole transcriptome analysis of potential on and off-targeting of two endogenously expressed transcripts using hfCas13d and DjCas13d
(A) To directly compare hfCas13d and DjCas13d for their on and off-targeting potential, 293FT cells were transfected with hfCas13d and DjCas13d encoding vectors, and either with gRNAs targeting *ACTG1* or *PPIA* or control gRNAs. Forty-eight hours post-transfection, the RNA was isolated and subjected to whole transcriptome analysis using RNA-seq. Volcano plots depict the differential expression observed between samples treated with *ACTG1* gRNAs compared to control gRNA controls (A and B). Gene lists were determined by filtering the data only to include genes with an adjusted p value of 0.1 and a fold change of ±1.4-fold. Blue dots indicate differentially expressed genes. Gray dots indicate genes that are not differentially expressed. The horizontal dashed line indicates the threshold for significance and the vertical dashed lines indicate the ±1.4-fold cut-off. The orange squares represent the target *ACTG1* gene. The number of genes up and downregulated are indicated on each volcano plot. The use of both hfCas13d and DjCas13d leads to the knockdown of *ACTG1*; however, the use of DjCas13d leads to many more transcripts being up and downregulated compared to hfCas13d. (C) RNA-seq data was graphed to depict the relative changes in *ACTG1* RNA levels compared to controls. DjCas13d or hfCas13d did not significantly differ in their ability to knockdown *ACTG1*. When targeting *PPIA*, shown in orange squares in (D and E), both hfCas13d and DjCas13d knocked down *PPIA* RNA levels. However, hfCas13d led to significantly better knockdown of *PPIA* compared to DjCas13d (F). (G) Shows a comparison of the number of up and downregulated genes, indicating lower transcriptome changes for hfCas13d across experiments. (H) Depicts the relative expression levels in transcripts per million (TPM), in 293FT cells for selected genes including *ACTG1* and *PPIA*, showing that both are highly expressed genes (data derived from the RNA-seq experiment). To directly compare protein expression of hfCas13d and DjCas13d, the experiment above was repeated but cells were harvested for western blot analysis. The levels of both hfCas13d (I and J) and DjCas13d (K and L) proteins remained unchanged between control and *ACTG1* gRNA groups. When targeting *PPIA*, hfCas13d protein levels remained unchanged (M and N), while DjCas13d levels were reduced (M and O), indicating collateral cleavage. hfCas13d and DjCas13d protein levels differ significantly in the control groups (M and P). Two-way ANOVAs, $^*p < 0.05$ were performed on data presented in (C and F); independent t tests, $^*p < 0.05$ were performed on data presented in (J, L, and N–P). Error bars = standard error of the mean. Three biological replicates for all groups ($n = 3$). B-tubulin, protein loading control.

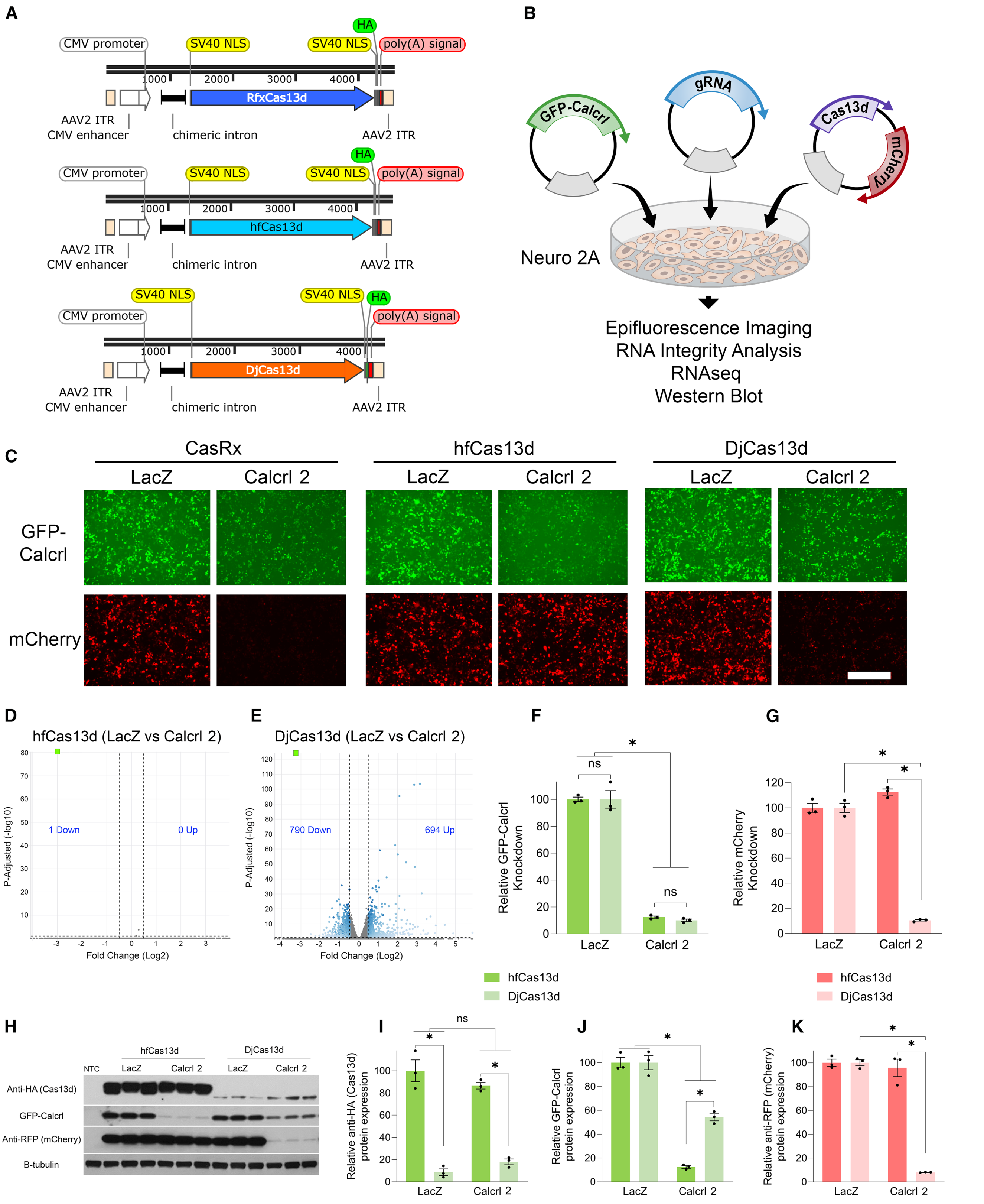

(legend on next page)

clearly function as intended as demonstrated in Figures 3, 4, 6, S1, S5, and S6. The fact that we do not see expression of Cas13d-mCherry here is most likely attributed to the much lower transgene copy number delivered to cells when transducing cells in culture with AAV versus plasmid transfection. These results are also consistent with the fact that the CMV-intron leads to a much stronger expression than the EFS promoter. The EFS promoter is small (~250 bps), which can be advantageous when space is limited in the AAV genome, but it also comes at a cost because of its reduced ability to drive expression. We recommend the end user to take these findings into consideration when choosing which AAV plasmid best suits their experimental needs.

Next, we transduced 293FT cells with the CMV-intron encoding Cas13d AAVs, an AAV encoding a GFP gene, and AAVs either encoding a gRNA expression cassette designed to knock down GFP or LacZ as a control. Seventy-two hours later, the cells were harvested and qPCR was performed to assess GFP knockdown (Figure 8C). All three Cas13d encoding AAVs, (CasRx, hfCas13d, and DjCas13d) were capable of significantly reducing GFP mRNA levels indicating the functionality of these viruses (Figure 8D). We then explored the CMV-intron DjCas13d AAV for its ability to knockdown genes in either rats or mice. In rats, we evaluated DjCas13d in the knockdown of endogenous *BDNF* in the infralimbic (IL) cortex. An AAV encoding CMV-DjCas13d-HA and an AAV encoding a GFP gene, as well as a gRNA expression cassette designed to target *BDNF* or LacZ as a control were injected into the rat IL cortex (Figure 8E). Three weeks later, the transduced tissue was examined for knockdown of *BDNF* via qPCR (Figure 8F). DjCas13d significantly reduced *BDNF* mRNA expression levels compared to the control. The presence of the gRNA virus was confirmed with native GFP visualization and HA immunohistochemistry (IHC) was performed to examine DjCas13d-HA expression in the IL cortex (Figure 8G). Next, in mice, we injected the CMV-intron DjCas13d-HA AAV with an AAV encoding a GFP gene and a gRNA expression cassette against *Calcrl* into the right central nucleus of the amygdala (CeA) (Figure S9.). *Calcrl* is expressed in the central portion of the amygdala (CeA).[25] IHC showed colocalization of HA (DjCas13d) and native GFP (gRNA). *In situ* hybridization (ISH) for *Calcrl* with native GFP was used to evaluate the knockdown of *Calcrl* in the injected right CeA and the un-injected left CeA. Quantification of the *Calcrl* signal per cell in the CeA showed a significant decrease in *Calcrl* in the right CeA compared with the left CeA, suggesting that the combination of DjCas13d and gRNA against *Calcrl* was sufficient to knockdown mRNA expression on a cell-to-cell basis.

We also developed a few other AAVs that encode CasRx or hfCas13d but use the CMV enhancer fused to the chicken beta-actin promoter (CAG) or the eukaryotic translation elongation factor 1 α (EF-1α) promoter. Both of these promoters are very efficient at driving robust expression. We confirmed the functionality of these viruses for their ability to knockdown ectopically expressed GFP-*Calcrl* in 293FT cells (Figure S10.).

DISCUSSION

Precise and effective methods to manipulate gene expression are critical for correctly associating genes with their physiological function. There are numerous instances where RNA interference technology has led to the wrong conclusions due to the imprecise nature of RNAi.[2,26,27] Thus, it is imperative that new methods to manipulate gene expression are extensively vetted so their strengths and weaknesses are fully understood. The arrival of CasRx is a great example of a novel technology that needs to be viewed with caution. CasRx is extremely effective at knocking down genes, but its tendency to cleave bystander RNAs is a major shortcoming significantly reducing the utility of this technology. For many CasRx early adopters, this came as an unfortunate surprise, which in some cases led to the loss of research productivity due to spurious results and/or having to retool research approaches. Thankfully, new high-fidelity Cas13d variants have been identified that will presumably replace CasRx. Convincing evidence exists that both hfCas13d and DjCas13d possess similar abilities of knocking down genes compared to CasRx, but do so with

Figure 6. Cas13d vectors using a CMV promoter knock down ectopically expressed genes efficiently in neuro 2A cells

(A) Schematic of AAV vector maps depicting AAVs designed to express RfxCas13d, hfCas13d, or DjCas13d from a CMV promoter. Note these viral plasmids also encode an mCherry gene outside the viral genome region (not shown). (B) Graphic describing the outline of the experiment. (C) Neuro 2A cells were transfected with either of these Cas13d encoding vectors, a GFP-*Calcrl* encoding plasmid and a gRNA expression cassette, *Calcrl* 2, or a gRNA expression cassette designed to target LacZ as a control. Forty-eight hours post-transfection, the cells were imaged for GFP and mCherry epifluorescence. Representative images are shown. Visualization of images indicates a reduction of GFP-*Calcrl* levels in all of the *Calcrl* 2/Cas13d groups, while mCherry levels are reduced only in the *Calcrl* 2/CasRx and DjCas13d groups indicating collateral activity. (D and E) The RNA was isolated and subjected to whole transcriptome analysis using RNA-seq for samples treated with hfCas13d and DjCas13d. Volcano plots depict the differential expression observed between samples treated with *Calcrl* 2 gRNAs compared to LacZ gRNA controls. Gene lists were determined by filtering the data to only include genes with an adjusted p value of 0.1 and a fold change of ±1.4-fold. Blue dots indicate differentially expressed genes. Gray dots indicate genes that are not differentially expressed. The horizontal dashed line indicates the threshold for significance and the vertical dashed lines indicate the ±1.4-fold cut-off. GFP-*Calcrl* is indicated by a green square. The number of genes up and downregulated are indicated on each volcano plot. GFP-*Calcrl* was significantly and robustly knocked down in both hfCas13d (D) and DjCas13d (E) conditions, but the use of DjCas13d leads to hundreds of more transcripts being up and downregulated compared to using hfCas13d. (F) RNA-seq data was graphed to depict the relative changes in GFP-*Calcrl* RNA levels compared to LacZ controls. Both hfCas13d and DjCas13d were able to robustly knock down GFP-*Calcrl* and there were no significant differences between the hfCas13d and DjCas13d groups. (G) The use of DjCas13d/*Calcrl* 2 led to a significant reduction in mCherry RNA levels compared to hfCas13d (*$p < 0.05$). Notably, there was no reduction of mCherry mRNA in the hfCas13d/*Calcrl* 2 group. (H and I) Western blot indicates lower Cas13d protein levels of DjCas13d compared to hfCas13d, (H and J) reduction in GFP-*Calcrl* protein levels in both hfCas13d and DjCas13d, and (H and K) a reduction of mCherry protein levels only with DjCas13d when transfected with *Calcrl* 2 gRNA. Error bars = standard error of the mean. (F, I, and J) Two-way ANOVAs, *$p < 0.05$, (G and K) two-way ANOVAs followed by Tukey post-hoc, *$p < 0.05$. Three biological replicates for all groups ($n = 3$). Scale bars 400 microns, NTC, non-transfected control; B-tubulin, protein loading control.

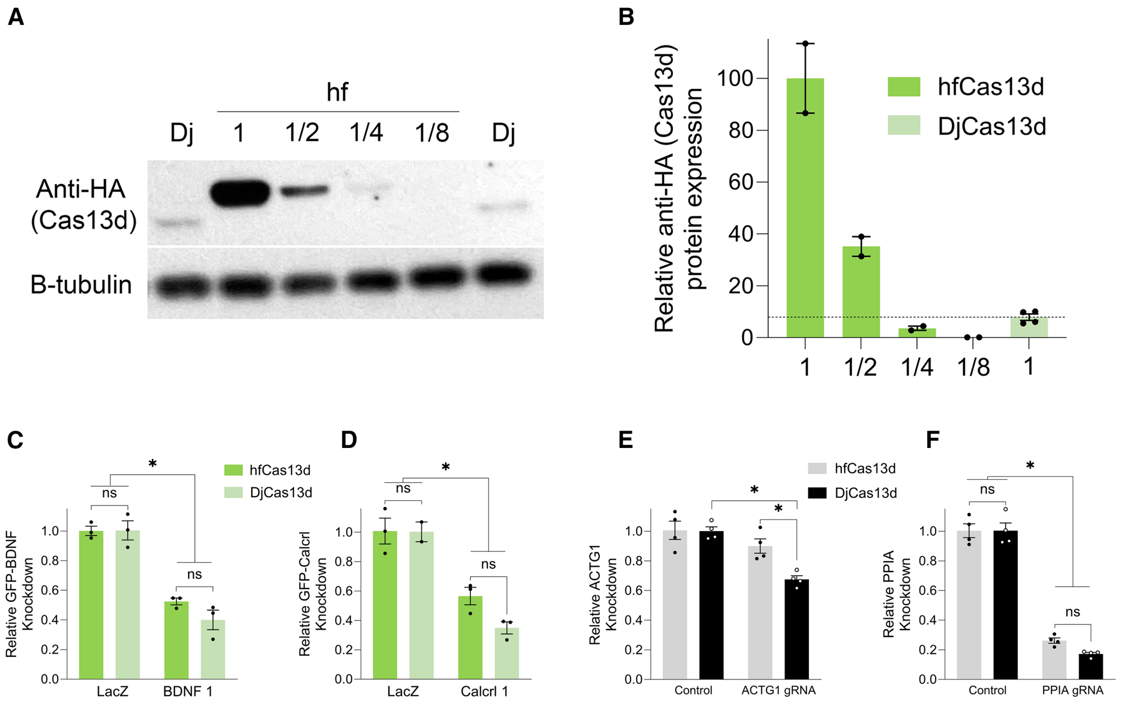

Figure 7. Knockdown of ectopically and endogenously expressed transcripts with equivalent amounts of hfCas13d and DjCas13d protein

(A) To determine the amount of hfCas13d and DjCas13d encoding plasmids needed to create equivalent levels of proteins, 293FT cells were transfected with differing amounts of hfCas13d encoding plasmids (1, 1/2, 1/4, or 1/8) while keeping the amount of DjCas13d encoding plasmid constant (1). Forty-eight hours post-transfection, the cells were harvested and homogenized for SDS-PAGE and western blotting. (B) The western blot quantification indicated that 1/3.5 or 28.5% of hfCas13d encoding plasmid was needed to create similar levels of DjCas13d protein levels. Error bars = standard error of the mean. Two biological replicates for the hfCas13d groups, and four biological replicates for the DjCas13d group (n = 2–4). To directly compare hfCas13d and DjCas13d for their efficacy, 293FT cells were transfected with the normalized amounts of hfCas13d and DjCas13d encoding plasmids, along with either a GFP-*BDNF* encoding plasmid and *BDNF* 1 or LacZ gRNA expression cassette plasmids, or GFP-*Calcrl* and *Calcrl* 1 or LacZ gRNA expression cassette plasmids, or either *ACTG1* or control gRNA expression cassette plasmids, or *PPIA* or control gRNA expression cassette plasmids. Forty-eight hours post-transfection, the RNA was isolated and subjected to qPCR to examine RNA levels for GFP-*BDNF*, GFP-*Calcrl*, *ACTG1*, or *PPIA*. (C) Both hfCas13d and DjCas13d produced similar knockdown levels of *BDNF*, and (D) *Calcrl*. (E) DjCas13d produced better knockdown of *ACTG1* compared to hfCas13d. (F) Both hfCas13d and DjCas13d produced similar knockdown levels of *PPIA*. (C, D, and F) Two-way ANOVAs, $^{*}p < 0.05$, (E) two-way ANOVA followed by Tukey post-hoc $^{*}p < 0.05$. (C and D) Two or three biological replicates for all groups (n = 2–3), (E and F) Four biological replicates for all groups (n = 4). B-tubulin, protein loading control.

significantly less cleaving of bystander RNAs. However, it was unknown how hfCas13d and DjCas13d compare to one another and if one had a more robust ability to knock down gene expression and also possessed a reduced tendency to disrupt the transcriptome. Our findings demonstrate that when targeting the same genes with the same spacer sequences under the same conditions, both hfCas13d and DjCas13d generally have similar efficacy in knocking down gene expression, but DjCas13d has a consistent tendency to induce much larger alterations of the transcriptome when targeting highly expressed genes. Based on previous data, neither DjCas13d nor hfCas13d appears to induce significant transcriptome alterations when targeting low to moderately expressed genes. Collectively, this indicates that DjCas13d likely could be used without concern when targeting lower expressed genes, but presumably, it appears to be a poor choice if high expressed genes are to be targeted.

When we started this line of research, we sought to generate several AAV vectors to deliver CasRx for gene knockdown purposes. We were surprised by the inexplicable loss of CasRx protein in conditions where CasRx was knocking down its intended target. Under these conditions, we observed that the protein levels of CasRx were significantly depleted as measured by western blot and by CasRx-mCherry epifluorescence. We also found no evidence that the ubiquitin-proteasome system was responsible for the loss of CasRx protein.

In our experiments comparing CasRx, hfCas13d, and DjCas13d for their ability to knockdown two ectopically expressed genes, *BDNF* and *Calcrl*, we observed that hfCas13d resulted in reduced loss of the Cas13d-mCherry epifluorescence signal in the targeting groups compared to both CasRx and DjCas13d groups. This indicated that hfCas13d exhibits less collateral cleavage compared to CasRx and DjCas13d under these circumstances. Analysis of the RNA from these samples, using an Agilent Bioanalyzer, revealed some RNA degradation in the CasRx targeting groups and, to a much lesser extent, in the hfCas13d, *Calcrl* 1 group. Specifically, two distinct RNA bands below the 28S rRNA band were present. It has been reported that these

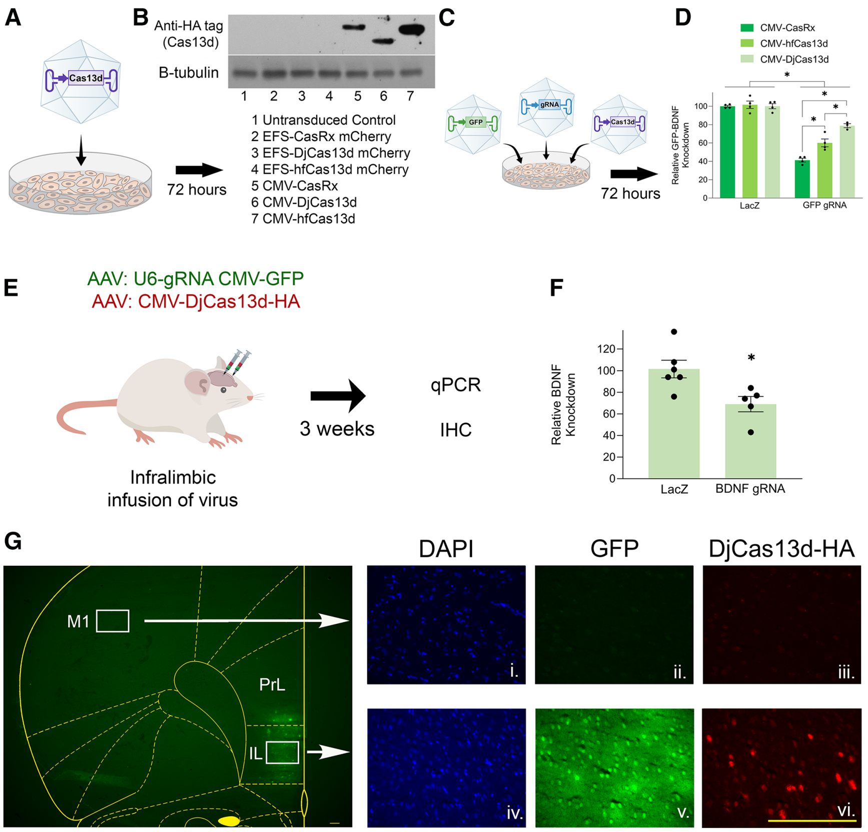

Figure 8. AAVs encoding Cas13d can knock down targets *in vitro* and *in vivo*

(A) The EFS-Cas13d-mCherry (Figure 3A) and the CMV-intron-Cas13d (Figure 6A) encoding AAVs were used to transduce 293FT cells in culture. Seventy-two hours later, the cells were harvested and Cas13d protein levels were examined via western blotting with an anti-HA tag antibody. (B) Only the cells that received the CMV-intron encoding Cas13d AAVs exhibited detectable expression of the HA-tagged Cas13d protein. B-tubulin levels were used as a loading control. $n = 1$. (C) CMV-intron encoding Cas13d AAVs, an AAV encoding a GFP gene, and AAVs either encoding a gRNA expression cassette designed to knockdown GFP or LacZ as a control were used to transduce 293FT cells in culture. Seventy-two hours later, the cells were harvested and qPCR was performed to assess GFP knockdown. (D) All three Cas13d encoding AAVs, (CasRx, hfCas13d, and DjCas13d) were capable of significantly reducing GFP mRNA levels, $n = 4$. (E) An AAV encoding CMV-DjCas13d and an AAV encoding a GFP gene with either a gRNA expression cassette designed to target *BDNF* or LacZ as a control were co-injected into the rat IL cortex and 3 weeks later, qPCR and IHC were performed. (F) Quantitative PCR revealed *BDNF* was significantly knocked down within the IL cortex compared to the LacZ control group, (*$p < 0.05$). LacZ gRNA group, $n = 6$; *BDNF* gRNA group, $n = 5$). Error bars = standard error of the mean. (G) The image on the left depicts a hemisected coronal brain slice taken from an animal that received the injection of viruses described (E and F). IL, infralimbic cortex; PrL, prelimbic cortex; M1, primary motor cortex. The location of viral transduction in the IL is obvious by visualizing native GFP fluorescence. Images to the right (i–vi) depict two magnified views from the brain slice and stained for DAPI, GFP fluorescence, and anti-HA DjCas13d expression IHC. Boxes to the top (i–iii) are from a control region from the M1 motor cortex that was not injected with virus. Boxes on the bottom (iv–vi) are magnified views of the viral transduction site. Scale bars are shown in vi., 200 microns. (D) Two-way ANOVA followed by Tukey post-hoc, *$p < 0.05$. (F) Independent t test, *$p < 0.05$. Error bars = standard error of the mean.

bands are the result of cleavage of the 28S rRNA by Cas13d. The presence of the bands was obvious, but it did not appear to alter the RNA integrity number of the RNA samples. Notably, the use of DjCas13d did not lead to a similar RNA degradation pattern, which was surprising because in other analyses, it was DjCas13d that seemed to have more off-target effects than hfCas13d. This may indicate that CasRx and hfCas13d possess some propensity to cleave the 28S rRNA in this manner. RNA-seq analysis revealed that DjCas13d was slightly better at knocking down *BDNF* compared to hfCas13d, but there was no difference in their ability to knockdown *Calcrl*, but DjCas13d led to the dysregulation of hundreds of more genes compared to hfCas13d. When we targeted two endogenous transcripts for knockdown, or an ectopically expressed transcript in neuro 2A cells with hfCas13d and DjCas13d, we also observed that the use of DjCas13d led to the dysregulation of many more genes compared to hfCas13d. These RNA samples did not exhibit any signs of rRNA degradation, based on the bioanalyzer results (data not shown).

Ontology analysis revealed that genes associated with the extracellular matrix were enriched in at least 3 out of the 4 downregulated gene lists from the DjCas13d experiments, and genes associated with RNA and ribosomal processing and regulation were enriched in these upregulated gene lists. However, the number of enriched genes were relatively few. We also cross-referenced genes found to be differentially expressed in our RNA-seq experiments to RNAs that could be potential off-targets due to sequence similarity to each gRNA that was used. Very few identified potential off-targets were determined to be differentially expressed in these experiments, and in the cases where the off-targets were differentially expressed, the sequence similarity was low, making it unlikely they were true off-targets. These findings are consistent with the notion that most of the transcriptional dysregulation caused by Cas13d is due to either collateral cleavage and/or other means that might alter transcription and/or RNA levels.

Several AAV vectors were developed for the delivery of Cas13d and its variants. Some of these were created using the weaker EFS promoter to drive expression of the Cas13d, while others were built using the strong CMV, CAG, and EF1a promoters. Notably, when we transduced equal amounts of Cas13d encoding AAVs into 293FT

cells, we found that we only observed Cas13d protein expression from the AAVs that used the CMV promoter, but not the EFS promoter in a western blot experiment. This is in part due to the fact that the EFS promoter is not as good at driving transcription compared to the CMV promoter and it is also a reflection that transducing the cells in this manner does so only moderately efficiently. Therefore, we recommend the use of AAVs that contain the strong promoters to drive Cas13d expression, since these should drive robust levels of Cas13d for optimal gene knockdown; however, the end user should decide which AAV plasmids best suits their experimental needs.

RNA-targeting Cas13d offers several advantages over the DNA-targeting CRISPR-Cas9 method for gene knockdown/knockout. CRISPR-Cas9 can be used to knock out genes, if it is deployed to create insertions or deletions (indels) within the protein-coding region of a gene. This is a powerful technique; however, due to its stochastic nature, it creates non-uniform mutations within and across cells. Cells targeted with CRISPR-Cas9 can result in mono- and bi-allelic mutations or none at all, and the mutations created are different.[28] Targeting cells *in vivo* with this technology will result in a highly heterogeneous population of cells that can potentially negatively impact research outcomes. In contrast, Cas13d should create homogenous gene knockdown across a population of targeted cells, and when using the appropriate Cas13d variant such as hfCas13d, there should be very low, if any, gene dysregulation, even when targeting very highly expressed transcripts. We suspect the AAV vectors produced in this study should be very useful for those interested in gene knockdown *in vivo* and *ex vivo*.

MATERIALS AND METHODS

Recombinant DNA cloning

Standard recombinant cloning techniques, including Gibson assembly, were used to create all novel recombinant DNA plasmids. All novel plasmids were created using AAV plasmids and were confirmed by whole plasmid sequencing (Plasmidsaurus). Most of these plasmids were deposited at Addgene plasmid repository. All other novel plasmids described within are available upon request. The DNA sequence of RfxCas13d (CasRx) was derived from pXR001: EF1a-CasRx-2A-EGFP, Addgene plasmid no. 109049. CasRx-compatible gRNA expression cassettes used in this study were derived from pXR003: CasRx gRNA cloning backbone, Addgene plasmid no. 109053. High-fidelity Cas13d (hfCas13d) plasmids were created by exchanging a portion of the CasRx sequence (**GCC**GAATACATTACCAAC**GCCGCC**TAC**GCC)** with the following DNA sequence, (5′-**GTG**GAATACATTACCAAC**GTGGTG**TAC**GTG**3′) to recapitulate the CasRx N2V8 mutant leading the conversion of 4 alanine codons to valine codons (**V**EYITN**VV**Y**V**) (marked in bold).[20] Plasmids designed to express DjCas13d were created from a synthesized gBlock (Integrated DNA Technologies) encoding a human codon-optimized DjCas13d sequence derived from the reverse translated publicly available amino acid sequence of DjCas13d.[21] DjCas13d compatible gRNA expression cassettes were created using the same architecture as the CasRx gRNA expression cassettes but instead used the direct-repeat (DR) specifically for DjCas13d. All gRNA expression cassettes were designed to accept guide sequences via SapI sites that occur immediately after the DR. Nuclear-localized enhanced blue-fluorescent protein 2 expressing plasmids were derived from Addgene Plasmid no. 14893.[29] The protein-coding regions of rat *BDNF*, rat *adrenoceptor alpha 2A* (*ADRA2A*), and mouse *calcitonin receptor-like receptor* (*Calcrl*) were PCR amplified from complementary DNA (cDNA) and ligated into pAAV-EFS-GFP-pA, Addgene plasmid no. 62913. *BDNF* and *ADRA2A* protein-coding regions were ligated into the EFS-GFP plasmid to create N-terminal fusions with GFP and these plasmids were used in experiments described in Figure S4. Additionally, the protein-coding regions of *BDNF* and *Calcrl* were ligated into the EFS-GFP plasmid immediately after the GFP stop codon to create transcripts that contain the *BDNF* and *Calcrl* sequences, but would only express GFP protein. This design enabled GFP expression and localization to remain unaltered but provided the necessary gRNA binding sites for *BDNF* and *Calcrl* targeting gRNAs. Two different control gRNAs were used throughout this study. One control gRNA was designed to target the LacZ gene that encodes beta-galactosidase, and is not present in the human genome. For each experiment that this control was used in, it is explicitly referred to as the LacZ control gRNA. The other control gRNA was a 17-nucleotide long sequence that is part of the empty gRNA expression cassettes prior to a custom gRNA being inserted into it. This sequence occurs immediately after the DR and immediately before the RNA polymerase transcriptional termination signal. Studies have indicated that gRNA sequences of 17 nucleotides or less possess little to zero ability to cleave intended targets.[21,30] We refer to this gRNA as a control gRNA. The plasmid pAAV-Ef1a-mCherry was used in Figure S3 and was also used as a source for the mCherry gene used in some of the plasmids described in this study. It was a gift from Karl Deisseroth (Addgene plasmid no. 114470; http://n2t.net/addgene:114470; RRID:Addgene_114470). A complete list of plasmids used and which experiment they were used in are in Table S5. All gRNA sequences are provided in Figure S11.

DNA transfections

293FT cells (Invitrogen cat no. R70007) or Neuro-2A (N2A) cells (ATCC, cat no. CCL-131), were grown in cell culture following the manufacturers' growth conditions. Twenty-four hours prior to DNA transfection, cells were seeded at 150,000 cells per well, in a 24-well cell culture plate. Equal amounts of each plasmid totaling no more than 800 nanograms were transfected into cells using Lipofectamine 2000 (Invitrogen) following the manufacturer's instructions. Cells were harvested 24 or 48 h later as specified. For experiments designed to examine differences between Cas13d variants, target encoding plasmids (if applicable) for each experiment were diluted into a master mix of Optimem first and then after vortexing, were aliquoted into appropriate tubes. Next, the Cas13d and gRNA encoding plasmids were added, and these transfections were conducted during the same session to minimize technical variability. For the experiment depicted in Figure 7, in cases where a lower amount of the hfCas13d encoding plasmid was used, a corresponding amount of puc19

plasmid was used to keep the total amount of plasmid DNA constant per transfection.

Microscopy

Cells grown in a 24-well plate were live imaged at 100× magnification on a Keyence BZ-X810 microscope and representative images were captured at high resolution setting. All BFP, mCherry (RFP), and GFP images were captured using the same exposure conditions within each experiment, respectively. Images were quantified for brightness using the Keyence BZ-X810 Analyzer Software. Images used to generate data for each figure were imaged during the same session. For the experiment depicted in Figure S5, 293FT cells were transfected with Cas13d-mCherry plasmids. Twenty-four hours later the media was removed, and the cells were fixed in 500 μL of 4% paraformaldehyde (PFA) in 1 × PBS (pH 7.4) on ice. After 25 min, all the liquid was removed from each well and the cells were cover-slipped using ProLong Diamond Antifade Mountant with DAPI (Invitrogen) and the cells were imaged. For Figure 8G, brain tissue images were taken on cryocut sections from a freshly frozen brain and imaged at 20×. IHC was performed on tissue slices and imaged at 400×.

Drugs

To inhibit the ubiquitin proteasome system, 293FT cells were treated 6 h after transfection with either MG132 (10 μM) (MilliporeSigma, cat no. M8699), or clasto-lactacystin β-lactone (1 μM) (MilliporeSigma, cat no. L7035). Cells were treated with leupeptin (25 μgs/ul) (MilliporeSigma, cat no. L2884) to inhibit the lysosome. Concentrations were selected based on concentrations that have been previously described.[31]

RNA purification

For experiments described in Figure 2, 293FT cells were homogenized in 1 mL of Trizol reagent (Invitrogen) per well, and the RNA was isolated following the manufacturer's standard procedure. The RNA was then DNAse treated in a 100 μL reaction using the RNase-Free DNase Set Kit following the manufacturer's recommendations (QIAGEN, cat no. 79254). Subsequently, the RNA was purified using the RNeasy MinElute Cleanup Kit, (QIAGEN, cat no. 74204). For all other experiments, cells were homogenized in 300 μL of Trizol reagent, brain tissue was homogenized in 500 μL of Trizol reagent, and the RNA was purified and DNAse treated using the Direct-zol RNA MiniPrep Plus Kit (Zymo Research, cat no. R2072), and the RNA was eluted in 25–50 μL of nuclease-free water.

Quantitative PCR

Total RNA was converted to cDNA using either the QuantiTect Reverse Transcription Kit (QIAGEN, cat no. 205311) or the iScript cDNA synthesis kit (Bio-Rad, cat no. 1708891), following the manufacturer's instructions. Complementary DNA synthesis occurred for 25 min, before the reaction was inactivated and then 0.3–1 μL of each reaction was used for 10–20 μL qPCR reactions using the QuantiTect SYBR Green PCR Kit (QIAGEN, cat no. 204143) following the manufacturer's instructions. Amplification occurred on a 384-well ABI QuantStudio 12K Flex system with OpenArray block qPCR machine using the following amplification parameters: activation: 95°C for 15 min; 40 cycles of denaturation: 94°C for 15 s, annealing: 55°C for 30 s, extension: 72°C for 30 s, with fluorescence data collection only during the extension phase. For all qPCR experiments, all samples were run in triplicate and relative gene concentrations were normalized against *ATP5PB*(*ATP5F1*)[32] or *GAPDH* and data were analyzed using the delta-delta Ct method. qPCR primers: (GFP: FP 5′-AAGCTGACCCTGAAGTTCATCTGC-3′; reverse primer (RP) 5′-CTTGTAGTTGCCGTCGTCCTTGAA-3′), (BFP: FP-5′ AAGCTGACCCTGAAGTTCATCTGC-3′; RP 5′-CTTGTAGGTGCCGTCGTCCTTGAA-3′), (human *ATP5F1*: FP 5′-TTAGCGCAGAGACCTTCACT-3′; RP 5′-CCAGTGCCTGTTGTGACTTC-3′), (human *GAPDH*: FP 5′-GGATTTGGTCGTATTGGG-3′; RP 5′-GGAAGATGGTGATGGGATT-3′), (rat *BDNF*: FP 5′-AAGGCTGCAGGGGCATAGAC-3′; RP 5′-TGAACCGCCAGCCAATTCTC-3′), (rat *GAPDH*: FP 5′-GCATCCTGCACCACCAACTG-3′; RP 5′-ACGCCACAGCTTTCCAGAGG-3′), (mouse *Calcrl*: FP 5′-CGGATGGCTATGCTGGAATGAC-3′; RP 5′-GGATGCCGAAACCAGTGTCCAT-3′), (human *PPIA*: FP 5′-TCTGCACTGCCAAGACTGAG-3′ ; RP 5′-TCGAGTTGTCCACAGTCAGC-3′), human *ACTG1*: FP 5′-AGGCCAACAGAGAGAAGATGACT-3′; RP 5′-CGTCTCCAGAGTCCATGACA-3′).

Western blotting

Cells were homogenized in 100 μL of RIPA lysis buffer (Sigma, cat no. RQ0278) containing protease inhibitor cocktail (1 tablet per 10 mL lysis buffer), (Roche, EASYpack, cat no. 4693124001), and 1 mM PMSF, (Roche, cat no. 10837091001), using a tissue grinder (Kontes Glass CO. no. 18) on ice. The supernatant was extracted after a 15-min 16,300 G centrifugation at 4°C, and the total protein was quantified using a BCA Assay Kit (Pierce, cat no. 23227). Equal protein amounts of each sample per gel were electrophoresed on a 4–15% SDS-PAGE Precast Gel (Bio-Rad, cat no. 5678085) and transferred to polyvinylidene fluoride membranes at 50 V for 45 min at room temperature with Tris/glycine transfer buffer (Bio-Rad, cat no. 1610734). Membranes were rinsed in T-TBS (20 mM Tris-HCl, 50 mM NaCl, 0.1% Tween 20) for 20 min and blocked with BlockHen (AVES, cat no. BH-1001) 1:10 in PBS followed by 15 min wash with D-PBS for chicken Anti-GFP (AVES, cat no. GFP-1020), or EveryBlot (Bio-Rad, cat no. 12010020) or 5% BSA (Sigma A7906) in 1× PBS for mouse Anti-HA (CasRx) (BioLegend, cat no. 901501), and mouse Anti-β-tubulin (Sigma, cat no. T7816). Primary antibody incubation occurred overnight at 4°C as follows: anti-GFP 1:10,000 in PBS 0.1% Tween 20, anti-HA 1:2,000, and anti-β-tubulin 1:25,000, in EveryBlot or 5% BSA. The membranes were then rinsed three times with T-TBS and probed with secondary antibody 1:10,000 in EveryBlot or 5% BSA for 1 h at room temperature: anti-mouse IgG horseradish peroxidase (HRP)-linked (Cell Signaling, cat no. 7076) (for anti-HA, and anti-β-tubulin), or anti-rabbit IgG HRP-linked (Cell Signaling, cat no. 7074) for anti-chicken IgY HRP-linked (Abcam, cat no. 6877) for anti-GFP. The membranes were rinsed three times in T-TBS and once in Tris-buffered saline (TBS) (20 mM Tris-HCl, 50 mM NaCl), and then developed using the enhanced chemiluminescent

(ECL) Chemiluminescence assay (Thermo Fisher Scientific, cat no. 34580). Images were captured at 12 MegaPixels, and the protein bands were quantified by averaged intensity density in Adobe Photoshop. The Integrated Density of target bands was normalized by β-tubulin integrated density for each sample.

RNA-seq

RNA-seq libraries were prepared using the Illumina Stranded mRNA Prep Ligation kit (Illumina) as per the manufacturer's instructions. Briefly, polyA RNA was purified from 200 ng of total RNA using oligo (dT) beads. The extracted mRNA fraction was subjected to fragmentation, reverse transcription, end repair, 3′-end adenylation, and adaptor ligation, followed by PCR amplification and magnetic bead purification (Omega Bio-Tek). The unique dual index sequences (IDT for Illumina RNA UD Indexes Set B, Ligation) were incorporated in the adaptors for multiplexed high-throughput sequencing. The final product was assessed for its size distribution and concentration using BioAnalyzer High Sensitivity DNA Kit (Agilent Technologies). The libraries were pooled and diluted to 1 nM and then denatured using the Illumina protocol. The denatured libraries were loaded onto an SP flow cell on an Illumina NovaSeq 6000 (Illumina) and run for 2 × 59 cycles according to the manufacturer's instructions, to obtain an average of 25 million reads per sample. Fastq files were uploaded to BasePairTech.com and aligned to a custom reference genome created from either the soft-masked primary assembly of the human GRCh38 genome or the mouse mm39 genome, modified to include genes that were ectopically expressed in 293FT and Neuro 2A cells in this study. Expression counts were determined using STAR and differential expression was determined using DESeq2. Following DESeq2, differentially expressed gene lists were determined by filtering the data to only include genes with an adjusted p value of 0.1 and a fold change of ±1.4-fold. All RNA-seq experiments were conducted using 3 biological replicates (n = 3). Samples used to generate RNA-seq data presented in Figures 2 and 4 were run on the same sequencing flow cell. Samples used to generate RNA-seq data presented in Figures 5 and 6 were run on their own sequencing flow cells.

Determination of transcript abundance

To determine the relative abundance of *PPIA* and *ACTG1* in 293FT cells with respect to the entire transcriptome, the RNA-seq data depicted in Figure 4A was examined. Specifically, the TPMs for every gene were averaged for the three RNA-seq samples for the hfCas13d, LacZ group and sorted for abundance. We then determined the number of genes with TPMs higher than 1,000, 100, 10, 1, and .1. The number of genes included in these groups, was 87, 1,302, 9,715, 10,451, and 10,526 respectively. Therefore, ACTG1 (∼1,200 TPM) and PPIA (∼160 TPM) are in the top 5% of transcripts for abundance.

Gene ontology analysis

Differential expression gene lists from the RNA-seq experiments were analyzed to determine if specific classes of genes were enriched in each list. Enriched gene ontology was determined using Enrichr[33–35] and gene classes for biological, cellular, and molecular gene ontology were reported in cases of adjusted p values of less than 0.05. To create Venn diagrams and combined gene lists, Draw Venn Diagram (ugent.be) was used.

Off-target analysis

Potential off-targets for gRNAs used in RNA-seq experiments were identified by searching the human GRCh38 or mouse GRCm39 (cDNAs [transcripts/splice variants]), database using BlastN. Specifically, each gRNA target sequence was searched against the applicable database using the following parameters for 23 nucleotide long gRNAs: maximum number of hits to report = 50,00, maximum E-value for reported alignments = 100,000, word size for seeding alignments = 5, match/mismatch scores = 3/−2, gap penalties = (opening = 5, extension = 5). Gaps were allowed and no filtering for low complexity regions was conducted. Similar settings were used for gRNAs that were 30 nucleotides, except the maximum E-value was set to 10,000. The resultant gene lists were cross referenced against the differentially expressed gene lists for the associated RNA-seq experiment using Draw Venn Diagram (ugent.be). The alignments for genes that were on both lists were examined and final gene lists were created by including only genes where the sequences were in the correct orientation to bind to the gRNA (Table S4).

Adeno-associated virus production for *in vivo* use

AAV-CMV-intron-DjCas13d-pA, AAV-DR(BDNF 1 gRNA)-CMV-intron-GFP-pA, AAV-DR(Calcrl 1 gRNA)-CMV-intron-GFP-pA and AAV-DR(LacZ gRNA)-CMV-intron-GFP-pA, were used to make *in vivo* grade AAVs pseudotyped as AAV/DJ8 suitable for intracranial injection into the rodent using a triple transfection, helper-free method as previously described.[36] Plasmids encoding pRC-AAVDJ/8 (Cell BioLabs), pHelper, and AAV2 genome plasmids were transfected into 293FT cells (Invitrogen, cat no. R70007). The transfection was performed using 810 uls of PEI Max (Polysciences, Inc.), at 1 mg/mL and 90 ugs of each of the three plasmids per 5 × 15 cm plate transfection. Seventy-two hours post-transfection, the cells were harvested, and lysed using a freeze-thaw method, DNA was degraded using Benzonase (Millipore-Sigma), and AAVs were purified on an iodixanol step gradient via ultracentrifugation and underwent buffer exchange (1× PBS, 0.001% Pluronic F-68, 200 mM NaCl) and concentration using Amicon Ultra-15 filter units (Millipore Sigma, cat no. C7715).

Adeno-associated virus production for *in vitro* use

AAV2-based viral genome plasmids were used to make AAVs pseudotyped as AAV/DJ suitable for cell culture experiments. Plasmids encoding pRC-AAVDJ (Cell BioLabs), pHelper, and AAV2 genome plasmids were transfected into 293FT cells (Invitrogen, cat no. R70007) in 6-well plates using Lipofectamine 2000 (Invitrogen), following the manufacturer's instructions. The day after the transfection, the media was removed and replaced for each well. Seventy-two hours post-transfection the cell media was collected and centrifuged to remove cell debris. The resultant viral supernatants were titered to calculate the concentration of DNase-resistant viral particles and

aliquots of the virus were made and stored at −20°C until use. Viral particles (5×10^9) were added to each well of a 24 well plate where applicable.

Viral titering

Viruses were titered to calculate the concentration of DNAse-resistant viral particles using a SYBR green (QIAGEN) quantitative PCR strategy as previously described.[36] PCR primers designed to anneal to either the CMV promoter: (FP 5′-GGCGGAGTTGTTAC GACAT-3′; RP 5′-GGGACTTTCCTACTTGGCA-3′), the GFP coding region: (FP 5′-AAGCTGACCCTGAAGTTCATCTGC-3′; RP 5′-CTTGTAGTTGCCGTCGTCCTTGAA-3′), CasRx/hfCas13d coding region: (FP 5′-GACGCCCCTAGACTGCCCAG-3′; RP 5′-GCGGAAACATCACGGCCAGC-3′), or the mCherry coding region: FP 5′-TCATCAAGGAGTTCATGCGC-3′; RP 5′-CCACCCT TGGTCACCTTCAG-3′), were used to titer the viruses.

Stereotaxic injection of adeno-associated virus

In vivo rat experiments were approved by the Institutional Animal Care and Use Committee (IACUC) from University of Texas at Dallas. Sprague Dawley male rats received a bilateral infusion (0.8 μL each side) of a 1:1 mixture of AAVDJ/8-CMV-intron-DjCas13d-pA and AAVDJ/8-DR(BDNF 1 gRNA)-CMV-intron-GFP-pA for *BDNF* KD or a 1:1 mixture of AAVDJ/8-CMV-intron-DjCas13d-pA and AAVDJ/8-DR(LacZ gRNA)-CMV-intron-GFP-pA as a control in the IL portion of the mPFC. Each virus was at a concentration of $\sim 1 \times 10^{13}$ genome copies (GC)/ml prior to mixing 1:1. For infusion surgery, rats were anesthetized with a ketamine (85 mg/kg) and xylazine (5 mg/kg) mixture intraperitoneally and head-fixed using a stereotaxic apparatus. An incision on the scalp was made to expose the skull and drill two burr holes. A Hamilton syringe was used to infuse 0.8 μL of the virus mixture into the IL using the following coordinates from bregma: +3.0 A/P, ±0.6 M/L, −5.3 V/D. Rats were sacrificed 3 weeks after viral infusion, brains were removed and were fresh frozen on dry ice for (1) visualization of native GFP (gRNA expression), (2) visualization of HA-tag (DjCas13d expression) by IHC (see following text), or (3) analysis of *BDNF* mRNA knockdown by qPCR (see previous text).

In vivo mouse experiments were approved by the IACUC of the University of Texas at Dallas (protocol no. 20-04). C57BL6/J wild-type mice received a unilateral viral injection into the right central nucleus of the amygdala (CeA). The injection consisted of a 1:1 mixture of AAVDJ/8-CMV-intron-DjCas13d-pA and AAVDJ/8-DR(gRNA 3)-CMV-intron-GFP-pA for *Calcrl* knockdown. Each virus was at a concentration of $\sim 1 \times 10^{13}$ GC/mL prior to mixing 1:1. For stereotaxic surgery, mice were briefly knocked out in a jar using isoflurane and then head-fixed on a stereotaxic frame (Kopf, Model 1900) under 3% isoflurane at a flow rate of 250 mL/min. The isoflurane rate was then adjusted to 1.6%–1.8% with a flow rate of 150 mL/min and maintained throughout the surgery. An incision was made along the head to expose the skull. The head was aligned between bregma and lambda in the dorsal place using an alignment indicator. The coordinates from bregma (−1.45 A/P, ±3.00 M/L, −4.30 V/D) were used to mark the injection site and to drill a small hole for viral injection. A volume of 1 μL of the virus mixture was delivered into the right CeA using a Hamilton syringe connected to a glass pipette and a microinjection syringe pump (WPI, MICRO2T) through the drilled hole. Mice were sacrificed 2 weeks after viral injection and perfused with an ice-cold 1× phosphate buffered saline (PBS) followed by ice-cold 4% PFA in 1× PBS. Brains were extracted and stored in 4% PFA overnight at 4°C before being transferred to a 20% sucrose solution. A small cut was made on the injection side (right side) of the brain to mark that side as the “right hemisphere.” The brains were then embedded in optimal cutting temperature compound (OCT) in a plastic mold and frozen in a dry-iced ethanol bath. This preparation allowed for (1) visualization of native GFP (gRNA expression), (2) visualization of HA-tag (DjCas13d expression) by IHC, and/or (3) analysis of *Calcrl* mRNA knockdown using RNAScope ISH as described in the following text.

Immunohistochemistry

For the rat study, fresh frozen brains were sectioned on a cryostat, and 20 μm coronal sections were taken and stored immediately at −80°C. The slices were thawed in a humid chamber on the day of the IHC. A circle around the slices was drawn with a hydrophobic pen to contain the solutions on the slides. After the hydrophobic ink dried, each slice was fixed with fresh, filtered cold 4% PFA in 1× PBS (pH 7.4) for 5 min, washed three times with 1× PBS (pH 7.4) solution, and blocked with 120 μL of freshly made blocking solution (5% goat serum, 0.3% Triton X-100, 1× PBS (pH 7.4) for 1 h at room temperature. Then, the blocking solution was replaced by a 120 μL blocking solution containing the primary antibody at a 1:800 dilution (Cell Signaling HA-tag C29F4 rabbit mAB3724). The slides were stored in a humid chamber wrapped in foil at 4°C overnight. On the next day, the slices were washed three times with 1× PBS (pH 7.4) at room temperature, then incubated in freshly made blocking buffer with the secondary antibody at a 1:1,000 dilution (TexasRed goat anti-rabbit IgG (H + L), Invitrogen, cat no. A-11008), for 2 h at room temperature, in the dark. After the incubation, the slices were washed three times with 1× PBS (pH 7.4), and the slides were mounted in the dark with ProLong Diamond Antifade Mountant with DAPI (Invitrogen, cat no. P36962). Slides were dried in the dark for 24 h before imaging.

For the mouse study, a perfused frozen brain was sectioned using a cryostat (CM 1850UV, Leica). All brain sections containing the CeA were collected alternatively at 30 μm thickness for IHC and 15 μm thickness for RNAscope ISH. The brain sections for IHC were stored in 1× PBS at 4°C until use, while sections for ISH were directly mounted on glass slides (VWR, Superfrost) and stored at −80°C until use. Brain sections for IHC were washed twice in 1× TBS for 5 min each and treated with 3% H_2O_2 for 10 min at room temperature. After washing again in 1× TBS, sections were incubated with blocking buffer (5% normal goat serum, 0.3% Triton X- in 1× TBS) for 60 min at room temperature. The blocking buffer was replaced with primary antibody (1:1,000 mouse anti-HA, ABclonal AE008) diluted in

blocking buffer and incubated at 4°C overnight. Sections were washed once in 1× TRIS-buffered saline with Triton (TBST) (0.3% Triton X- in 1× TBS) and three times in 1× TBS for 5 min each. Sections were then incubated with secondary antibody (1:250 goat anti-mouse Alexa 555, Thermo Fisher Scientific, cat no. A21422) for 1 h at room temperature. After a series of washing as described earlier, all brain sections were mounted on glass slides (VWR, Superfrost) and coversliped with Vectashield anti-fade mounting medium containing DAPI (Vector Laboratories, H-1800-10). The following day, slides were imaged to validate the injection target and visualize the native GFP present in the right CeA using the microscope (Olympus, IX83 Inverted microscope).

RNAScope *in situ* hybridization

Brain sections adjacent to those showing native GFP and HA tag in right CeA during IHC were selected for RNAScope ISH. The RNAscope fluorescent multiplex assay v2 kit (ACD, no. 323110) and Calcrl probe (ACD, no. 452281) were used to validate Calcrl expression knockdown following the manufacturer's protocol. A negative probe (ACD, no. 320871) was used as a control. Perfused frozen brain sections were briefly rinsed in 1× PBS (pH 7.4) to remove OCT, then dehydrated through an ethanol series (50%, 70%, and 100% twice) at room temperature. After air-drying, hydrophobic barriers were drawn around the sections using ImmEdge pen (Vector Lab, H-4000). Protease III was applied and incubated for 20 min at 40°C in the humidity-controlled HybEZ II oven. Sections were washed twice in 1× wash buffer (ACD, no. 310091) before hybridization with Calcrl probe (ACD, no. 452281) and negative probe (ACD, no. 320871) for 2 h at 40°C. After washing, amplification steps (AMP1, AMP2, and AMP3) were performed at 40°C for 30, 30, and 5 min, respectively, with washing between steps. HRP signal for C1 was developed by incubating for 15 min at 40°C. Opal dye 690 (AKOYA, FP1497001KT) diluted in Tyramide Signal Amplification (TSA) buffer (ACD, no. 322809) was applied and incubated for 30 min at 40°C. After washing, HRP signal was blocked with the HRP blocker for 15 min at 40°C. Slides were coversliped with DAPI-containing anti-fade mounting medium (Vector Laboratories, H-1500-10). The following day, slides were imaged using a confocal laser microscope (Olympus, FV 3000 RS) at 20× to image the entire CeA and 40× to capture the close-up CeA structure. The *Calcrl* RNA expression and GFP signal were quantified by measuring pixel density for all cells within the field of view that showed any *Calcrl* signal associated with a clear DAPI-labeled nucleus in both the right and left CeA. The left CeA was served as a control with no viral injection.

DATA AVAILABILITY

The data that support the findings of this study are available from the corresponding author upon reasonable request.

ACKNOWLEDGMENTS

We thank the Ploski laboratory members for their support and assistance with this project, including the editing of this manuscript. We thank Mary O'Brien, Rachel Barkey, Laura Odom, and Neha Philip for their technical assistance. This work was supported by the National Institutes of Health grants, R01MH120302 (J.E.P.), R01DA055008 (S.K.), R01 DK115478 (B.J.K.), and F31 NS129269 (V.M.H.). We thank the Penn State College of Medicine Genome Sciences Core for their technical assistance and for performing the RNA quality control and RNA-seq library preparation and sequencing. We thank Simplified Science Publishing for making the illustrations for the figures.

The Genome Sciences Core (RRID:SCR_021123) services and instruments used in this project were funded, in part, by the Pennsylvania State University College of Medicine via the Office of the Vice Dean of Research and Graduate Students and the Pennsylvania Department of Health using Tobacco Settlement Funds (CURE). The content is solely the responsibility of the authors and does not necessarily represent the official views of the University or College of Medicine. The Pennsylvania Department of Health specifically disclaims responsibility for any analyses, interpretations, or conclusions.

AUTHOR CONTRIBUTIONS

J.E.P. provided the conceptual framework for the study. J.E.P., F.B., B.J.K., and S.K. designed the experiments. F.B., J.E.P., A.S., L.M.V., V.M.H., A.B., S.R.R., and J.T.O. performed the experiments. J.E.P. and F.B. wrote the manuscript with editorial feedback from B.J.K., S.K., and A.B. J.E.P., B.J.K., and S.K. received funding to complete the study.

DECLARATION OF INTERESTS

The authors declare no competing interests.

SUPPLEMENTAL INFORMATION

Supplemental information can be found online at https://doi.org/10.1016/j.omtm.2025.101565.

REFERENCES

1. Jackson, A.L., Bartz, S.R., Schelter, J., Kobayashi, S.V., Burchard, J., Mao, M., Li, B., Cavet, G., and Linsley, P.S. (2003). Expression profiling reveals off-target gene regulation by RNAi. Nat. Biotechnol. *21*, 635–637. https://doi.org/10.1038/nbt831.
2. Smith, I., Greenside, P.G., Natoli, T., Lahr, D.L., Wadden, D., Tirosh, I., Narayan, R., Root, D.E., Golub, T.R., Subramanian, A., and Doench, J.G. (2017). Evaluation of RNAi and CRISPR technologies by large-scale gene expression profiling in the Connectivity Map. PLoS Biol. *15*, e2003213. https://doi.org/10.1371/journal.pbio.2003213.
3. Sigoillot, F.D., Lyman, S., Huckins, J.F., Adamson, B., Chung, E., Quattrochi, B., and King, R.W. (2012). A bioinformatics method identifies prominent off-targeted transcripts in RNAi screens. Nat. Methods *9*, 363–366. https://doi.org/10.1038/nmeth.1898.
4. Goel, K., and Ploski, J.E. (2022). RISC-y Business: Limitations of Short Hairpin RNA-Mediated Gene Silencing in the Brain and a Discussion of CRISPR/Cas-Based Alternatives. Front. Mol. Neurosci. *15*, 914430. https://doi.org/10.3389/fnmol.2022.914430.
5. Abudayyeh, O.O., Gootenberg, J.S., Konermann, S., Joung, J., Slaymaker, I.M., Cox, D.B.T., Shmakov, S., Makarova, K.S., Semenova, E., Minakhin, L., et al. (2016). C2c2 is a single-component programmable RNA-guided RNA-targeting CRISPR effector. Science *353*, aaf5573. https://doi.org/10.1126/science.aaf5573.
6. Abudayyeh, O.O., Gootenberg, J.S., Essletzbichler, P., Han, S., Joung, J., Belanto, J.J., Verdine, V., Cox, D.B.T., Kellner, M.J., Regev, A., et al. (2017). RNA targeting with CRISPR-Cas13. Nature *550*, 280–284. https://doi.org/10.1038/nature24049.
7. Konermann, S., Lotfy, P., Brideau, N.J., Oki, J., Shokhirev, M.N., and Hsu, P.D. (2018). Transcriptome Engineering with RNA-Targeting Type VI-D CRISPR Effectors. Cell *173*, 665–676.e14. https://doi.org/10.1016/j.cell.2018.02.033.
8. Cox, D.B.T., Gootenberg, J.S., Abudayyeh, O.O., Franklin, B., Kellner, M.J., Joung, J., and Zhang, F. (2017). RNA editing with CRISPR-Cas13. Science *358*, 1019–1027. https://doi.org/10.1126/science.aaq0180.
9. Zheng, Z., Li, G., Cui, C., Wang, F., Wang, X., Xu, Z., Guo, H., Chen, Y., Tang, H., Wang, D., et al. (2022). Preventing autosomal-dominant hearing loss in Bth mice with CRISPR/CasRx-based RNA editing. Signal Transduct. Target. Ther. *7*, 79. https://doi.org/10.1038/s41392-022-00893-4.
10. Mahas, A., Aman, R., and Mahfouz, M. (2019). CRISPR-Cas13d mediates robust RNA virus interference in plants. Genome Biol. *20*, 263. https://doi.org/10.1186/s13059-019-1881-2.

11. Hernandez-Huertas, L., Kushawah, G., Diaz-Moscoso, A., Tomas-Gallardo, L., Moreno-Sanchez, I., da Silva Pescador, G., Bazzini, A.A., and Moreno-Mateos, M. A. (2022). Optimized CRISPR-RfxCas13d system for RNA targeting in zebrafish embryos. STAR Protoc. *3*, 101058. https://doi.org/10.1016/j.xpro.2021.101058.

12. Wang, Q., Liu, Y., Han, C., Yang, M., Huang, F., Duan, X., Wang, S., Yu, Y., Liu, J., Yang, H., et al. (2021). Efficient RNA Virus Targeting via CRISPR/CasRx in Fish. J. Virol. *95*, e0046121. https://doi.org/10.1128/JVI.00461-21.

13. Kushawah, G., Hernandez-Huertas, L., Abugattas-Nuñez del Prado, J., Martinez-Morales, J.R., DeVore, M.L., Hassan, H., Moreno-Sanchez, I., Tomas-Gallardo, L., Diaz-Moscoso, A., Monges, D.E., et al. (2020). CRISPR-Cas13d Induces Efficient mRNA Knockdown in Animal Embryos. Dev. Cell *54*, 805–817.e7. https://doi.org/10.1016/j.devcel.2020.07.013.

14. Morelli, K.H., Wu, Q., Gosztyla, M.L., Liu, H., Yao, M., Zhang, C., Chen, J., Marina, R.J., Lee, K., Jones, K.L., et al. (2023). An RNA-targeting CRISPR–Cas13d system alleviates disease-related phenotypes in Huntington's disease models. Nat. Neurosci. *26*, 27–38. https://doi.org/10.1038/s41593-022-01207-1.

15. Guo, Y., Han, L., Han, S., Tang, H., Wang, S., Cui, C., Chen, B., Li, H., and Shu, Y. (2022). Specific knockdown of Htra2 by CRISPR-CasRx prevents acquired sensorineural hearing loss in mice. Mol. Ther. Nucleic Acids *28*, 643–655. https://doi.org/10.1016/j.omtn.2022.04.014.

16. Zhou, C., Hu, X., Tang, C., Liu, W., Wang, S., Zhou, Y., Zhao, Q., Bo, Q., Shi, L., Sun, X., et al. (2020). CasRx-mediated RNA targeting prevents choroidal neovascularization in a mouse model of age-related macular degeneration. Natl. Sci. Rev. *7*, 835–837. https://doi.org/10.1093/nsr/nwaa033.

17. Buchman, A.B., Brogan, D.J., Sun, R., Yang, T., Hsu, P.D., and Akbari, O.S. (2020). Programmable RNA Targeting Using CasRx in Flies. CRISPR J. *3*, 164–176. https://doi.org/10.1089/crispr.2020.0018.

18. Huynh, N., Depner, N., Larson, R., and King-Jones, K. (2020). A versatile toolkit for CRISPR-Cas13-based RNA manipulation in Drosophila. Genome Biol. *21*, 279. https://doi.org/10.1186/s13059-020-02193-y.

19. Ai, Y., Liang, D., and Wilusz, J.E. (2022). CRISPR/Cas13 effectors have differing extents of off-target effects that limit their utility in eukaryotic cells. Nucleic Acids Res. *50*, e65. https://doi.org/10.1093/nar/gkac159.

20. Tong, H., Huang, J., Xiao, Q., He, B., Dong, X., Liu, Y., Yang, X., Han, D., Wang, Z., Wang, X., et al. (2023). High-fidelity Cas13 variants for targeted RNA degradation with minimal collateral effects. Nat. Biotechnol. *41*, 108–119. https://doi.org/10.1038/s41587-022-01419-7.

21. Wei, J., Lotfy, P., Faizi, K., Baungaard, S., Gibson, E., Wang, E., Slabodkin, H., Kinnaman, E., Chandrasekaran, S., Kitano, H., et al. (2023). Deep learning and CRISPR-Cas13d ortholog discovery for optimized RNA targeting. Cell Syst. *14*, 1087–1102.e13. https://doi.org/10.1016/j.cels.2023.11.006.

22. Shi, P., Murphy, M.R., Aparicio, A.O., Kesner, J.S., Fang, Z., Chen, Z., Trehan, A., Guo, Y., and Wu, X. (2023). Collateral activity of the CRISPR/RfxCas13d system in human cells. Commun. Biol. *6*, 334. https://doi.org/10.1038/s42003-023-04708-2.

23. Li, Z., Li, Z., Cheng, X., Wang, S., Wang, X., Ma, S., Lu, Z., Zhang, H., Zhao, W., Chen, Z., et al. (2024). Intrinsic targeting of host RNA by Cas13 constrains its utility. Nat. Biomed. Eng. *8*, 177–192. https://doi.org/10.1038/s41551-023-01109-y.

24. Li, Y., Xu, J., Guo, X., Li, Z., Cao, L., Liu, S., Guo, Y., Wang, G., Luo, Y., Zhang, Z., et al. (2023). The collateral activity of RfxCas13d can induce lethality in a RfxCas13d knock-in mouse model. Genome Biol. *24*, 20. https://doi.org/10.1186/s13059-023-02860-w.

25. Allen, H.N., Chaudhry, S., Hong, V.M., Lewter, L.A., Sinha, G.P., Carrasquillo, Y., Taylor, B.K., and Kolber, B.J. (2023). A Parabrachial-to-Amygdala Circuit That Determines Hemispheric Lateralization of Somatosensory Processing. Biol. Psychiatry *93*, 370–381. https://doi.org/10.1016/j.biopsych.2022.09.010.

26. van Gestel, M.A., van Erp, S., Sanders, L.E., Brans, M.A.D., Luijendijk, M.C.M., Merkestein, M., Pasterkamp, R.J., and Adan, R.A.H. (2014). shRNA-induced saturation of the microRNA pathway in the rat brain. Gene Ther. *21*, 205–211. https://doi.org/10.1038/gt.2013.76.

27. Baek, S.T., Kerjan, G., Bielas, S.L., Lee, J.E., Fenstermaker, A.G., Novarino, G., and Gleeson, J.G. (2014). Off-target effect of doublecortin family shRNA on neuronal migration associated with endogenous microRNA dysregulation. Neuron *82*, 1255–1262. https://doi.org/10.1016/j.neuron.2014.04.036.

28. Swiech, L., Heidenreich, M., Banerjee, A., Habib, N., Li, Y., Trombetta, J., Sur, M., and Zhang, F. (2015). In vivo interrogation of gene function in the mammalian brain using CRISPR-Cas9. Nat. Biotechnol. *33*, 102–106. https://doi.org/10.1038/nbt.3055.

29. Ai, H.W., Shaner, N.C., Cheng, Z., Tsien, R.Y., and Campbell, R.E. (2007). Exploration of new chromophore structures leads to the identification of improved blue fluorescent proteins. Biochemistry *46*, 5904–5910. https://doi.org/10.1021/bi700199g.

30. Wessels, H.H., Méndez-Mancilla, A., Guo, X., Legut, M., Daniloski, Z., and Sanjana, N.E. (2020). Massively parallel Cas13 screens reveal principles for guide RNA design. Nat. Biotechnol. *38*, 722–727. https://doi.org/10.1038/s41587-020-0456-9.

31. Ehlers, M.D. (2003). Activity level controls postsynaptic composition and signaling via the ubiquitin-proteasome system. Nat. Neurosci. *6*, 231–242. https://doi.org/10.1038/nn1013.

32. Su, X., Yao, X., Sun, Z., Han, Q., and Zhao, R.C. (2016). Optimization of Reference Genes for Normalization of Reverse Transcription Quantitative Real-Time Polymerase Chain Reaction Results in Senescence Study of Mesenchymal Stem Cells. Stem Cells Dev. *25*, 1355–1365. https://doi.org/10.1089/scd.2016.0031.

33. Chen, E.Y., Tan, C.M., Kou, Y., Duan, Q., Wang, Z., Meirelles, G.V., Clark, N.R., and Ma'ayan, A. (2013). Enrichr: interactive and collaborative HTML5 gene list enrichment analysis tool. BMC Bioinf. *14*, 128. https://doi.org/10.1186/1471-2105-14-128.

34. Kuleshov, M.V., Jones, M.R., Rouillard, A.D., Fernandez, N.F., Duan, Q., Wang, Z., Koplev, S., Jenkins, S.L., Jagodnik, K.M., Lachmann, A., et al. (2016). Enrichr: a comprehensive gene set enrichment analysis web server 2016 update. Nucleic Acids Res. *44*, W90–W97. https://doi.org/10.1093/nar/gkw377.

35. Xie, Z., Bailey, A., Kuleshov, M.V., Clarke, D.J.B., Evangelista, J.E., Jenkins, S.L., Lachmann, A., Wojciechowicz, M.L., Kropiwnicki, E., Jagodnik, K.M., et al. (2021). Gene Set Knowledge Discovery with Enrichr. Curr. Protoc. *1*, e90. https://doi.org/10.1002/cpz1.90.

36. Holehonnur, R., Luong, J.A., Chaturvedi, D., Ho, A., Lella, S.K., Hosek, M.P., and Ploski, J.E. (2014). Adeno-associated viral serotypes produce differing titers and differentially transduce neurons within the rat basal and lateral amygdala. BMC Neurosci. *15*, 28. https://doi.org/10.1186/1471-2202-15-28.

OMTM, Volume *33*

Supplemental information

Adeno-associated viral vector resource for the RNA-targeting Cas13d: A comparison of high-fidelity variants, DjCas13d and hfCas13d

Franklin Back, Alfredo Sandoval, Lily M. Vu, Veronica M. Hong, Amulya Bhaskara, Sierra R. Rodriguez, John T. O'Brien, Benedict J. Kolber, Sven Kroener, and Jonathan E. Ploski

`

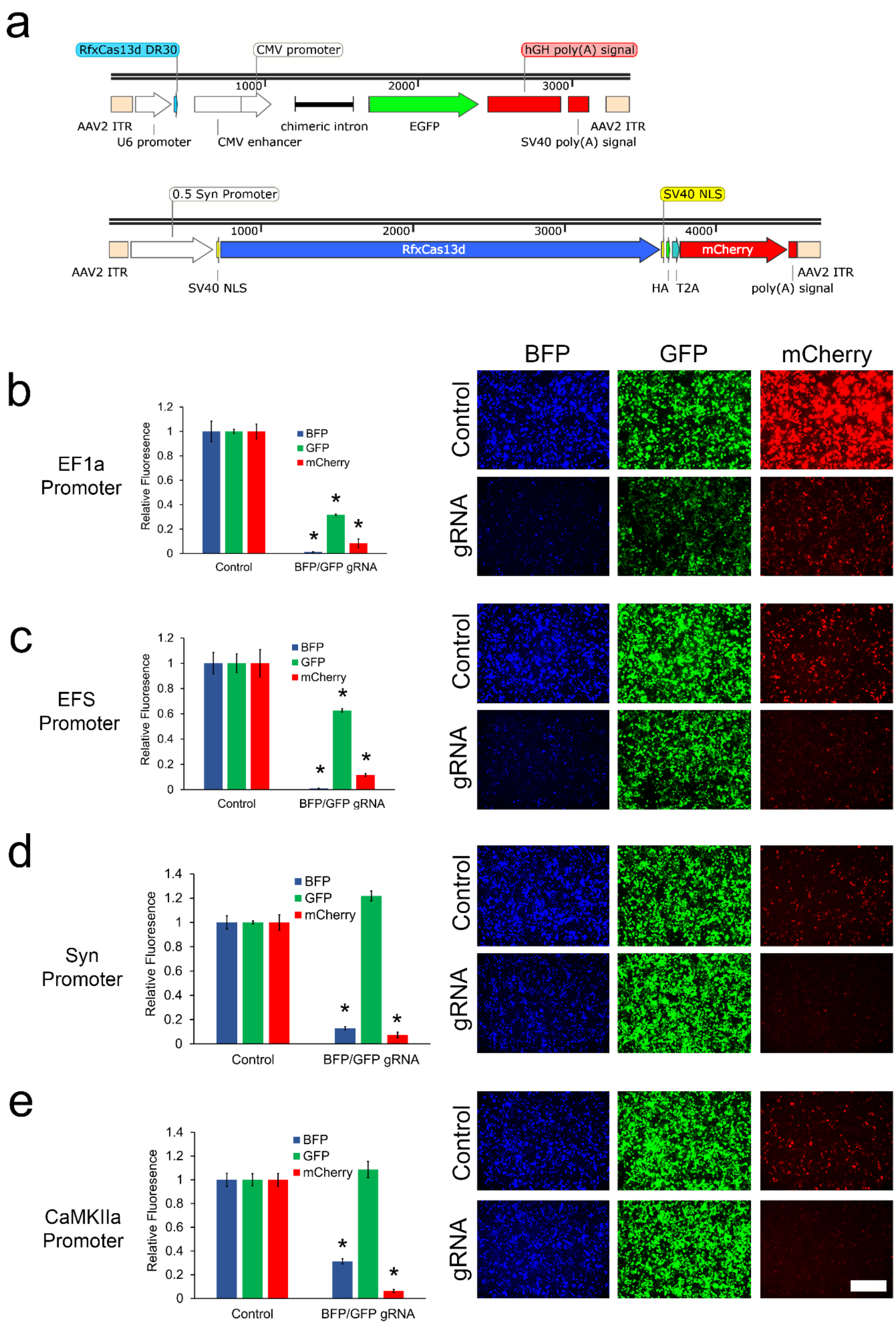
a
RfxCas13d DR30
CMV promoter
hGH poly(A) signal
1000
2000
3000
AAV2 ITR
U6 promoter
CMV enhancer
chimeric intron
EGFP
AAV2 ITR
SV40 poly(A) signal
0.5 Syn Promoter
SV40 NLS
1000
2000
3000
4000
RfxCas13d
mCherry
AAV2 ITR
SV40 NLS
HA
T2A
AAV2 ITR
poly(A) signal
BFP
GFP
mCherry
b
EF1a Promoter
Relative Fluoresence
Control
BFP/GFP gRNA
Control
gRNA
c
EFS Promoter
d
Syn Promoter
e
CaMKIIa Promoter

`

Figure S1: Demonstration of the functionality of AAV-based gRNA and CasRx (dual vector) plasmids in Neuro-2A cells. a.) Schematic of AAV vector maps. The first vector is designed to express a gRNA expression cassette using a U6 promoter and a GFP gene designed to be expressed from a CMV promoter. The second vector is designed to express a CasRx(RfxCas13d)-mCherry gene controlled from a synapsin promoter. Additional vectors were similarly designed to include an EF-1α, EF-1α short (EFS), or an alpha-CaMKII promoter, instead of a synapsin promoter to control CasRx expression (not shown). **b-e.)** Guide RNA plasmids containing a BFP/GFP targeting gRNA or non-targeting gRNA control plasmids were co-transfected with plasmids designed to express CasRx and nuclear-localized BFP, and 48 hours later the cells were imaged for BFP, GFP and mCherry epifluorescence. EF-1α, EFS, Synapsin, and alpha-CaMKII versions are shown respectively in b., c., d., and e. Quantification of data is depicted on the left side, representative images are presented on the right side. BFP/GFP gRNA-treated samples all exhibited a significant reduction in BFP levels compared to controls, but GFP levels were only lowered in experiments using the EF-1α and EFS CasRx plasmids. The levels of mCherry dropped in all BFP/GFP gRNA-treated conditions. Error bars = standard error of the mean. Four biological replicates ($n = 4$) were used for all experimental groups. Asterisk (*) indicates a significant difference compared to the respective control group (t-test, two-tailed, * = $p < 0.003$). Scale bar = 400 microns. Note: The EF-1α-CasRx-mCherry AAV vector genome is above the recommended packaging limit.

`

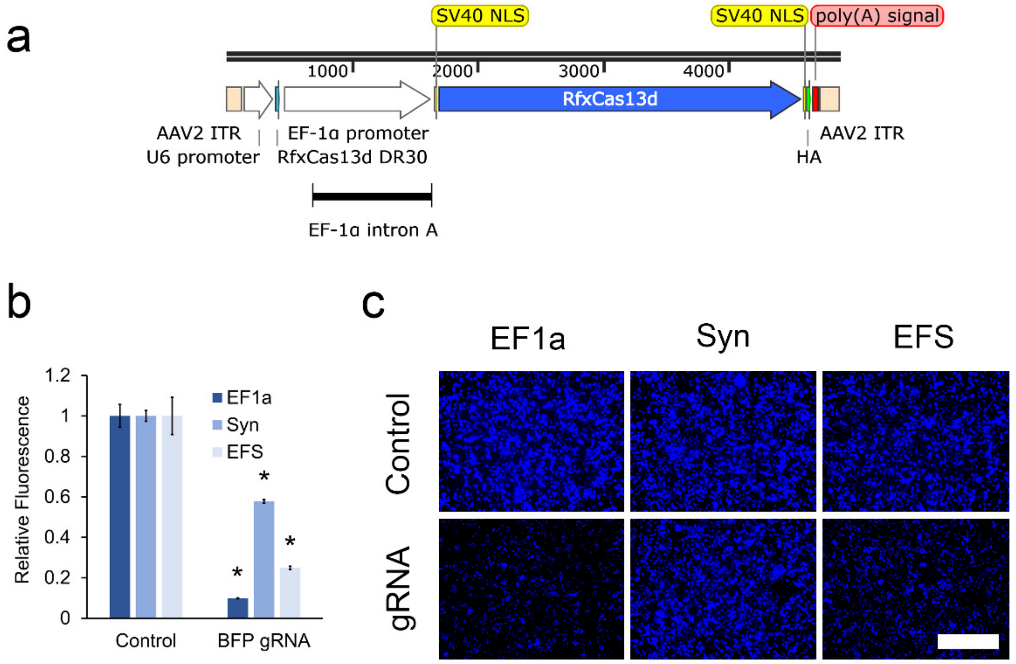

Figure S2: Demonstration of the functionality of AAV-based gRNA/CasRx (single vector) plasmids in Neuro-2A cells. a.) Schematic of AAV vector map designed to express a gRNA expression cassette using a U6 promoter and a CasRx(RfxCas13d) gene controlled from an EF-1α promoter. Additional vectors were similarly designed to include an EF-1α short (EFS) or synapsin promoter, instead of an EF-1α promoter to control CasRx expression (not shown). **b-c.)** Each of these single vector plasmids containing a BFP targeting gRNA or non-targeting gRNA control were co-transfected with plasmids designed to express nuclear-localized BFP, and 48 hours later the cells were imaged for BFP epifluorescence. Quantification of data depicted in b.), and representative images presented in c.). BFP gRNAs treated samples all exhibited a significant reduction in BFP levels compared to controls. Error bars = standard error of the mean. Four biological replicates (n = 4) were used for all experimental groups. Asterisk (*) indicates a significant difference compared to the respective control group (t-test, two-tailed, * = $p < 0.0002$). Scale bar = 450 microns.

`

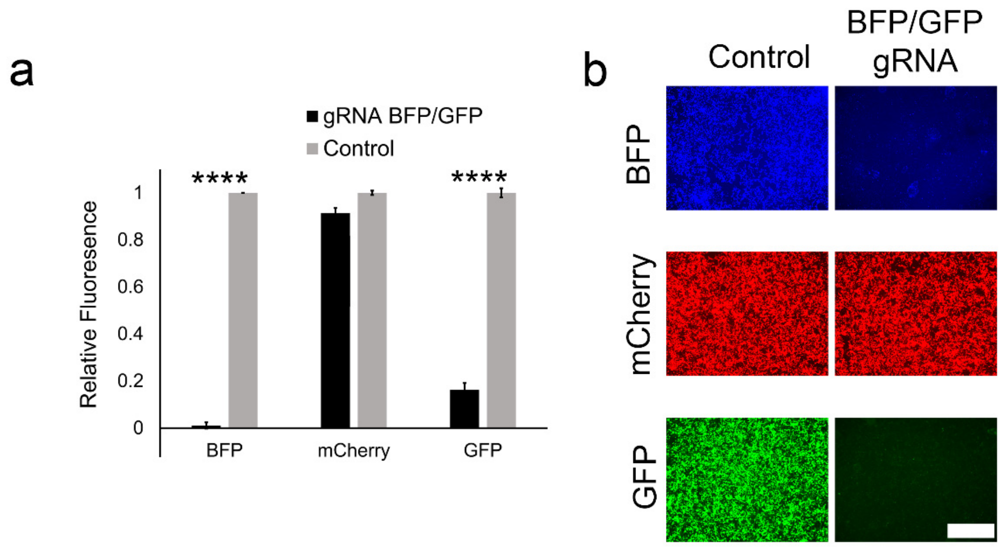

Figure S3: BFP/GFP targeting gRNA is not targeting mCherry. To ensure the BFP/GFP targeting gRNA is not also targeting mCherry, we co-transfected 293FT cells with a vector containing an EFS-CasRx gene, a BFP/GFP gRNA expression cassette, and either BFP, GFP, or mCherry encoding plasmids. Forty-eight hours later, the cells were imaged for BFP/GFP/mCherry expression. BFP and GFP fluorescence were significantly lower in samples that received the BFP/GFP targeting gRNA compared to control samples that received a non-targeting gRNA while mCherry levels remained high, indicating that the gRNA was not targeting mCherry. Data quantitation is depicted in a.) and representative images are depicted in b.). All groups contained 4-6 biological replicates ($n = 4\text{-}6$). T-test, two-tailed, **** = $p < 1E\text{-}9$ compared to the respective control group. Error bars = standard error of the mean. Scale bar = 400 microns.

`

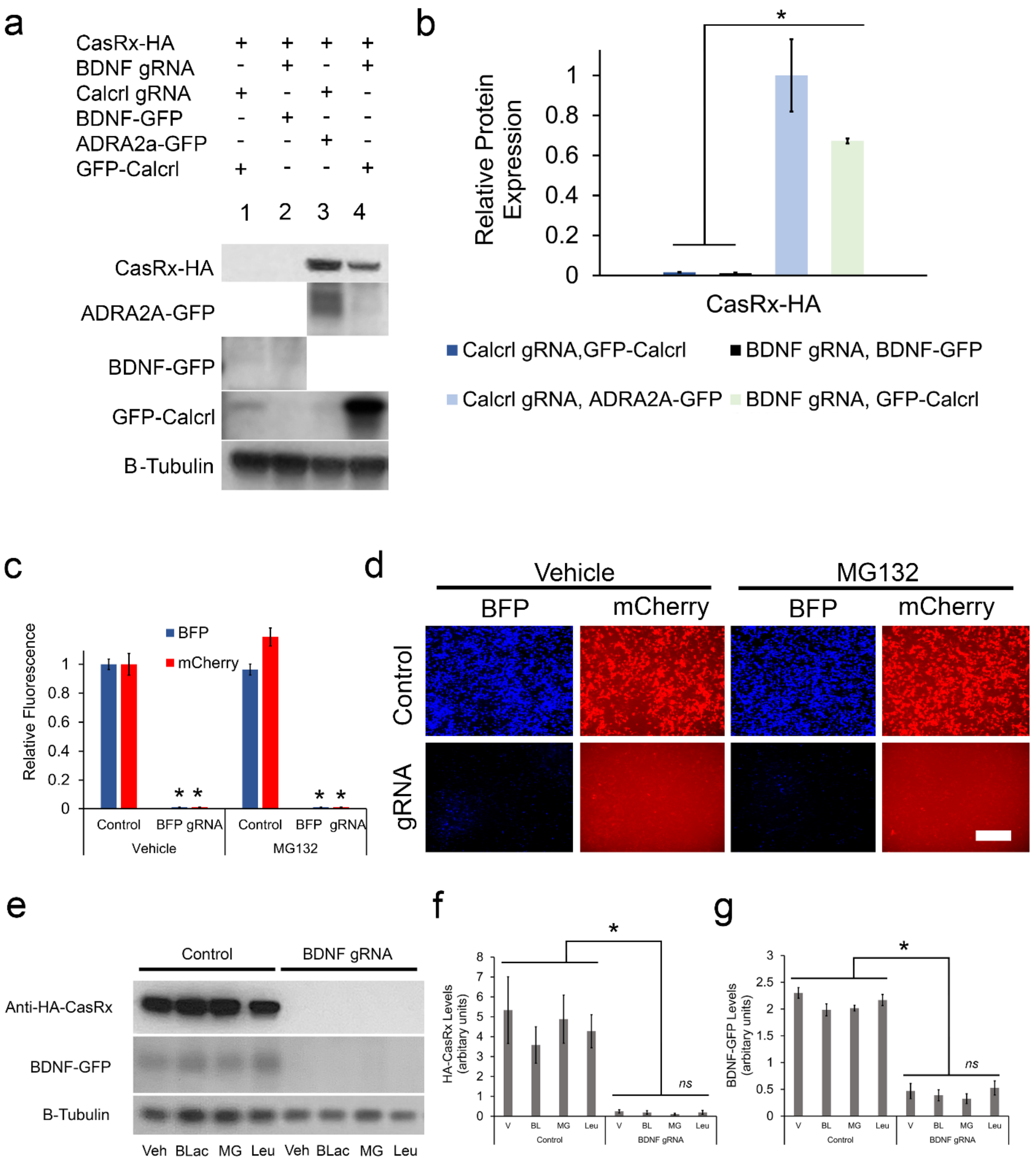

a
CasRx-HA + + + +
BDNF gRNA - + - +
Calcrl gRNA + - + -
BDNF-GFP - + - -
ADRA2a-GFP - - + -
GFP-Calcrl + - - +
1 2 3 4
CasRx-HA
ADRA2A-GFP
BDNF-GFP
GFP-Calcrl
B-Tubulin
b
Relative Protein Expression
CasRx-HA
Calcrl gRNA,GFP-Calcrl
BDNF gRNA, BDNF-GFP
Calcrl gRNA, ADRA2A-GFP
BDNF gRNA, GFP-Calcrl
c
Relative Fluorescence
BFP
mCherry
Control
BFP gRNA
Vehicle
MG132
d
Vehicle
MG132
BFP
mCherry
Control
gRNA
e
Control
BDNF gRNA
Anti-HA-CasRx
BDNF-GFP
B-Tubulin
Veh BLac MG Leu Veh BLac MG Leu
f
HA-CasRx Levels (arbitary units)
V BL MG Leu
Control
BDNF gRNA
ns
g
BDNF-GFP Levels (arbitary units)
ns

`

Figure S4: CasRx protein levels become significantly depleted when CasRx is co-expressed with a gRNA and its intended target and this is not influenced by the ubiquitin-proteasome system. a-b.) 293FT cells were transfected with plasmids encoding EFS-CasRx, gRNAs designed to target *BDNF*, or *Calcrl*, and plasmids designed to express rat BDNF-GFP, ADRA2A-GFP, or GFP-*Calcrl*. Forty-eight hours later, the cells were harvested and examined via western blotting for CasRx, ADRA2A-GFP and GFP-*Calcrl* protein levels. When *Calcrl* gRNAs and GFP-*Calcrl* (lane 1) or *BDNF* gRNAs and BDNF-GFP (lane 2), were co-expressed, CasRx protein levels were significantly lowered. However, CasRx levels remained high when *Calcrl* gRNAs and ADRA2A-GFP (lane 3) or *BDNF* gRNAs and GFP-*Calcrl* (lane 4), were co-expressed. CasRx protein levels were significantly depleted when CasRx was co-expressed with a gRNA and its intended target (ANOVA with Tukey pairwise comparisons, * = $p < 0.004$). Note that BDNF protein is not present in lane two because it is degraded. Each group contained 3 biological replicates (n = 3). Error bars = standard error of the mean. HA-CasRx and GFP protein levels were normalized to Beta-Tubulin levels for each sample. **(c-d).** 293FT cells were transfected with plasmids encoding CasRx-mCherry, BFP, and BFP gRNA expression cassettes or control gRNA expression cassettes, and the cells were either treated with vehicle or the ubiquitin-proteasome inhibitor, MG132. Forty-eight hours later, the cells were imaged for BFP and mCherry epifluorescence. BFP and mCherry fluorescence dropped in samples that were treated with BFP gRNAs, but not controls, and the treatment with MG132 had no influence on these levels. Two-way ANOVA revealed a significant effect for BFP gRNA on BFP and mCherry levels (* = $p < 1E\text{-}9$), however, there was no interaction for MG132 treatment ($p > 0.05$). Quantified data are shown in c.) and representative images are depicted in d.). Four biological replicates for all groups (n = 4). Scale bar = 375 microns. **(e-g)**. 293FT cells were transfected with plasmids encoding CasRx, BDNF-GFP, and *BDNF* gRNAs or control gRNAs expression cassettes and treated cells with either vehicle (Veh or V) or the ubiquitin-proteasome inhibitors, MG132 (MG), or clasto-Lactacystin β-lactone (BLac or BL) or the lysosomal inhibitor leupeptin (Leu). Forty-eight hours later, the cells were harvested, and protein levels for CasRx and BDNF-GFP were examined via western blotting. CasRx and BDNF-GFP levels were significantly lowered in the cells that were treated with *BDNF* gRNAs compared to controls (Two-way ANOVA, * $p < 0.0000003$), and none of the inhibitors influenced these protein levels (Two-way ANOVA $p > 0.3$ = *ns*). Four biological replicates for all groups (n = 4). Error bars = standard error of the mean.

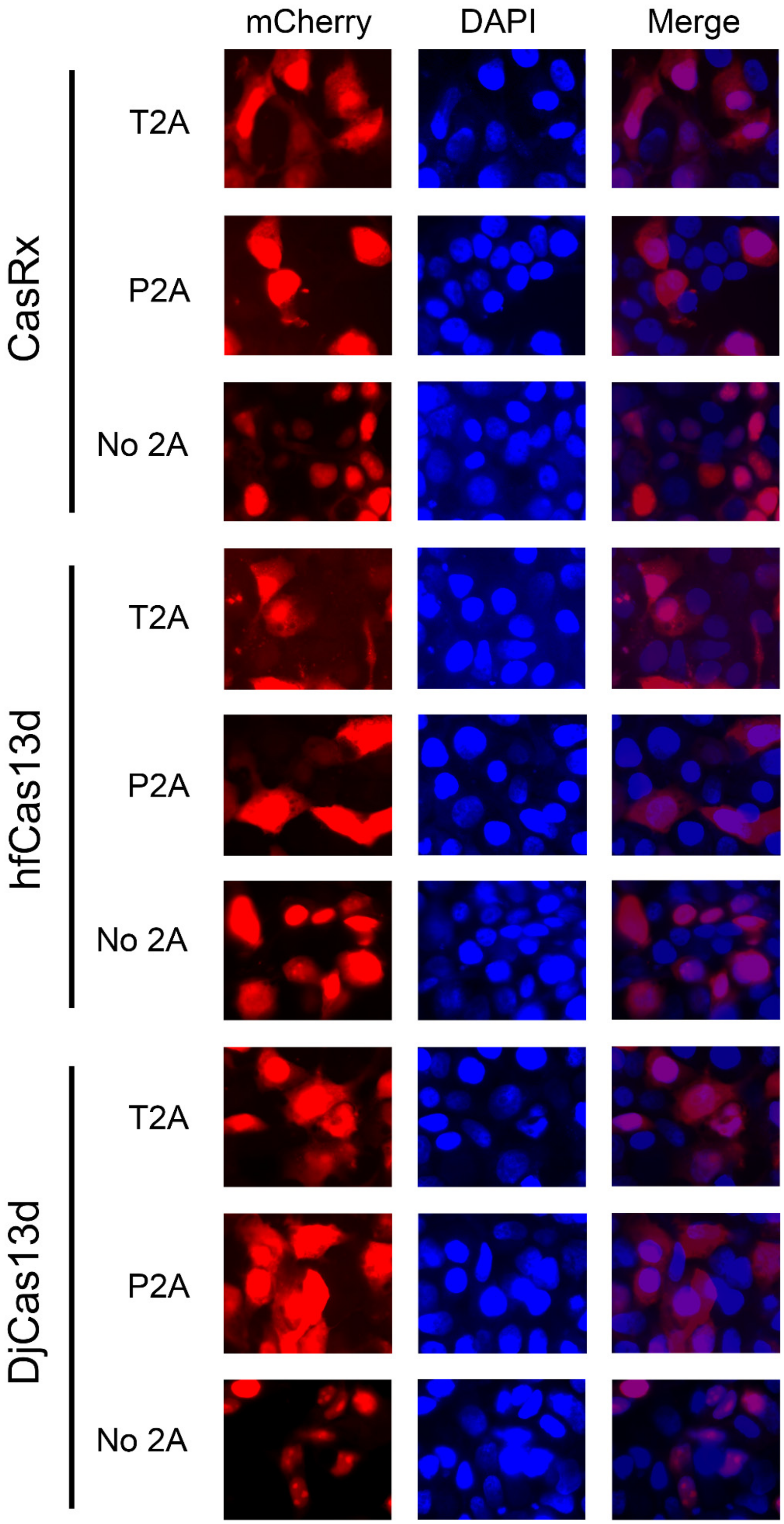
mCherry
DAPI
Merge
CasRx
T2A
P2A
No 2A
hfCas13d
T2A
P2A
No 2A
DjCas13d
T2A
P2A
No 2A

`

Figure S5: Cas13d/mCherry protein cellular localization. Plasmids designed to encode three different variants of Cas13d that contain either a T2A-mCherry sequence, a P2A-mCherry sequence or don't have a 2A sequence, but are designed to be a fusion protein with mCherry, were transfected into 293FT cells. Forty-eight hours later the cells were imaged for mCherry and DAPI epifluorescence to observe the cellular localization of mCherry. From left to right, the panels are mCherry, DAPI and the merged signal between the two to clearly indicate the cellular localization of mCherry. In all cases there is strong nuclear localization of mCherry. For samples that contain 2A sequences, there is also significant cytoplasmic localization of mCherry. Samples that do not contain 2A sequences but are instead Cas13-Cherry fusions, show virtually only nuclear localization except in cases of high expression. Representative images are shown for all samples.

`

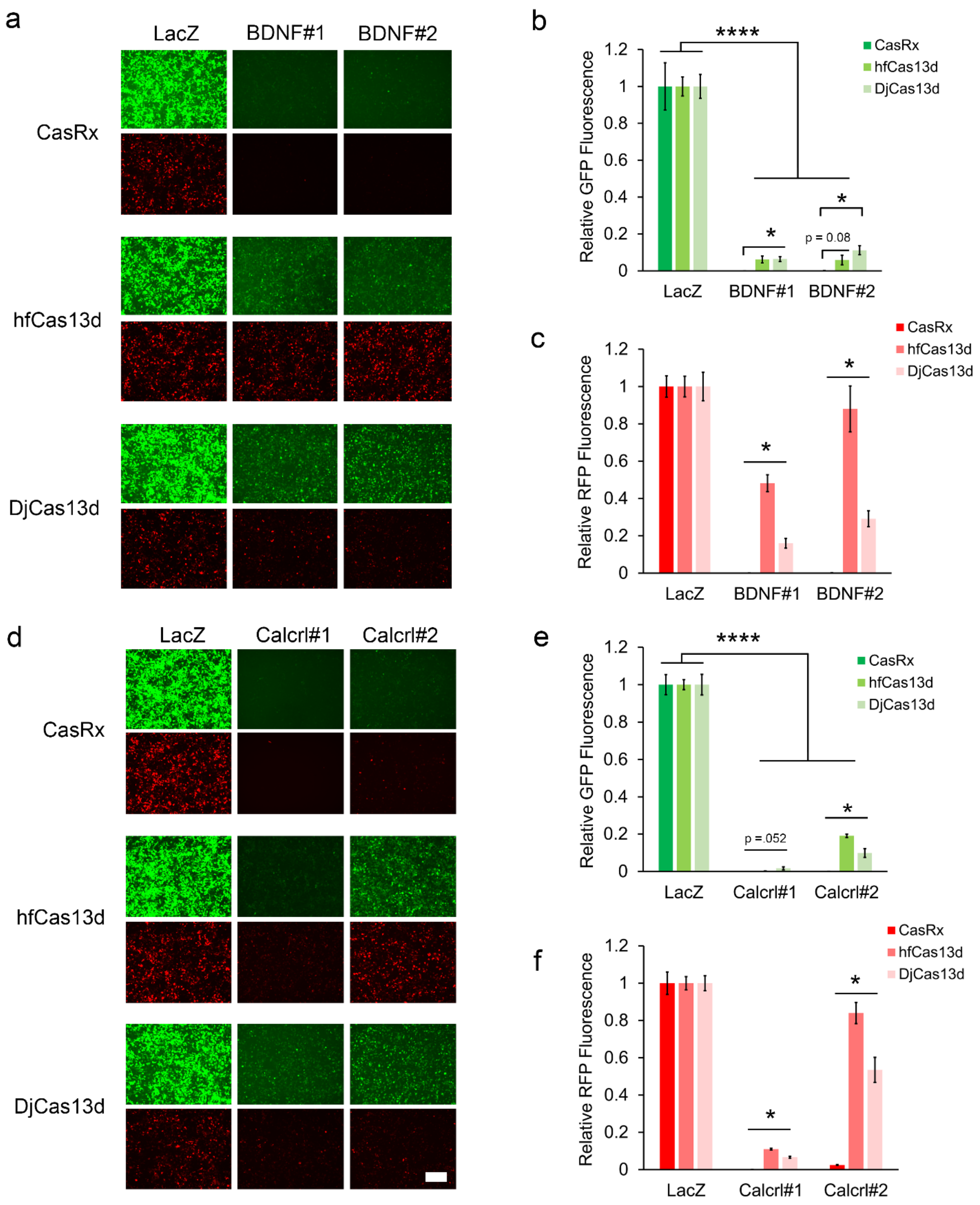

Figure S6: Variants of Cas13d knock down ectopically-expressed genes efficiently. (a). 293FT cells were transfected with Cas13d encoding vectors, a GFP-*BDNF* encoding plasmid and two different gRNA expression cassettes, *BDNF* 1 and 2, or a gRNA expression cassette designed to target LacZ as a control. Forty-eight hours post-transfection the cells were imaged for GFP and mCherry(RFP) epifluorescence. Representative images are shown. **(b).** Quantification of

`

images revealed that treatment with both *BDNF* gRNAs resulted in robust knockdown of GFP-*BDNF* for each of the Cas13d variants compared to LacZ controls (**** = ANOVA, p < 0.000006; Fisher's LSD, p < 0.000005). There was a significant difference in GFP-*BDNF* knockdown for *BDNF* 1 among the Cas13d variants, with CasRx outperforming the other Cas13d variants (* = ANOVA, p = 0.0103; Fisher's LSD, p < 0.008059). There was a significant difference in GFP-*BDNF* knockdown for *BDNF* 2 between CasRx and DjCas13d, (* = ANOVA, p < 0.0134; Fisher's LSD, p = 0.0042). Four biological replicates for all groups (n = 4). **(c).** Both DjCas13d and CasRx groups, for both *BDNF* 1 and 2, exhibited a significant reduction in RFP levels compared to LacZ controls, (ANOVA, p < 0.000003; Fisher's LSD, p < 0.000006), however, RFP levels were only significantly different from LacZ controls for hfCas13d *BDNF* 1 (ANOVA, p = 0.003991; Fisher's LSD, p < 0.001598), but not hfCas13d *BDNF* 2 (p = 0.3296). RFP levels for the hfCas13d, *BDNF* 1 and 2 groups were significantly higher than the DjCas13d and CasRx, *BDNF* 1 and 2 groups (* = ANOVA, p < 0.000055; Fisher's LSD, p < 0.023700). Four biological replicates for all groups (n = 4). **(d).** 293FT cells were transfected with either of these Cas13d encoding vectors, a GFP-*Calcrl* encoding plasmid and two different gRNA expression cassettes, *Calcrl* 1 and 2, or a gRNA expression cassette designed to target LacZ as a control. Forty-eight hours post transfection the cells were imaged for GFP and mCherry(RFP) epifluorescence. Representative images shown. **(e).** Quantification of images revealed that treatment with both *Calcrl* gRNAs resulted in robust knockdown of GFP-*Calcrl* for each of the Cas13d variants compared to LacZ controls (**** = ANOVA, p < 1.39E-08; Fisher's LSD, p < 1.96E-08). There was not a significant difference in GFP-*Calcrl* knockdown for *Calcrl* 1 among the Cas13d variants, (ANOVA, p = 0.052). There was a significant difference in GFP-*Calcrl* knockdown for *Calcrl* 2 among the Cas13d variants, with CasRx outperforming the other Cas13d variants (* = ANOVA, p = 0.000024; Fisher's LSD, p < 0.000966). The use of DjCas13d led to a significant knockdown of GFP-*Calcrl* compared to hfCas13d (p < 0.001449). Four biological replicates for all groups (n = 4). **(f).** Each of the Cas13d variants, for both *Calcrl* 1 and 2, exhibited a significant reduction in RFP levels compared to LacZ controls, (** = ANOVA, p < 0.000001; Fisher's LSD, p < 0.016729). RFP levels for the hfCas13d, *Calcrl* 1 and 2 groups were significantly higher than the DjCas13d and CasRx, *Calcrl* 1 and 2 groups (* = ANOVA, p < 0.000004; Fisher's LSD, p < 0.002160). Four biological replicates for all groups (n = 4). Error bars = standard error of the mean. Scale bar = 325 microns.

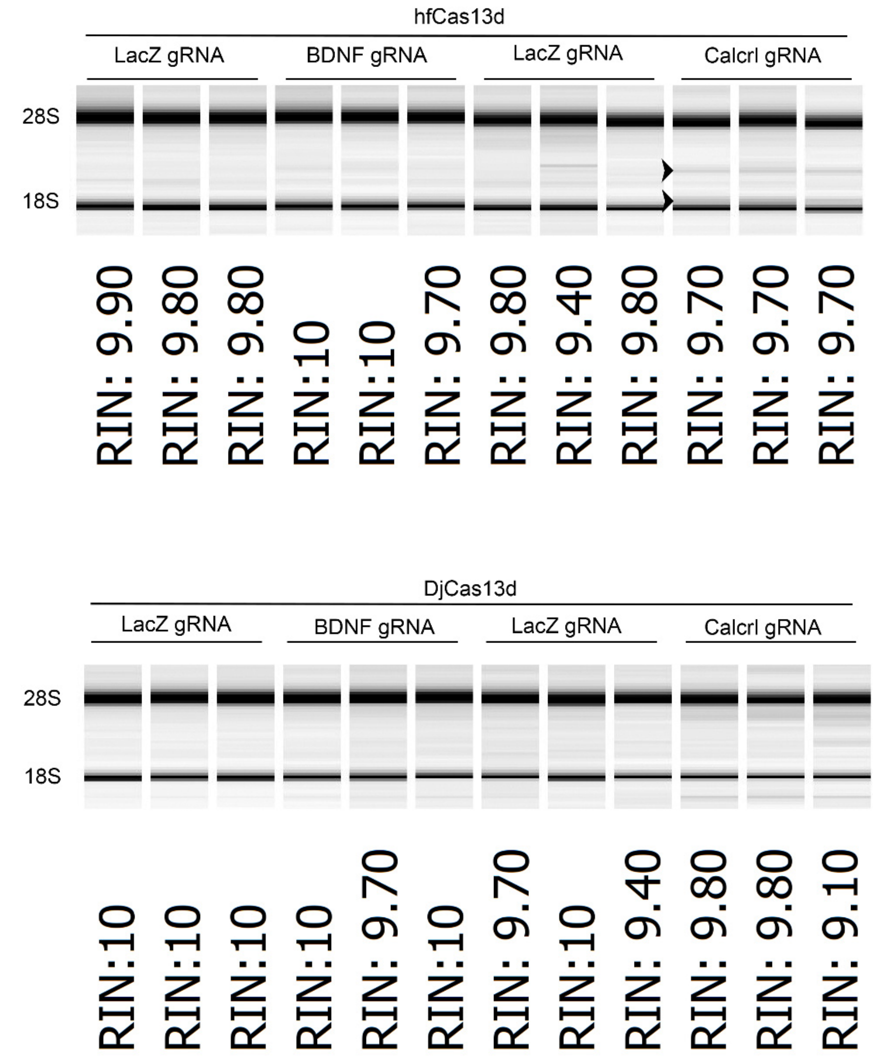

Figure S7:

RNA analysis via Agilent bioanalyzer for total RNA samples that were used in experiments depicted in Figures S6 and Figure 4. Arrows point to the two degraded RNA bands seen in the hfCas13d/*Calcrl* gRNA samples. No other samples show this stereotyped pattern of RNA degradation. These findings are consistent with the data presented in Figure 3. RNA Integrity Numbers (RINs) are shown for each sample.

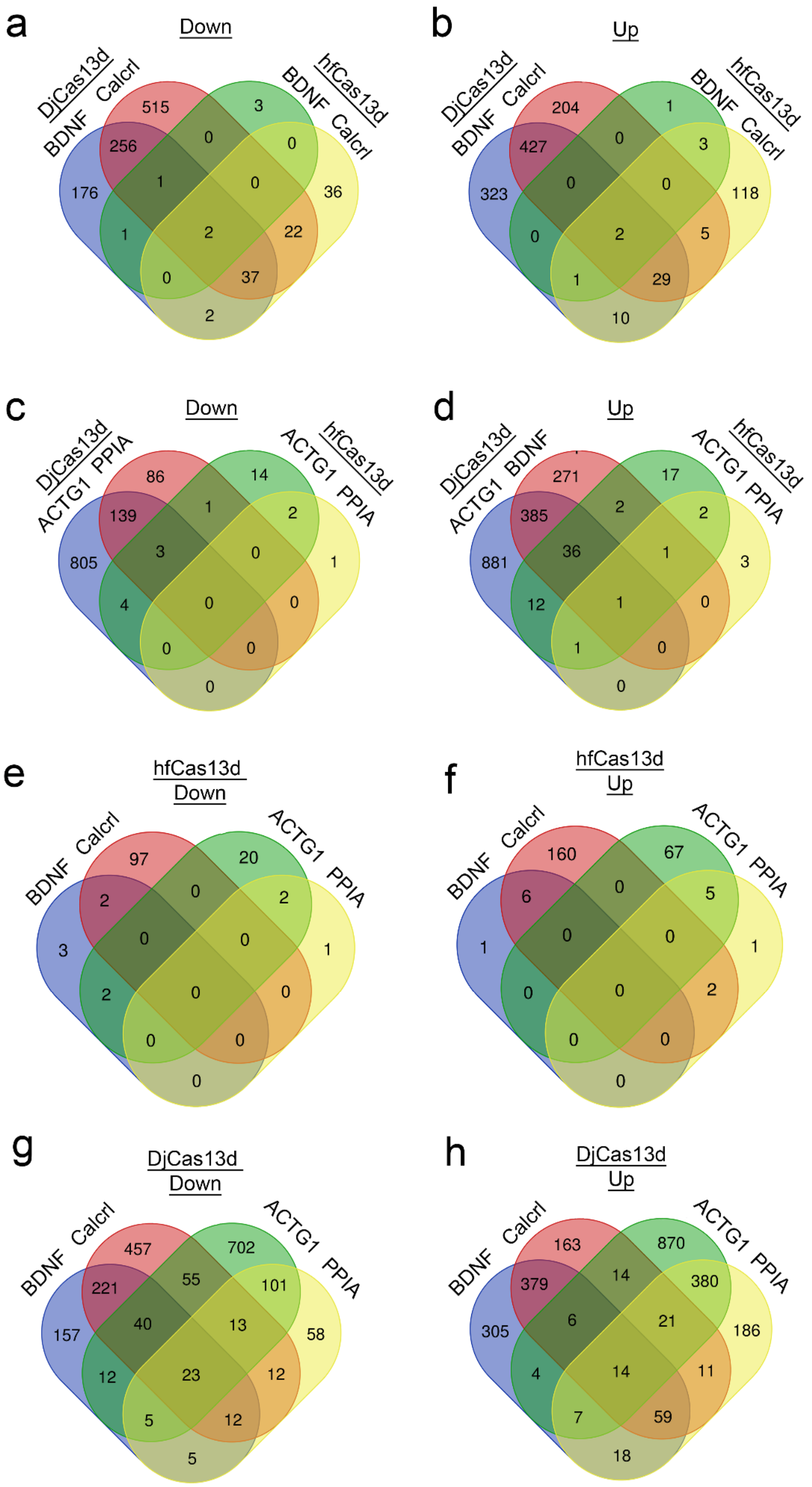
a
Down
DjCas13d
BDNF Calcrl
hfCas13d
BDNF Calcrl
515
3
256
0
0
176
1
0
36
1
2
22
0
37
2
b
Up
DjCas13d
BDNF Calcrl
hfCas13d
BDNF Calcrl
204
1
427
0
3
323
0
0
118
0
2
5
1
29
10
c
Down
DjCas13d
ACTG1 PPIA
hfCas13d
ACTG1 PPIA
86
14
139
1
2
805
3
0
1
4
0
0
0
0
0
d
Up
DjCas13d
ACTG1 BDNF
hfCas13d
ACTG1 PPIA
271
17
385
2
2
881
36
1
3
12
1
0
1
0
0
e
hfCas13d
Down
BDNF Calcrl
ACTG1 PPIA
97
20
2
0
2
3
0
0
1
2
0
0
0
0
0
f
hfCas13d
Up
BDNF Calcrl
ACTG1 PPIA
160
67
6
0
5
1
0
0
1
0
0
2
0
0
0
g
DjCas13d
Down
BDNF Calcrl
ACTG1 PPIA
457
702
221
55
101
157
40
13
58
12
23
12
5
12
5
h
DjCas13d
Up
BDNF Calcrl
ACTG1 PPIA
163
870
379
14
380
305
6
21
186
4
14
11
7
59
18

`

Figure S8: Venn diagrams illustrating the similarities and differences of dysregulated genes among several RNAseq experiments.

(a). Venn diagram showing the overlap of downregulated genes from 4 RNAseq experiments (see Figure 4), designed to target GFP-*BDNF* or GFP-*Calcrl* with either DjCas13d or hfCas13d. (**b.**) Venn diagram showing the overlap of upregulated genes from 4 RNAseq experiments (see Figure 4), designed to target GFP-*BDNF* or GFP-*Calcrl* with either DjCas13d or hfCas13d. **(c).** Venn diagram showing the overlap of downregulated genes from 4 RNAseq experiments (see Figure 5), designed to target *ACTG1* or *PPIA* with either DjCas13d or hfCas13d. (**d.**) Venn diagram showing the overlap of upregulated genes from 4 RNAseq experiments (see Figure 5), designed to target *ACTG1* or *PPIA* with either DjCas13d or hfCas13d. **(e, f).** Venn diagrams showing the overlap of down and upregulated genes from 4 RNAseq experiments (see Figures 4 and 5), designed to target GFP-*BDNF*, GFP-*Calcrl*, *ACTG1* or *PPIA* with hfCas13d. **(g, h).** Venn diagrams showing the overlap of down and upregulated genes from 4 RNAseq experiments (see Figures 4 and 5), designed to target GFP-*BDNF*, GFP-*Calcrl*, *ACTG1* or *PPIA* with DjCas13d.

`

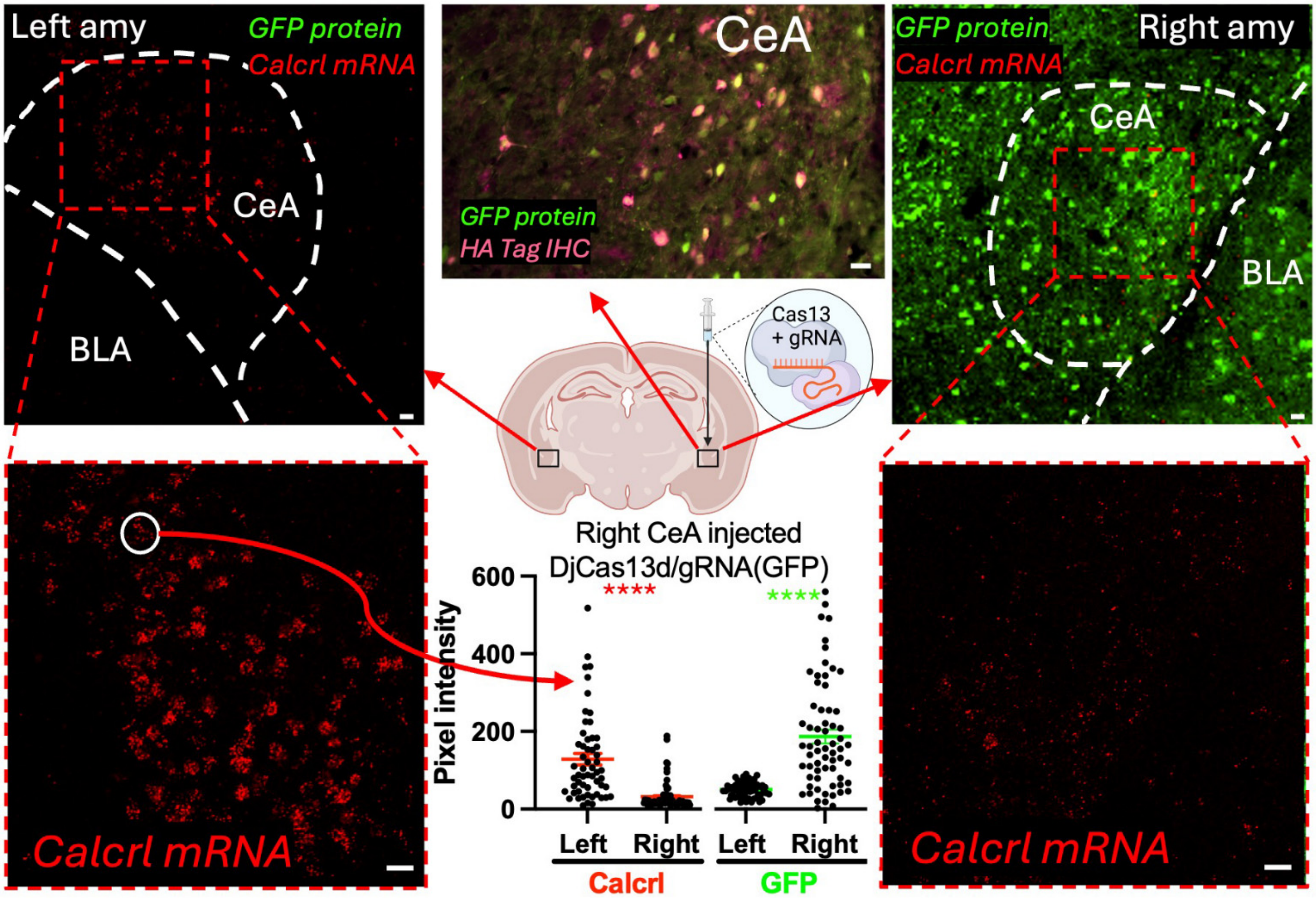

Figure S9: In Vivo Disruption of *Calcrl* mRNA with DjCas13d in mice. The right central amygdala (CeA) of a mouse was injected with AAV-DjCas13d-HA and AAV-gRNA*Calcrl*-GFP. Central top image shows >85% co-localization of GFP (gRNA vector) and HA tag IHC (DjCas13d protein). The amount of *Calcrl* mRNA was decreased in the right CeA (bottom right 400 x image) compared to the uninjected left CeA (bottom left 400 x image) as quantified in the bottom central graph. Quantification was completed on all cells in a representative image for *Calcrl* and for GFP (tagged to gRNA). Scale bars = 20um ****P<0.0001 t-test between sides.

`

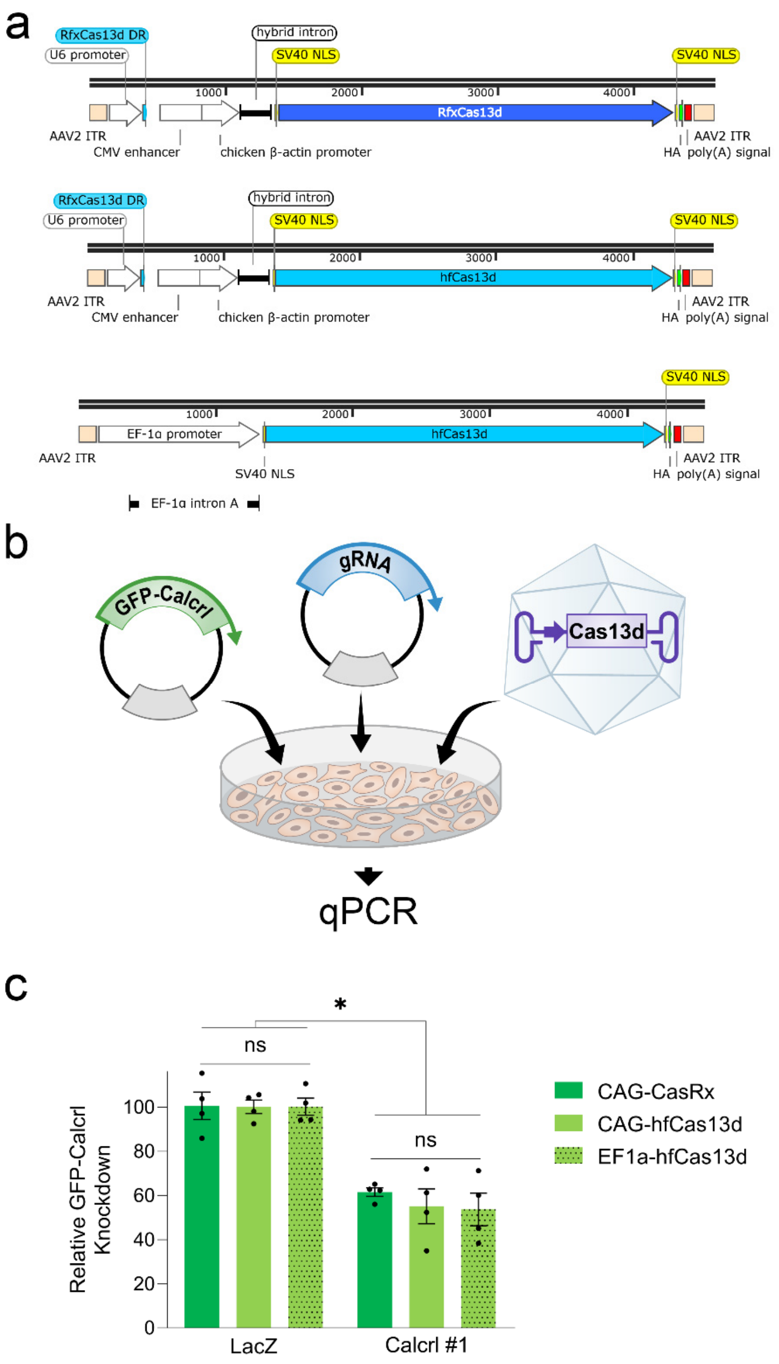

Figure S10: AAVs encoding Cas13d show functionality with different promoters

(a). Schematic of AAV vector maps depicting AAVs designed to express CasRx and hfCas13d, from a CAG promoter. These vectors also contain a gRNA expression cassette upstream of the Cas13d gene. The bottom vector is an AAV designed to express hfCas13d from an Ef1a promoter. This vector does not have a gRNA expression cassette. **(b,c).** Graphic describing the outline of the experiment. 293FT cells were transfected with a GFP-*Calcrl* encoding plasmid and a gRNA expression cassette, *Calcrl* 1, or a gRNA expression cassette designed to target LacZ as a control. The cells were also transduced with Cas13d encoding viruses depicted in (a.). Seventy-two hours post-transfection/transduction the samples were processed for qPCR to examine GFP mRNA levels. Quantitative PCR revealed GFP was significantly knocked down compared to the LacZ control group demonstrating the functionality of these viruses, two-way ANOVA (* = $p < 0.05$). Error bars = standard error of the mean.

`

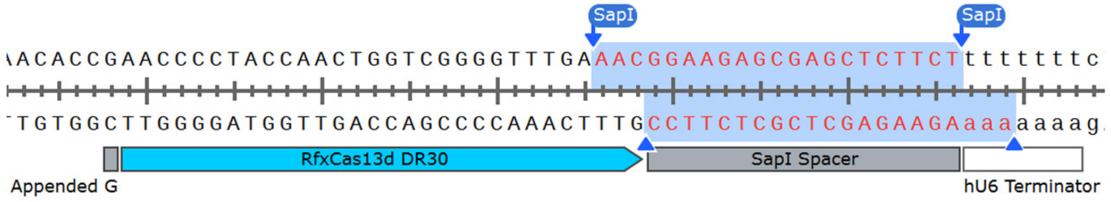

gRNA Name	Sequence (5' - 3')
GFP/BFP Top	aacTTGTACTCCAGCTTGTGCCCCAG
GFP/BFP Bot	aaaCTGGGGCACAAGCTGGAGTACAA
BDNF1 1 Top	aacAATAACCATAGTAAGGAAAAGGA
BDNF1 1 Bot	aaaTCCTTTTCCTTACTATGGTTATT
BDNF1 2 Top	aacACGTGCTCAAAAGTGTCAGCCAG
BDNF1 2 Bot	aaaCTGGCTGACACTTTTGAGCACGT
Calcrl 1 Top	aacGTAACATTCATATTGAGCCGTCA
Calcrl 1 Bot	aaaTGACGGCTCAATATGAATGTTAC
Calcrl 2 Top	aacCAAAGCAGCACAAATCGGACCAT
Calcrl 2 Bot	aaaATGGTCCGATTTGTGCTGCTTTG
ACTG1 Top	aacACTTCAGGGTCAGGATGCCACGCTTGCTCT
ACTG1 Bot	aaaAGAGCAAGCGTGGCATCCTGACCCTGAAGT
PPIA Top	aacAAACACCACATGCTTGCCATCCAACCACTC
PPIA Bot	aaaGAGTGGTTGGATGGCAAGCATGTGGTGTTT
LacZ Top	aacCGTCTGGCCTTCCTGTAGCCAGCTTTCATC
LacZ Bot	aaaGATGAAAGCTGGCTACAGGAAGGCCAGACG

Figure S11: Guide RNA composition for all experiments. Top: Image depicts the SapI sites used to ligate the gRNAs into the gRNA expression cassette. Bottom: Oligonucleotides used for all gRNAs used in this study. Lowercase nucleotides in red are the overhang sequences used to ligate into the SapI site of the gRNA expression cassette.

Supplemental Table Legends

Table S1: Dysregulated genes identified in each RNAseq experiment were subjected to ontology analysis to determine if specific classes of genes were enriched. Each RNAseq experiment is labeled in bold and underline and is referred to as either Dj or hf, referring to DjCas13d or hfCas13d; up or down referring to if the gene list was from the up or downregulated list and the name of the target gene. If genes were identified to be enriched in either the biological, cellular, or molecular ontological classifications, the classification is listed with the adjacent p-value and the genes that are part of each classification. If no classification met the adjusted p-value of > 0.05, then “nothing” is listed.

`

Table S2: Lists of genes that occur in at least 3 out of 4 RNAseq comparisons made for each comparison in Figure S8. On the left most, column is a description of what is contained in the adjacent rows: Relevant figure refers to the portion of Figure S8 the data refers to. Groups refers to which RNAseq groups the genes are co-dysregulated in. # of genes refers to the number of genes that were part of the comparison and match the associated portion of the Venn diagrams displayed in Figure S8. Genes refers to the specific genes that were found in the group. Each specific experimental comparison is highlighted in alternating dark or light gray highlight, to help differentiate the separate experimental comparisons.

Table S3: Ontology analysis for genes that were dysregulated in at least 3 out of the 4 RNAseq experiments depicted in each of the comparisons from Figure S8. The table is organized with biological and cellular ontological terms on the left. The adjusted p-value for each term in the middle and the genes that were identified in the genes lists that are part of each ontological category are listed on the right. If data in not presented for a particular comparison, it's because no genes were identified above chance or the experiment did not have any genes that occurred in the 3 out of 4 RNAseq experiments such as the case for comparisons made in Figure S8, e and f.

Table S4: Potential off-targets for each gRNA that were found to be in differentially expressed gene lists. Headings for each column describe what information the table contains.

Table S5: A list of each plasmid described in this study. Including the name of the plasmid, Addgene # if available, the ITR to ITR length of the viral genome in base pairs and which experimental figure the plasmid was used in. Note all the AAV plasmids contain viral genomes within the optimal packaging limit except for 1 which is highlighted in red. All the plasmids were created by the Ploski Lab except for the EF1a-mCherry plasmid.
